# Amine oxide lipid nanoparticles for delivery of cyclic dinucleotides

**DOI:** 10.1093/nsr/nwag340

**Published:** 2026-06-08

**Authors:** Yao Chen, Yanfeng Yang, Feng Xiao, Qian Wang, Shengbo Chen, Jia’an Li, Saijie Wang, Yunhan Zhang, Jun Wu, Xingyu Jiang

**Affiliations:** Shenzhen Key Laboratory of Smart Healthcare Engineering, Guangdong Provincial Key Laboratory of Advanced Biomaterials, Department of Biomedical Engineering, Southern University of Science and Technology, Shenzhen 518055, China; Bioscience and Biomedical Engineering Thrust, Hong Kong University of Science and Technology (Guangzhou), Guangzhou 511458, China; Shenzhen Key Laboratory of Smart Healthcare Engineering, Guangdong Provincial Key Laboratory of Advanced Biomaterials, Department of Biomedical Engineering, Southern University of Science and Technology, Shenzhen 518055, China; Shenzhen Key Laboratory of Smart Healthcare Engineering, Guangdong Provincial Key Laboratory of Advanced Biomaterials, Department of Biomedical Engineering, Southern University of Science and Technology, Shenzhen 518055, China; Shenzhen Key Laboratory of Smart Healthcare Engineering, Guangdong Provincial Key Laboratory of Advanced Biomaterials, Department of Biomedical Engineering, Southern University of Science and Technology, Shenzhen 518055, China; Shenzhen Key Laboratory of Smart Healthcare Engineering, Guangdong Provincial Key Laboratory of Advanced Biomaterials, Department of Biomedical Engineering, Southern University of Science and Technology, Shenzhen 518055, China; Shenzhen Key Laboratory of Smart Healthcare Engineering, Guangdong Provincial Key Laboratory of Advanced Biomaterials, Department of Biomedical Engineering, Southern University of Science and Technology, Shenzhen 518055, China; Shenzhen Key Laboratory of Smart Healthcare Engineering, Guangdong Provincial Key Laboratory of Advanced Biomaterials, Department of Biomedical Engineering, Southern University of Science and Technology, Shenzhen 518055, China; Shenzhen Key Laboratory of Smart Healthcare Engineering, Guangdong Provincial Key Laboratory of Advanced Biomaterials, Department of Biomedical Engineering, Southern University of Science and Technology, Shenzhen 518055, China; Bioscience and Biomedical Engineering Thrust, Hong Kong University of Science and Technology (Guangzhou), Guangzhou 511458, China; Shenzhen Key Laboratory of Smart Healthcare Engineering, Guangdong Provincial Key Laboratory of Advanced Biomaterials, Department of Biomedical Engineering, Southern University of Science and Technology, Shenzhen 518055, China

**Keywords:** lipid nanoparticle, amine oxide lipid, STING agonist, tumor immunotherapy, molecular self-assembly

## Abstract

The clinical potential of cyclic dinucleotides (CDNs) as potent agonists of the stimulator of interferon gene (STING) has been hampered by the lack of robust delivery systems. While lipid nanoparticles (LNPs) excel with large nucleic acids, they are unsuitable for delivering small, zwitterionic and highly hydrophilic CDNs. Here, we find that the tertiary amine oxide lipid (AOL) promotes stable encapsulation of CDNs into LNP through enhanced hydrogen bonding and electrostatic interactions, resulting in ultra-high encapsulation efficiency of 86.8%. Compared to cationic liposomes, AOL-incorporating LNP exhibits superior tumor penetration and cytosolic cargo delivery, eliciting broad and potent intratumoral STING activation. In line with growing emphasis on combination therapy in oncology, we further incorporate lipidated mitoxantrone in the LNP as an inducer of immunogenic cell death to enhance tumor antigen presentation and STING-driven immunotherapy. The obtained LNP provokes potent immunostimulatory and antitumor effects in multiple challenging animal models, including triple-negative breast cancer, pancreatic cancer and melanoma.

## Teaser

A tertiary amine oxide-based lipid interacts with cyclic dinucleotides, natural agonists of stimulator of interferon gene (STING), through hydrogen bonding and electrostatic forces, enabling an efficient lipid nanoparticle delivery system that potentiates STING-based anti-tumor immunity.

## INTRODUCTION

Nucleic acid therapeutics have tremendous potential for clinical utilities with some success of small interfering RNA/messenger RNA-based drugs and vaccines [1,2]. Despite this promise, their broader application is constrained by delivery challenges due to unfavorable pharmacokinetic properties, metabolic instability and poor tissue/cellular permeability of ‘naked’ nucleic acids [3,4]. Cyclic dinucleotides (CDNs) such as cyclic dimeric adenosine monophosphate and cyclic adenosine monophosphate-guanosine monophosphate, as potent natural agonists of the stimulator of interferon gene (STING), are particularly affected by these limitations in delivery [5–7]. While lipid nanoparticles (LNPs) have become a benchmark delivery platform for large RNA molecules, their conventional formulations, which rely on electrostatic compaction of polynucleotides under acidic conditions, are fundamentally ineffective for encapsulating small, highly hydrophilic and zwitterionic CDNs [6,8]. The distinct molecular structure of CDNs, featuring exposed positively charged nucleobases and a limited number of phosphate groups, restricts stable encapsulation via electrostatic interactions alone [9,10]. Although cationic liposomes have been used to deliver CDNs, the optimal encapsulation efficiency (EE) is about 21% [11], highlighting the critical need for rationally designed lipid systems tailored to the unique physicochemical properties of CDNs.

The amine oxide functional group, characterized by a zwitterionic structure with a directly connected N–O bond, has attracted growing interest in drug design and biomaterial science [12–14]. Notably, amine oxides can form strong intermolecular hydrogen bonds with nucleobases such as adenine and guanine, stabilizing single-stranded nucleotide structures without significant interaction with pentose sugars of nucleotides [15,16]. Physiologically stable amine oxide lipids (AOLs) are common degradants of tertiary amine-based ionizable lipids [17]. Typically, trimethylamine-*N*-oxide, a natural protective osmolyte solute in marine animals, can stabilize structures of proteins through increasing intermolecular interactions in solutions to counteract denaturing factors such as urea [18–21]. Moreover, ionizable amine oxides exhibit a useful charge-switching behavior: they remain zwitterionic at neutral pH but become positively charged under acidic conditions [22,23]. We thus hypothesized that synthetic lipids incorporating an amine oxide moiety could facilitate high-efficiency CDN encapsulation and delivery through hydrogen bonding and pH-dependent electrostatic interactions.

**Figure 1.**
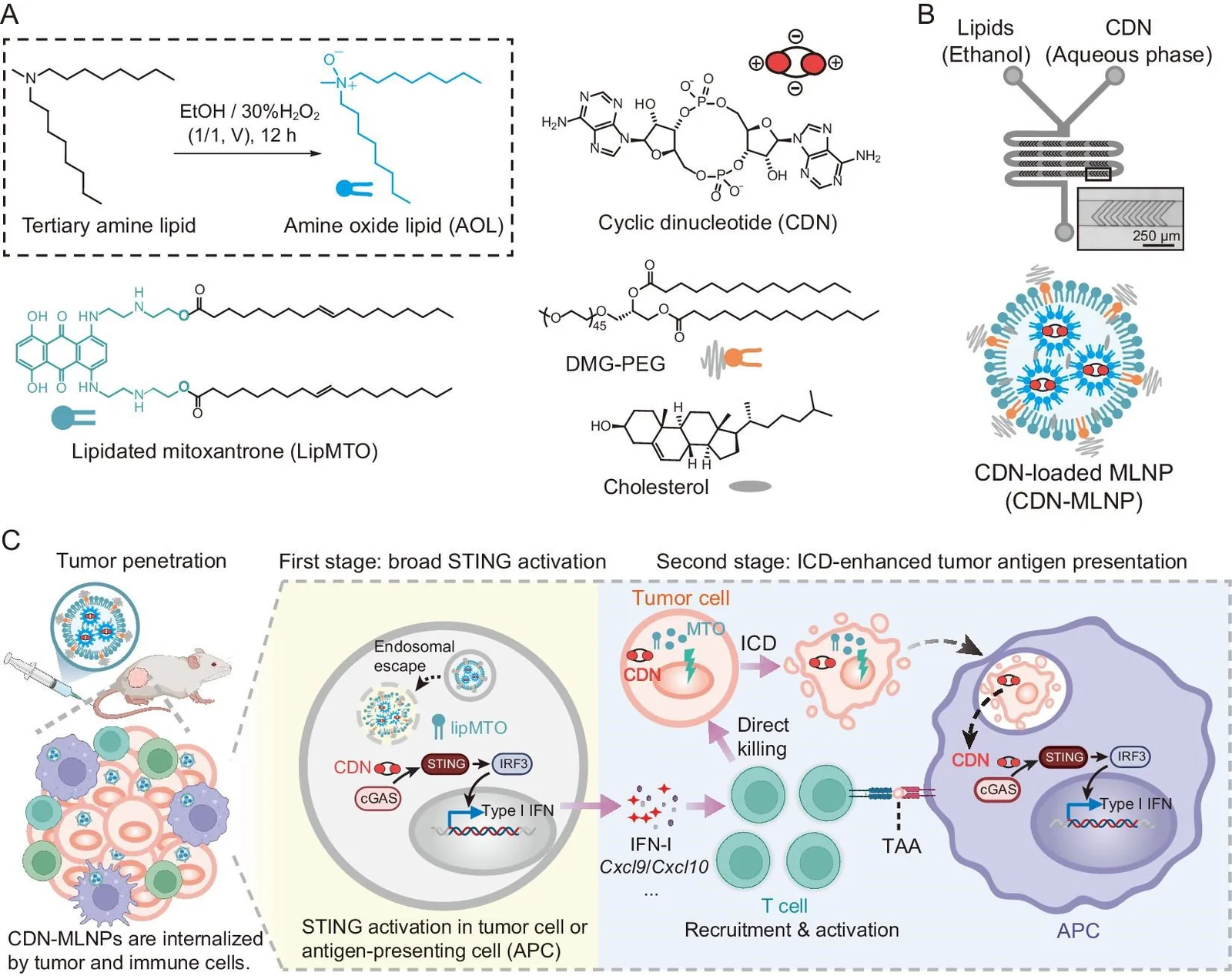
Schematic of formulation of amine oxide lipid (AOL)-incorporating CDN-MLNP and the mechanism of CDN-MLNP to improve tumor immunotherapy. (A) Chemical structures of four lipid components for preparing CDN-MLNP, including the AOL, lipMTO, 1,2-dimyristoyl-rac-glycero-3-methoxypolyethylene glycol-2000 (DMG-PEG), and cholesterol. (B) The microfluidic chip with herringbone textures was used to fabricate the CDN-MLNP. (C) The schematic shows the processes that CDN-MLNP accumulate in the tumor to trigger potent anti-tumor immunity through synergizing intratumoral STING activation and MTO-induced ICD. In the first stage, CDN-MLNP enables deep tumor penetration and delivers CDN to the cytoplasm. This broadly activate STING pathways in both tumor cells and APCs to induce the secretion of IFN-I and chemokines, resulting in the recruitment of T cells and creating a T cell-rich ‘hot’ microenvironment. In the second stage, the released MTO induces tumor ICD to facilitate the phagocytosis of tumor cells by APCs, priming the specific T-cell anti-tumor immunity through the ICD-enhanced tumor antigen presentation. Created with biorender.com.

In this study, we developed the LNP incorporating the AOL for systemic CDN delivery (Fig. 1A). The LNP composed of AOL, lipidated mitoxantrone (lipMTO), lipid-PEG and cholesterol were prepared using a microfluidics-based approach (Fig. 1B). We demonstrated that AOL not only enables stable and highly efficient CDN encapsulation via strengthened intermolecular interactions but also promotes deep tumor penetration and enhanced endo/lysosomal escape, leading to broad intratumoral STING activation within the tumor. In light of the growing importance of combination therapy in clinical oncology, the lipMTO was incorporated as both a structural lipid and a cytotoxic component to form an MTO-based LNP (MLNP) and meanwhile augment STING-driven immunotherapy (Fig. 1C). Specifically, the CDN-loaded MLNP (CDN-MLNP) first elicit broad and potent intratumoral STING activation, resulting in the recruitment of T cells to create a T cell-rich ‘hot’ microenvironment. In the second stage, the lipMTO induces immunogenic cell death (ICD) to facilitate the phagocytosis of tumor cells by antigen-presenting cells (APCs), which enhances tumor antigen presentation for priming specific T-cell antitumor immunity. The CDN-MLNP demonstrated significant antitumor efficacy across multiple challenging murine models, including poorly immunogenic 4T1 breast tumors, highly metastatic B16-F10 melanomas, and immunosuppressive orthotopic KPC pancreatic ductal adenocarcinoma (PDAC), underscoring the therapeutic potential.

## RESULTS

### The molecular interaction between amine oxide lipid and CDN

**Figure 2.**
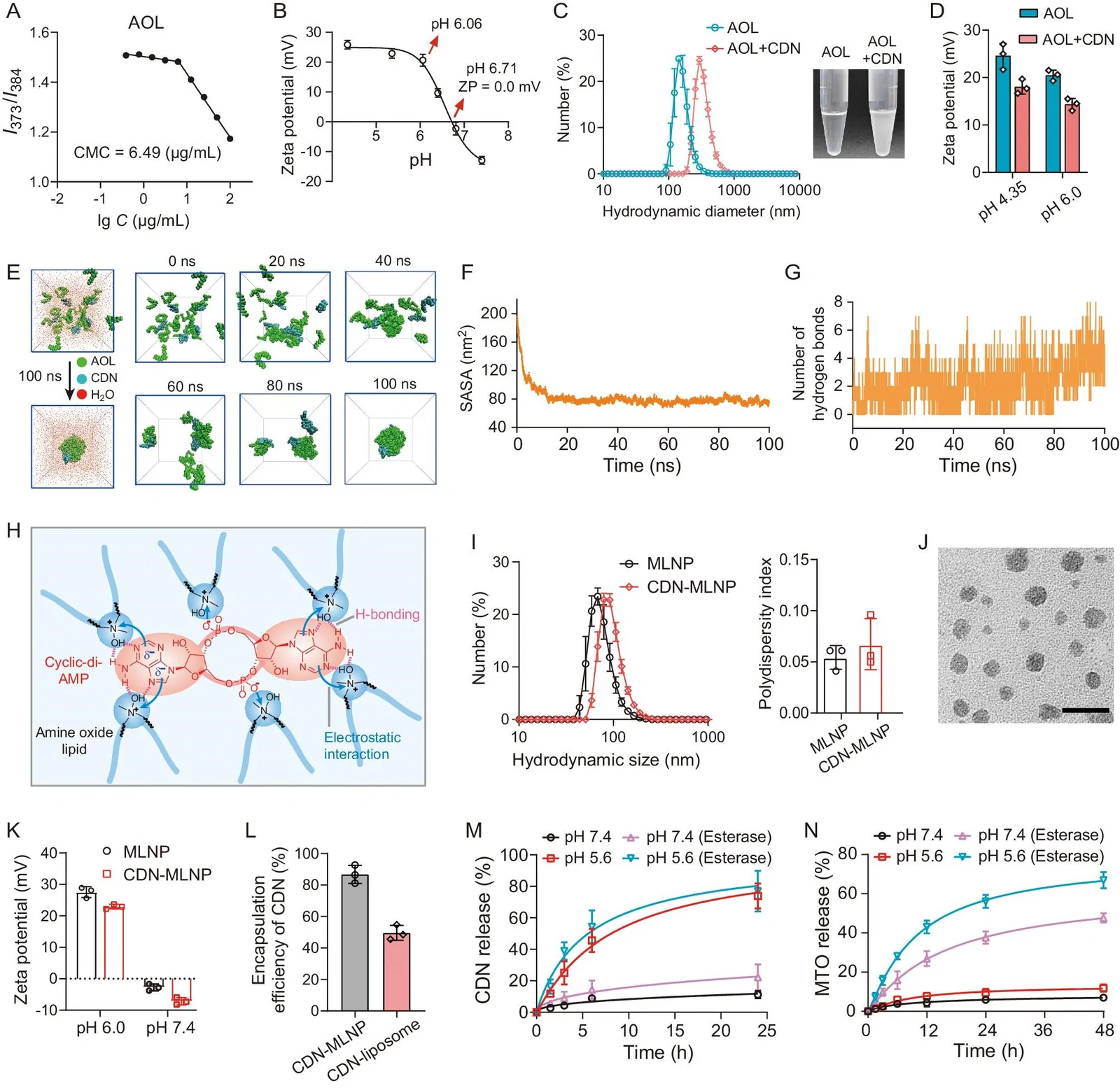
Molecular interaction between AOL and CDN and characterization of CDN-MLNP. (A) The determination of critical micelle concentrations (CMC) of the AOL. (B) The zeta-potentials of AOL micelle (0.5 mg/mL) in PBS buffer at different pH. Data were mean ± s.d. of replicates (*n* = 3). (C) The sizes of AOL micelle and AOL/CDN emulsion in the pH 6.0 acetic acid/sodium acetate buffer determined with dynamic light scattering (DLS). The right photo shows that adding CDN into the clear AOL solution enables the formation of white emulsion in seconds. (D) The Zeta potentials of AOL and AOL/CDN emulsions in acetic acid buffer at pH 4.35 or pH 6.0. (E) Molecular dynamics (MD) simulation snapshots of the self-assembly process of 30 AOL and 5 CDN molecules within a 6-nm water box over a 100 ns time scale. (F) Solvent-accessible surface area (SASA) as a function of time in the simulating system. (G) Variation of the number of intermolecular hydrogen bonds in the CDN/AOL system with simulation time. (H) The intermolecular interacting mode between AOL and CDN. (I) Particle sizes and polydispersity indexes of unloaded MLNP and CDN-MLNP measured by dynamic light scattering. (J) The TEM image of CDN-MLNP. Scale bar = 100 nm. (K) Zeta potentials of CDN-MLNP in PBS buffer with a pH of 7.4 or 6.0. (L) The encapsulation efficiency (EE) of CDN entrapped in CDN-MLNP and CDN-loaded liposomes measured by liquid chromatography tandem mass spectrometry (LC-MS). This liposome was fabricated by replacing AOL with DOTAP (a cationic lipid) while maintaining other components unchanged. The EE was obtained by calculating the percentage of CDN amount in the purified CDN-MLNP solution of the unpurified solution. (M) Release profiles of CDN from CDN-MLNP in PBS (pH 5.6 or 7.4) with or without esterase (50 U/mL). (N) The esterase-responsive release of MTO in PBS with a pH of 5.6 or 7.4 buffers through detecting the fluorescence of MTO.

We investigated the molecular interaction between AOL and CDN. AOL (*N*-methyl-*N*-octyloctan-amine oxide, MOAO) contains an amine oxide headgroup and two short alkyl tails and was synthesized by oxidizing the corresponding tertiary amine lipid (Fig. 1A and Fig. S1). AOL exhibits a critical micelle concentration (CMC) of 6.49 μg/mL in water (Fig. 2A), and its isoelectric point is at pH 6.71 as determined by zeta potential measurements (Fig. 2B). The AOL solution at pH 6.0 or pH 4.35 (acetate buffer) is clear, but when CDN solution was added, the mixture immediately formed a white emulsion, indicating co-assembly between AOL and CDN. AOL/CDN assemblies exhibit significantly larger size than AOL micelles alone (Fig. 2C and Fig. S2). In the pH 6.0 buffer, the zeta potential of the AOL/CDN assemblies decreased to +14.3 mV, compared to +20.5 mV of AOL micelles (Fig. 2D), suggesting electrostatic involvement in the co-assembly process. Molecular dynamics (MD) simulations revealed that 30 AOL and 5 CDN molecules co-assembled into a stable aggregate within a 6-nm water box over 100 ns (Fig. 2E). The solvent-accessible surface area decreased rapidly during the initial simulation phase and stabilized after 20 ns (Fig. 2F), indicating the formation of a compact nanostructure with reduced solvent exposure. The total interaction energy of −372.4 kJ/mol of solute molecules signifies strong net attraction within the system. The average electrostatic energy contributed with electrostatic and hydrogen bonding interactions is −112.4 kJ/mol. Van der Waals interactions (−260.0 kJ/mol) driven by hydrophobic aggregation of AOL molecules dominated in the assembly process (Fig. S3). The progressive increase in intermolecular hydrogen bonds further corroborates stable molecular association (Fig. 2G). We further investigated the interaction between AOL and CDN by ^1^H NMR spectra. Compared with the spectrum of pure CDN, the ^1^H NMR spectrum of a mixture of AOL/CDN shows that the protons (H1, H2 and H3) on CDN adenine groups shifted downfield, while the chemical shifts of protons on the middle 12-membered ring have no evident changes (Fig. S4). As demonstrated by previous studies that the oxygen of N-oxide could form strong hydrogen bonds with the amino groups of adenine or guanine of RNA, the hydrogen bonds between AOL and aminos of CDN caused effective molecular association, which results in the strengthened electron-withdrawing effect of positively charged AOL to *π* electronic clouds of adenine groups (Fig. 2H).

We then investigated the formation of CDN-lipid adducts in the AOL/CDN solution. It has been reported that tertiary AOLs can hydrolyze to generate aldehydes and secondary amines. These reactive aldehydes can then covalently bind to nucleobases, leading to the formation of lipid-nucleic acid adducts and a consequent loss of mRNA activity [17,24]. To test this, a phosphate-buffered saline (PBS) solution containing AOL (4 mM) and CDN (1 mM) was incubated at room temperature (∼20°C) for 48 h, followed by a 20-fold dilution with methanol for liquid chromatography tandem mass spectrometry (LC-MS) analysis. The results confirm the existence of both AOL hydrolysis products and CDN-lipid adducts (Fig. S5). However, the efficiencies of these reactions are low, with the proportions of AOL hydrolysis products (relative to total AOL) and adducts (relative to total CDN) being below 1% and 4%, respectively (Fig. S6). This systematic analysis thus demonstrates the chemical inertness of AOL under formulation and storage conditions.

### Design and characterization of CDN-MLNP

The CDN-MLNP were composed of four lipid components: AOL, lipMTO, 1,2-dimyristoyl-rac-glycero-3-methoxypolyethylene glycol-2000 (DMG-PEG), and cholesterol. While the amine oxide moiety has been widely employed in drug and biomaterial development, here we explored its utility in a synthetic ionizable lipid to overcome the delivery challenges of small nucleotides. The lipMTO was synthesized by conjugating mitoxantrone (MTO) with oleic acids to form an amphiphilic lipidoid (Fig. S7), exhibiting a CMC of 12.55 μg/mL (Fig. S8). As a lipidated prodrug of MTO, lipMTO functions not only as a cytotoxic component to enhance therapeutic efficacy, but also as a structural lipid. DMG-PEG and cholesterol were incorporated to improve the biocompatibility and stability of the MLNP. Lipid-prodrug nanoparticles based on synthetic lipidated drug hold considerable clinical translation potential due to their benefits in modulating drug release, reducing systemic toxicity and improving intracellular delivery [25–27].

The CDN-MLNP was prepared via microfluidic mixing of an ethanol phase containing the lipid components with an aqueous phase containing CDN. We employed pH 6.0 PBS as the aqueous phase, as this pH maintains AOL in a partially protonated state, which is sufficient to drive efficient encapsulation while remaining compatible with downstream biological applications. The feed molar percentages of AOL, lipMTO, cholesterol and DMG-PEG for preparing MLNP were set at 46.7%, 26.0%, 26.2% and 1.0%, respectively (Fig. S9A and B). The resulting CDN-MLNP displayed a uniform size of ∼94.3 nm and a low polydispersity index of ∼0.06 (Fig. 2I), and exhibited good serum stability (Fig. S10). Transmission electron microscopy (TEM) imaging revealed typical LNP morphology (Fig. 2J). The positively charged AOL rapidly forms inverted micelles to entrap negatively charged CDN via electrostatic and hydrogen bonding interactions, and then other lipid components assemble around the micelles, stabilizing the final LNP structure. Moreover, the smaller size of CDN-MLNP compared to AOL/CDN assemblies is attributed to the introduction of additional lipids and microfluidic fabrication [28–30]. The zeta potential of CDN-MLNP shifted from +22.9 mV at pH 6.0 to −7.14 mV at pH 7.4, reflecting pH-dependent protonation behavior (Fig. 2K). As quantified by LC-MS, the EE of CDN in AOL-incorporated MLNP reached 86.8 ± 5.76% and the loading capacity was 11.5% (Fig. 2L and Fig. S9C). For comparison, a cationic liposome (formulated with lipMTO, DOTAP, cholesterol and DMG-PEG) was fabricated by replacing AOL with the cationic lipid DOTAP (1,2-dioleoyl-3-trimethylammonium-propane) while keeping other components unchanged. The EE of CDN in the cationic liposome was only 49.6% (Fig. 2L and Fig. S9), confirming the critical role of AOL in driving CDN encapsulation.

We investigated, using LC-MS, the drug release kinetics of CDN in PBS with a pH of 5.6 that was close to the pH of late endosome [31]. More than 70% of CDN was released within 24 h, compared to only 11.2% at pH 7.4 condition (Fig. 2M). The addition of esterase (50 U/mL) resulted in only a marginal increase in CDN release under both pH conditions. This demonstrates both the stability of CDN encapsulation under physiological conditions and its responsive release in weakly acidic compartments. MTO release was assessed by detecting via its fluorescence. The esterase significantly accelerated MTO release (Fig. 2N). Even with the catalysis of esterase, MTO was released more slowly than CDN. The half-release time (*t*_1/2_) of MTO at pH 5.6 was 16.8 h, compared to 7.0 h for CDN. We propose that this differential release profile is conducive to a coordinated antitumor immune response. The rapid release of CDN facilitates early STING activation, initiating immune cell recruitment, while the delayed release of MTO enables subsequent induction of synthetic ICD. This sequence allows for the establishment of an inflammatory tumor microenvironment via STING signaling prior to the exposure of tumor-associated antigens through ICD, thereby potentially enhancing the priming of tumor-specific T cells and overall therapeutic efficacy.

### Endo/lysosomal escape and tumor accumulation/penetration of CDN-MLNP

**Figure 3.**
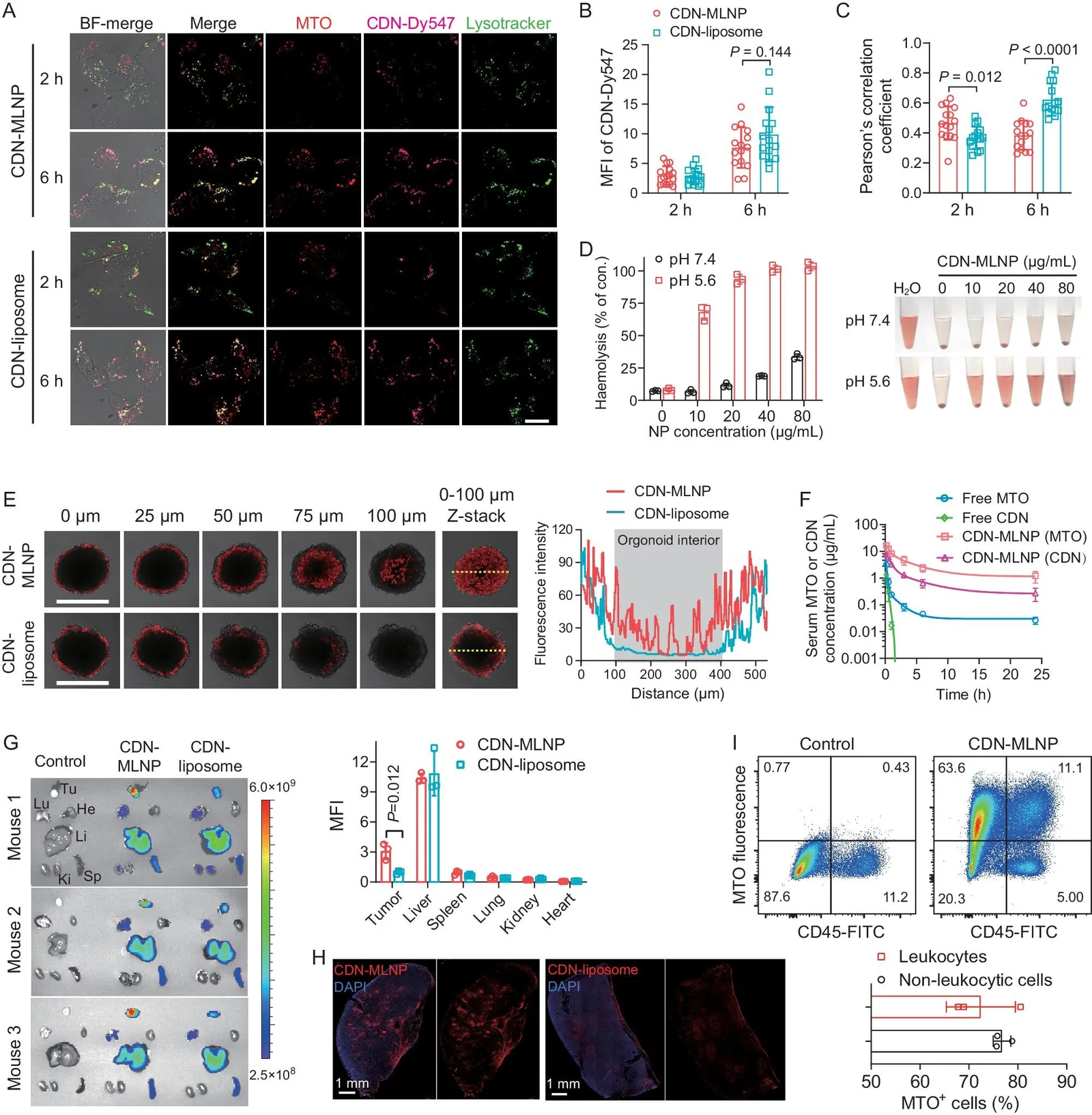
Cellular endo/lysosomal escape and tumor accumulation/penetration of CDN-MLNP. (A) Confocal images of 4T1 cells treated with CDG-Dy547-loaded CDN-MLNP or CDN-liposome for 2 or 6 h. MTO (red), CDG-Dy547 (magenta), and acidic compartments (LysoTracker-Green) are shown. Scale bar = 20 μm. (B) Quantification of CDG‑Dy547 mean fluorescence intensity (MFI) per cell (*n* = 15 cells). (C) The quantitative analysis of co-localization of CDN-Dy547 with endo/lysosomes labelled with LysoTracker Green (*n* = 15 cells). The Pearson’s correlation coefficients are close to 1 if they are highly co-localized. (D) pH-dependent hemolysis of CDN-MLNP. (E) The confocal images of the 4T1 tumor organoid at varied depths upon incubation with CDN-MLNP or CDN-liposome for 12 h. The corresponding quantification analysis shows the NP distribution at the border and interior of tumor organoid. (F) Pharmacokinetics of CDN and MTO in BALB/c mice injected (i.v.) with CDN+MTO mixture or CDN-MLNP. The curves were plotted by a two-phase exponential decay fit (*n* = 3 per group). (G) *Ex vivo* imaging of main organs and tumors from mice after a single i.v. administration of NPs into subcutaneous 4T1 tumor-bearing BALB/c nude mice (tumor volume, ∼100 mm^3^), and the related quantification of MFI or main organs (*n* = 3 mice per group). (H) 4T1-tumor-bearing mice were treated with CDN-MLNP or CDN-liposome, and tumors were excised 24 h later for histology. Shown are representative whole tumor cross sections from the treated mice. (I) The mice were injected with CDN-MLNP and their tumors were excised 24 h later, and the cellular internalization of CDN-MLNP by lymphocytes (CD45^+^ cells) and non-lymphocytic cells (CD45^−^ cells) in the tumors was analyzed by the flow cytometry (*n* = 3 animals per group).

We investigated whether CDN-MLNP containing AOL could enable effective tumor penetration and cargo release in the cytoplasm. 4T1 cells were treated with CDN-MLNP and stained endo/lysosomes with LysoTracker Green. CDN is prone to be degraded within the body and cells, in particular lysosomes. Confocal microscopic images show that CDN-MLNP and MLNP effectively enter cells and show good endo/lysosomal escape efficiencies, since the apparent signal separations between MTO and endo/lysosomes are shown in cells (Fig. S11). To investigate the intracellular fate of CDN, we prepared CDN-MLNP and CDN-liposome loaded with a fluorophore-labeled CDN (CDG-Dy547) for confocal imaging (Fig. 3A). Both of them exhibited time‑dependent cellular uptake (Fig. 3B). Co-localization analysis revealed that both formulations displayed low Pearson’s correlation coefficients (*R*) with LysoTracker at 2 h, indicating limited lysosomal entrapment. At 6 h, CDN-liposome showed increased co-localization (*R* = 0.62), whereas CDN-MLNP maintained a low level (*R* = 0.38), demonstrating more efficient lysosomal escape (Fig. 3C). The endo/lysosomal escape ability of CDN-MLNP might be ascribed to their membrane-disruptive activity after protonation; thus, a hemolysis test was performed to evaluate the membrane-disruptive activity. As expected, CDN-MLNP exhibited obviously higher hemolysis in pH 5.6 buffer than that of pH 7.4, validating their ability of membrane fusion and rupture (Fig. 3D). We then evaluated the tissue penetration of CDN-MLNP using 4T1 tumor organoids. Z‑stack images were acquired by confocal 3D scanning from the equatorial plane of the spheroid downward at depths of 0–100 μm (Fig. S12). Compared with CDN-liposomes, CDN-MLNP exhibit deeper penetration in 4T1 tumor organoids to deliver agents to the deep tissue, as the tumor organoid treated with CDN-MLNP has more fluorescent signals of MTO and significantly higher signals in the organoid core (Fig. 3E and Fig. S12).

To evaluate the *in vivo* pharmacokinetics (PK) of the formulations, BALB/c mice were intravenously injected with a CDN+MTO mixture or CDN-MLNP, and plasma concentrations of CDN and MTO were quantified at predetermined time points. Both CDN and MTO in the CDN-MLNP group exhibited prolonged circulation compared with the free CDN+MTO mixture (Fig. 3F and Fig. S13). CDN-MLNP markedly prolonged the terminal half-life (*t*_1/2_*β*) of CDN from 0.19 h (free mixture) to 3.01 h, accompanied by a substantial increase in systemic exposure, with the area under curve (AUC) increasing from 0.31 to 20.85 μg h/mL (Fig. S13). Free MTO was also rapidly cleared from circulation, whereas CDN delivered by CDN-MLNP exhibited significantly improved retention, as reflected by an increased AUC and prolonged elimination phase. Overall, these results demonstrate that CDN-MLNP enhances the pharmacokinetic profiles of both CDN and MTO, enabling prolonged circulation and increased systemic exposure, which may contribute *in vivo* therapeutic efficacy.

We investigated *in vivo* tumor accumulation and intratumoral distribution of CDN-MLNP in 4T1 tumor-bearing mice. At 24 h post-injection (50 μg MTO per mouse, intravenous (i.v.) injection), the fluorescence images of main organs and tumors of mice showed that CDN-MLNP mainly accumulated in tumors and livers (Fig. 3G). Although the tumor volumes were relatively small (∼100 mm^3^), around one-fourth fluorescent signals of LNPs were shown in tumors, and fluorescence signals per unit area of the tumor were higher than that of the liver, while the CDN-liposome had less accumulation in the tumor. The whole tumor sections also showed more fluorescent signals in CDN-LNP-treated group than that of CDN-liposomes (Fig. 3H). To unravel whether CDN-MLNP could extravasate and penetrate deeply into tumor tissues following tumor accumulation, the vascular endothelial cells were labeled by immunofluorescent staining of CD31 to visualize the tumor vasculature. The NP fluorescence signals were distributed throughout the tumor section and showed circumvascular distribution, suggestive of the deep tissue penetration (Fig. S14). The delivery carrier which is better able to penetrate beyond the tumor vasculature can expose the majority of cells in the tumor to the STING agonist, ensuring optimal activation of DCs for subsequent T-cell-mediated tumor immunity. We found that 72.4% of CD45^+^ leukocytes and 76.8% of CD45- non-leukocytic cells in tumors showed positive for NPs (Fig. 3I). Thus, CDN-MLNP can be internalized by most cells in the tumor tissue, with the potential to activate STING pathways of both tumor cells and immune cells. Altogether, the chemical properties of AOL—namely, that it is a zwitterion and transforms to a cationic lipid under acidic conditions, endow CDN-MLNP with significant advantages in cytosolic cargo delivery and tumor penetration, as confirmed by *in vitro* and *in vivo* investigations.

### Immunostimulatory potency of CDN-MLNP *in vitro*

**Figure 4.**
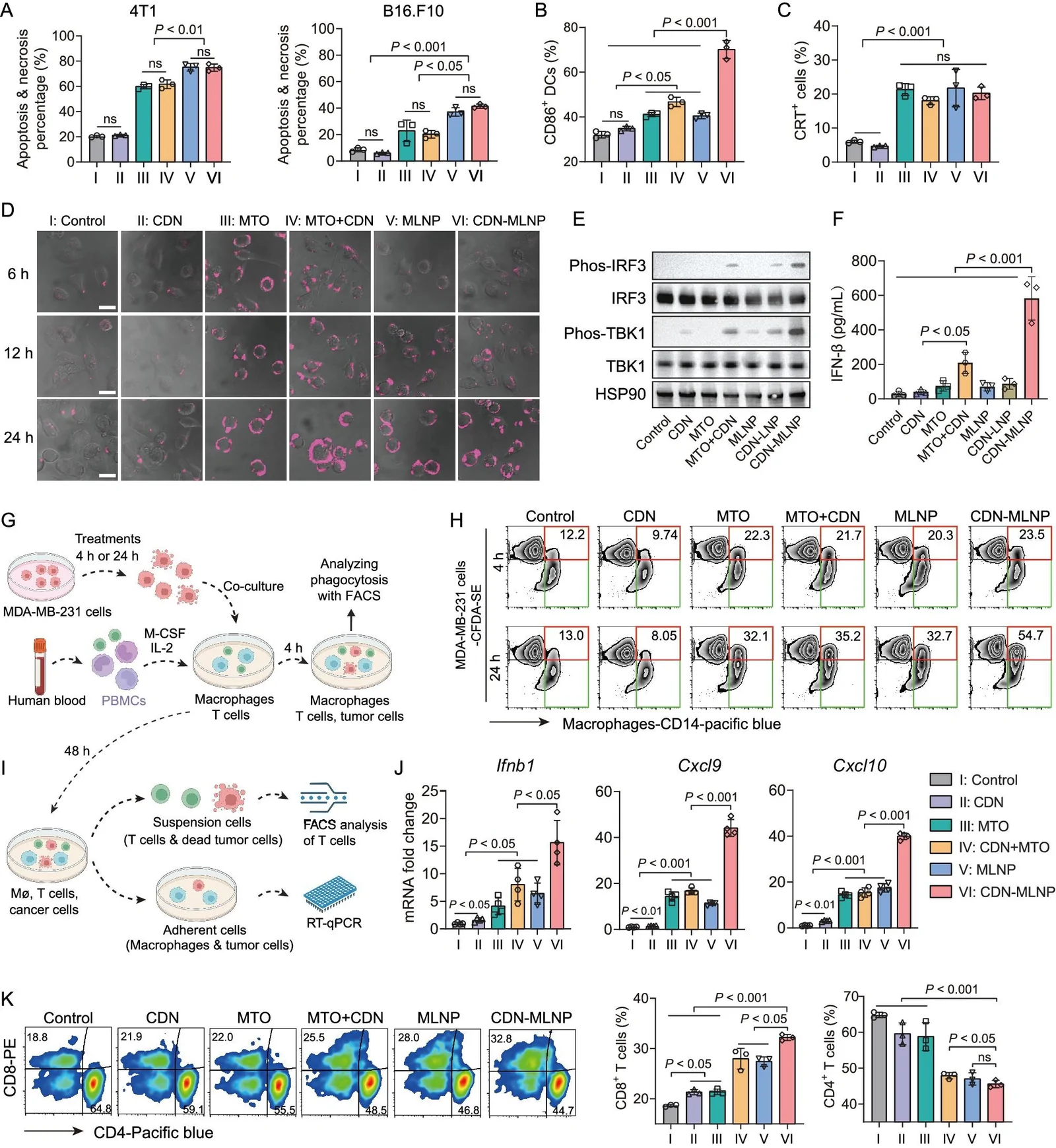
*In vitro* induction of immunogenic cell death and immune stimulatory capacity of CDN-MLNP. (A) The cell apoptosis/necrosis of 4T1 and B16.F10 cells were analyzed by flow cytometry using Annexin-PI staining after incubation with different formulations for 24 h. (B) Flow cytometry analysis of CD86-positive BMDC after stimulation with pretreated 4T1 cells with each formulation. (C) Flow cytometry analysis of calreticulin (CRT) levels on living 4T1 cells treated with each formulation for 24 h. (D) Confocal images show CRT exposure on the membrane of 4T1 cells treated with each agent for different time. Scale bar = 25 μm. (E and F) BMDMs from BALB/c mice were incubated with pretreated 4T1 cells with each formulation for 24 h, followed by western blotting of marker proteins in STING-IRF3 pathway (E) and ELISA analysis of supernatant IFN-*β* level (F). (G) The schematic of the experimental design for evaluating the phagocytosis of MDA-MB-231 cells by human macrophages. (H) Flow cytometry counter diagrams showing the phagocytosis of MDA-MB-231 cells by human macrophages. The phagocytosis was calculated as the percentage of double-positive macrophages among CD14-positive macrophages. (I) A schematic of the experimental design for evaluating the activation of STING pathway and anti-tumor T cell activation in co-culture systems of cancer cells, macrophages and PBMCs. (J) qPCR analysis of expressions of *Ifnb1, Cxcl9* and *Cxcl10* of adherent cells in the co-culture systems. *n* = 4 biologically independent culture wells. (K) Representative flow cytometric plots and the corresponding quantification of CD8^+^ and CD4^+^ T cells in the co-culture systems. All experiments were conducted with *n* = 3 biologically independent culture wells unless otherwise indicated. Data are represented as means ± s.d., and statistical significance was calculated by one-way ANOVA or Student’s *t* test with Tukey test. Panels (G) and (I) were created with BioRender.com.

We assessed the capacity of CDN-MLNP to induce cytotoxic effect and ICD, given that the incorporated MTO is an effective ICD inducer [32,33]. An ICD inducer can cause tumor cells to die as it activates antitumor immunity through promoting the maturation of innate immune cells and phagocytosis of tumor cells by APCs. For instance, calreticulin (CRT) exposed on the tumor cell membrane is a typical feature of ICD, as an ‘eat me’ signal to enhance antigen presentation [32–34]. Although STING agonists hold promise for cancer therapy, tumor resistance to STING monotherapy has emerged particularly in the treatment of immunosuppressive tumors like PDAC and triple-negative breast cancer (TNBC) [35–38]. These tumors are in the immune ‘cold’ state, characterized by the lack of initially infiltrating immune cells and tumor immune evasion through downregulating MHC-I and surface antigens [39–41]. Studies demonstrated that tumor-intrinsic STING activation could elevate the levels of IFN-I and chemokines like *Cxcl9*/*Cxcl10* to increase the recruitment of immune cells [35,42–47]. However, to elicit effective specific antitumor responses, tumor antigens must be taken up and presented by APCs. Once activated, CD8^+^ T cells can only recognize and kill tumor cells with exposed MHC-I-antigen complex [41,48]. CDN is not able to cause cytotoxicity to tumor cells. Thus, an ICD inducer may enhance STING-based immunotherapy through promoting antigen cross-presentation. The confocal images of cell live/dead staining showed that free MTO and MLNP exhibited dose-dependent cytotoxicity against 4T1 cells, while CDN alone had no cytotoxicity (Fig. S15). Flow cytometry results showed that MTO-related formulations induced significant apoptosis/necrosis in 4T1 and B16-F10 cells within 24 h, while CDN had no effect on the cells (Fig. 4A and Fig. S16).

We then investigated the immunostimulatory effect of CDN-MLNP using the co-culture system of TNBC cells and immune cells. The STING expression in four cancer cell lines (4T1, B16-F10, KPC, and MDA-MB-231) involved in this study was firstly confirmed using western-blot assays (Fig. S17). We treated 4T1 cells (a mouse TNBC cell line) with different formulations and then co-cultured them with bone marrow-derived dendritic cells (BMDCs) to analyze the maturation of DCs (Fig. S18), which was the first step for activation of innate immunity. The results showed that CDN-MLNP-treated tumor cells were much more effective to promote the maturation of BMDCs than other treatments (Fig. 4B and Fig. S18C). Free MTO-treated 4T1 cells induced the DC maturation to some extent, and the combination of MTO and CDN further enhanced this effect. However, free CDN-treated tumor cells had no effect on DC maturation, likely because free CDN alone internalized by tumor cells could not be transmitted to DCs. To assess ICD, we stained the membrane calreticulin (CRT), a marker translocated to the cell membrane during ICD. Flow cytometry revealed that MTO-related formulations significantly increased CRT expression compared to CDN-treated 4T1 cells (Fig. 4C and Fig. S19). Live-cell imaging showed that free MTO induced obvious CRT exposure on cell membrane within 6 h, while LPN and CDN-MLNP induced very limited CRT exposure at 6 h and a moderate CRT exposure level at 12 h. We observed comparable CRT expression on cell membrane with the free MTO-treated cells until 24 h, which was due to slower nanoparticle internalization and sustainable release of MTO parent drug (Fig. 4D).

To validate the activation of the STING pathway, we assessed key downstream signaling events in bone marrow-derived macrophages (BMDMs) following co-culture with treated 4T1 tumor cells. 4T1 cells were incubated with various formulations (PBS, CDN, MTO, MTO+CDN, MLNP, CDN-LNP and CDN-MLNP) for 24 h, followed by co-culture with BMDMs for an additional 24 h. CDN-LNP was formulated with AOL, neutral phospholipid DSPC (1,2-distearoyl-sn-glycero-3-phosphocholine), DMG-PEG and cholesterol. Western blot analysis revealed that CDN-MLNP markedly increased the phosphorylation levels of TBK1 and IRF3 in BMDMs, whereas total protein levels of TBK1 and IRF3 remained unchanged across groups (Fig. 4E and Fig. S20). Consistently, ELISA quantification showed that CDN-MLNP induced the highest secretion of IFN-*β* among all treatment groups (Fig. 4F). Of note, while CDN alone did not induce detectable STING activation, the MTO+CDN mixture led to moderate phosphorylation of TBK1 and IRF3, accompanied by elevated IFN‑*β* levels. This observation may be attributed to efficient phagocytosis of MTO-treated 4T1 cells by BMDMs, facilitating the transfer of CDN from tumor cells to macrophages.

To evaluate the translational relevance of the phagocytosis-dependent mechanism in a human context, we assessed phagocytosis of MDA-MB-231 cells (a human TNBC cell line) by macrophages derived from human peripheral blood mononuclear cells (PBMCs). Cancer cells were treated with different formulations for 4 or 24 h and then co-cultured with macrophages for another 4 h, and tumor-associated antigens (TAAs) were labeled with CFDA-SE (Fig. 4G). With 4-h pretreatment, there were no differences in phagocytosis among groups of MTO, MTO+CDN, unloaded MLNP and CDN-MLNP. Treatment of CDN-MLNP for 24 h significantly enhanced phagocytosis of cancer cells by macrophages compared to other groups (Fig. 4H). Consistently, confocal images and the corresponding quantification results also showed the more obvious phagocytosis of cancer cells by macrophages in CDN-MLNP groups, as the fluorescent signals of CFDA-SE-labeled TAAs were localized in more macrophages (Fig. S21). These results indicate that CDN-MLNP can be effectively internalized by cancer cells to induce their ICD and phagocytosis by macrophages, which further facilitates the phagocytic ability of the macrophages due to the existence of STING agonist.

The cancer cells treated with different agents for 24 h were used to co-culture with macrophages and other immune cells in PBMCs for 48 h, and then the suspended cells were analyzed to confirm T-cell activation and the adherent cells (mainly including macrophages and living cancer cells) were analyzed by real-time quantitative PCR (RT-qPCR) (Fig. 4I). CDN-MLNP-treated cancer cells upregulated the expression of *Ifnb1, Cxcl9*, and *Cxcl10* in adherent cells, indicating the STING pathway activation (Fig. 4J). Free MTO, MTO+CDN, and unloaded MLNP moderately upregulated these genes, while free CDN had minimal effects, as MTO treatment facilitated CDN reutilization by macrophages through enhanced phagocytosis. Compared to other groups, the percentage of CD8^+^ T cells in the co-culture system was increased while the ratio of CD4^+^ T cells was decreased in CDN-MLNP group (Fig. 4K and Fig. S22), because CDN-MLNP-treated cancer cells could effectively increase the number of matured APCs with antigen-presenting ability and these matured APCs further induced the activation and proliferation of CD8^+^ T cells through MHC-mediated antigen presenting and CD80/86 co-stimulatory signals.

These results demonstrate the ability of CDN-MLNP to induce ICD to facilitate the presentation of TAAs and CDN to APCs for the stimulation of interferon pathways and CD8^+^ T cell proliferation. In contrast, tumor cells treated with CDN alone minimally activated interferon pathways of macrophage, as macrophages poorly phagocytosed weakly immunogenic MDA-MB-231 cells, limiting their access to CDN and TAAs. These results highlight the importance of the co-delivery of MTO and CDN, which facilitates the transmission of CDN from tumor cells to APCs and compels tumor cells to be a depot of CDN. The chemical design of the CDN-MLNP, combining high-efficiency CDN encapsulation with controlled MTO release, is essential for enabling this strategy.

### CDN-MLNP sequentially induces immune cell recruitment and ICD within tumors

**Figure 5.**
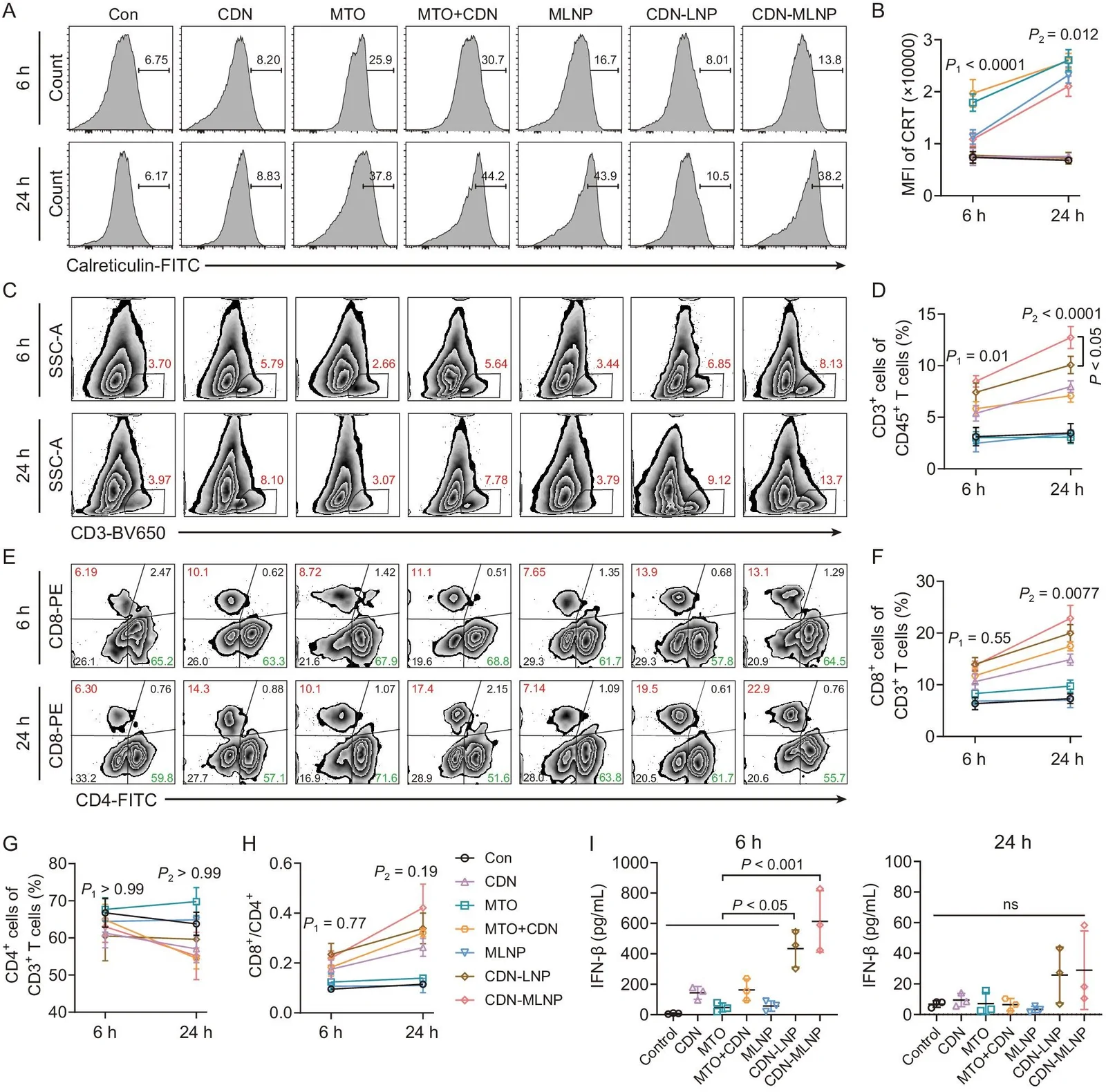
Time-dependent induction of ICD in tumor cells and T cell infiltration by CDN-MLNP. 4T1 tumor-bearing mice were intratumorally injected with different treatments (single dose), and the induction of ICD in tumor cells and T cell infiltration was assessed using flow cytometry at 6 and 24 h post-injection. (A and B) Representative flow cytometric plots (A) and quantification (B) of CRT-positive cells (gated from CD45^−^ cells) in the tumor. (C and D) Representative flow cytometric plots (C) and quantification (D) of tumor-infiltrating CD3^+^ cells. (E–G) Representative flow cytometry plots (E) and quantification (F and G) of the percentage of CD8^+^ (F) and CD4^+^ (G) T cells within the CD3^+^ population. (H) The ratio of CD8^+^ to CD4^+^ T cells in the tumor following treatment. (I) Serum IFN-*β* levels measured by ELISA at 6 and 24 h post-injection. *n* = 3 biologically independent samples. Data are represented as means ± s.d., and statistical significance was calculated by one-way ANOVA with Tukey test. *P*_1_ and *P*_2_ represent the significance test results for the comparison between MTO+CDN and CDN-MLNP groups at 6 h and 24 h, respectively.

To evaluate the ability of CDN-MLNP to induce ICD and promote immune cell infiltration, 4T1 tumor-bearing mice were intratumorally injected with different formulations (5 μg CDN per mouse). Tumors were collected at 6 and 24 h post-injection and analyzed by flow cytometry (Fig. S23). Compared with the free MTO+CDN group, the expression level of CRT in CDN-MLNP-treated tumor decreased to 0.55-fold at 6 h post-injection but increased to 0.81-fold at 24 h post-injection, indicating a delayed cytotoxic effect of lipMTO due to its controlled release kinetics (Fig. 5A and B). Tumors treated with CDN-LNP or CDN-MLNP exhibited more CD3^+^ immune cell infiltration at both time points compared with all other groups. At 6 h, CDN-MLNP increased the proportion of CD3^+^ cells by 2.72-, 1.58-, 1.46- and 1.14-fold compared to control, free CDN, MTO+CDN and CDN-LNP groups, respectively. By 24 h, these increases further rose to 3.67-, 4.14-, 1.80- and 1.27-fold respectively, demonstrating enhanced and sustained immune cell recruitment (Fig. 5C and D). This effect can be attributed to the efficient delivery of CDN by CDN-MLNP to both tumor and immune cells, thereby inducing chemokine secretion and promoting immune cell recruitment, whereas free CDN shows limited activity due to its poor stability and membrane permeability. Further analysis revealed that the percentage of CD8^+^ T cells and the CD8^+^/CD4^+^ T cell ratio in tumors treated with CDN-MLNP were higher than those in other treatment groups at 24 h post-injection (Fig. 5E–H). Moreover, intratumoral injection of CDN-LNP or CDN-MLNP resulted in significantly enhanced systemic immune activation, as indicated by elevated serum IFN-*β* levels compared with free CDN or the CDN+MTO admixture (Fig. 5I).

Together, these results demonstrate that CDN-MLNP induces rapid and effective STING activation and immune cell infiltration, particularly of T cells, while initially eliciting a relatively low level of ICD in tumor cells. This finding suggests that immune cell recruitment precedes ICD. This ‘immune warm-up’ driven by broad STING activation within the tumor microenvironment is critical for maximizing therapeutic efficacy. The recruited T cells are subsequently primed for activation by tumor-associated antigens (TAAs) exposed during ICD, resulting in a synergistic antitumor response. By integrating the *in vivo* and *in vitro* findings, we further elucidated the immunostimulatory mechanism of CDN-MLNP (Fig. 1C). (1) Initiation stage: the enhanced tumor penetration and rapid CDN release from CDN-MLNP activate STING signaling in both tumor and immune cells, creating a T cell-inflamed microenvironment. (2) Effector stage: MTO induces ICD, facilitating the phagocytosis of tumor cells by APCs and subsequent T cell activation. This process also enables the transfer of CDN from tumor cells to APCs, promoting its reutilization. (3) Amplification stage: cytotoxic CD8^+^ T cell-mediated killing of tumor cells further amplifies the immune response by releasing additional TAAs, thereby sustaining antitumor immunity. CDN-MLNP thus serves as a potent initiator of this self-amplifying immune cascade, leveraging their unique chemical design to coordinate STING agonist and tumor antigens.

### Therapeutic efficacy and innate immune response in 4T1 tumor-bearing mice

**Figure 6.**
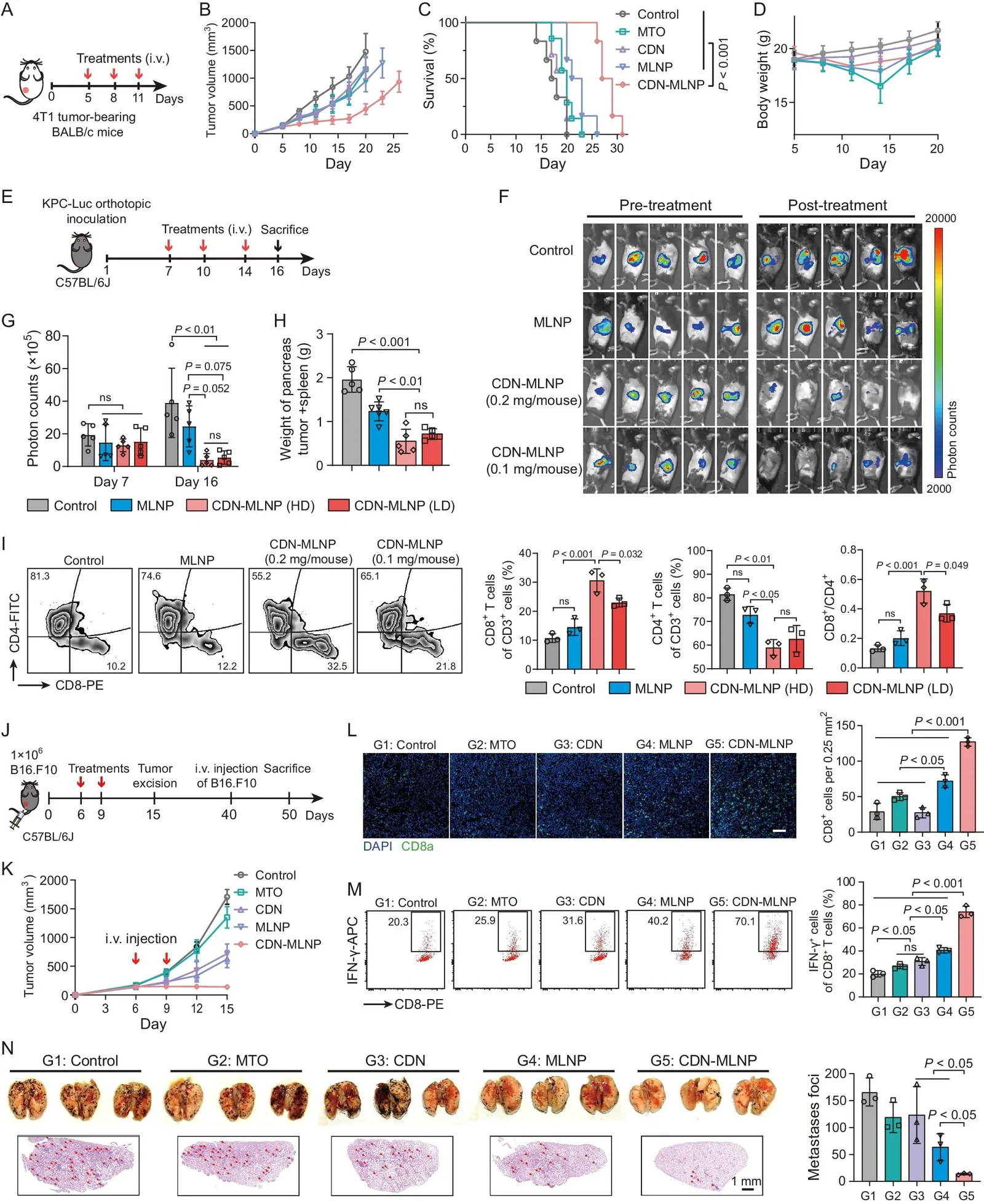
CDN-MLNP shows therapeutic efficacy and elicits effective anti-tumor immune response in mouse models of 4T1 breast cancer, orthotopic KPC pancreatic ductal adenocarcinoma (PDAC) and B16-F10 melanoma. (A) BALB/c mice were inoculated with 4T1 tumor cells and treated on days 5, 8, and 11 with i.v. administration of PBS (*n* = 6), MTO (50 μg per mouse, *n* = 7), CDN (20 μg per mouse, *n* = 7), unloaded MLNP (0.2 mg per mouse, *n* = 6), or CDN-MLNP (0.162 mg per mouse, *n* = 6). (B and C) Tumor growth curves (B) and overall survival (C) of 4T1 tumor-bearing mice. Survival data were analyzed using two-sided log-rank (Mantel-Cox) tests. (D) Body weight changes of 4T1 tumor-bearing mice following treatment with different agents. (E) The treatment scheme for mice with established pancreatic tumor by orthotopic inoculation of luciferase-expressed KPC (KPC-Luc) cells. (F) Bioluminescence imaging of mice bearing KPC (luciferase-expressed) orthotopic tumors at the start and end of treatments of PBS (*n* = 5), MLNP (0.2 mg per mouse, *n* = 6) and CDN-MLNP (0.2 or 0.1 mg per mouse, *n* = 5). (G) The quantification result of bioluminescence intensity of the mice tumors. (H) The weights of isolated pancreatic tumors along with spleens at the end of treatments. (I) Representative flow cytometry plots and quantification results showing the percentages of CD8^+^ and CD4^+^ T in CD45^+^CD3^+^ cells and ratio of CD8^+^ to CD4^+^ T cells in pancreatic tumors following the treatments. (J) Schematic of B16-F10 tumor inoculation and treatment schedule. (K) Tumor growth in B16-F10 melanoma-bearing mice (*n* = 5 mice per group) receiving two doses (i.v.). (L) Immunofluorescent staining of CD8a-positive T cells of tumor sections. The corresponding quantitative result shows the number of CD8a-positive cells per 0.25 mm^2^. Scale bar: 100 μm. (M) Representative flow cytometric analysis images (left) and the relative quantifications (right) of IFN-*γ*^+^CD8^+^ T cells in the B16-F10 tumors 6 days after the second treatment. (N) The mice were intravenously injected with B16-F10 cells on day 40 and sacrificed on day 50. The lungs isolated from the mice and related H&E staining specimens were displayed. The densely growing cell colonies are identified as tumor tissue on the lung specimens. The corresponding quantitative result shows the number of metastatic foci on the lung surface (front and back). Data are represented as means ± s.d., and statistical significance was calculated by one-way ANOVA with Tukey test.

We evaluated the therapeutic efficacy of CDN-MLNP in 4T1 tumor-bearing mice (Fig. 6A). Three doses of CDN-MLNP (equivalent to 20.0 μg CDN, 32.8 μg MTO, and 0.162 mg CDN-MLNP per mouse) significantly inhibited tumor growth, while free CDN (20 μg per mouse), MTO (50 μg per mouse), or unloaded MLNP (0.2 mg per mouse) showed very limited antitumor effects (Fig. 6B and Fig. S24). CDN-MLNP significantly extended survival compared to other treatments (two-sided log-rank test, Fig. 6C). We observed an obvious decrease in body weight of free MTO-treated mice during the treatment, while CDN-MLNP caused a smaller effect on body weight of mice (Fig. 6D).

To assess immune activation, another batch of 4T1 tumor-bearing mice were intratumorally or intravenously injected with different agents and the tumors were harvested for qPCR analysis at 12 h post-injection. Intratumorally injected CDN-MLNP induced the highest level of expressions of *Ifnb1* and *Cxcl10*. The intratumorally injected free MTO and CDN also elevated expressions of the two genes to some extent, but intravenous injections of both had no significant effect. By contrast, intravenous injection of CDN-MLNP up-regulated the expression of *Ifnb1* and *Cxcl10* to a great extent, which is attributed to the targeted delivery of CDN-MLNP to the tumor (Fig. S25A). These results demonstrated the effective activation of STING pathway within the tumor to trigger the secretion of IFN-I and chemokines that conferred immune cell recruitment and a ‘hot’ microenvironment.

To further evaluate the effect of CDN-MLNP on innate immune activation and biosafety, another batch of 4T1 tumor-bearing mice were intravenously administered with different formulations, and mice were sacrificed 6 h after the third dose to capture early innate immune responses and acute toxicity (Fig. S25B). CDN-MLNP showed the greatest efficacy in inhibiting the tumor growth and CDN-LNP was also effective (Fig. S25C). The ELISA results for serum cytokine levels showed that CDN-LNP and CDN-MLNP significantly elevated serum levels of IFN-*β*, TNF-*α* and IL-6 compared to other treatments, and CDN-MLNP induced the highest levels among all three cytokines (Fig. S25E). Intratumoral macrophages (CD45^+^F4/80^+^) and DCs (CD45^+^F4/80⁻CD11c^+^) were analyzed by flow cytometry for CD86 expression (Fig. S26). Both CDN-MLNP and CDN-LNP upregulated CD86 expression on intratumoral macrophages and dendritic cells, with CDN-MLNP showing a slightly higher increase over CDN-LNP (Fig. S25F and G). These results demonstrate that CDN-MLNP effectively activates innate immune cells and promotes pro-inflammatory cytokine production in the tumor microenvironment.

We performed the biosafety assessment using the same animal cohort. Complete blood count results showed free MTO and the MTO+CDN mixture caused a marked decrease in WBC and lymphocyte counts. In contrast, CDN-MLNP and CDN-LNP significantly elevated WBC and lymphocyte counts, suggesting effective immune cell mobilization. RBC counts remained unchanged across all groups, and platelet counts showed a mild decrease in the MTO and MTO+CDN groups (Fig. S27A). For the serum biochemistry analysis, CDN-MLNP induced only a mild, nonsignificant increase in ALT and ALP, while free MTO and MTO+CDN treatment resulted in significantly elevated levels exceeding the normal reference range. CDN-MLNP caused no significant changes of CR and BUN levels while slightly elevated levels in the MTO and MTO+CDN groups (Fig. S27B). Hematoxylin and eosin (H&E) staining of liver and kidney sections showed well-preserved physiological architecture across all groups, with no obvious pathological abnormalities such as necrosis and inflammatory infiltration (Fig. S27C). Although H&E staining of spleen sections showed no obvious necrosis or hemorrhage in all groups, focal regions with reduced cellular density and mild architectural alterations were observed in the free MTO-treated group, suggesting localized tissue changes. An increased density of basophilic cells was observed in the red pulp regions of the spleen in the CDN-LNP and CDN-MLNP groups. These cells, characterized by densely stained nuclei, may represent increased immune cell presence. In addition, AOL exhibited no cytotoxicity toward human umbilical vein endothelial cells, even at a concentration of 100 μg/mL (Fig. S28). These data demonstrate that CDN-MLNP treatment does not induce significant hematological toxicity, hepatotoxicity or nephrotoxicity, whereas free MTO treatments impact hematopoiesis and liver function. The observed increases in WBC and lymphocytes, as well as basophilic cell infiltration in the splenic red pulp, reflect the desired immunostimulatory effect of CDN-MLNP rather than toxicity.

### CDN-MLNP induces potent antitumor effect and adaptive immune response in mice

We next evaluated the efficacy of CDN-MLNP for treating orthotopic KPC PDAC and melanoma. To construct an orthotopic KPC PDAC model, C57BL/6 mice were orthotopically transplanted with luciferase-engineered KPC PDAC cells that derived from a KPC (*Kras^LSL-G12D/+^; Trp53^flox/flox^; Pdx1-cre*) genetically engineered mouse (Fig. 6E). The KPC PDAC tumor is characterized by a hallmark dense stroma, poor vascularization and high-level immunosuppression, which impedes effective drug delivery, drives chemoresistance and blocks immune cell activation and infiltration [36]. The results of *in vivo* bioluminescence imaging of mice show the effective inhibition of tumors in the treatment groups (Fig. 6F and G). After the treatment, we harvested the pancreatic tumors along with spleens and pancreas and weighed them. Compared with the unloaded MLNP (0.2 mg per mice) and control groups, treatments of CDN-MLNP at both doses (0.1 and 0.2 mg per mouse) led to decreased tumor weight, and there was no difference between the high-dose and low-lose treatments (Fig. 6H). Compared with the control group, CDN-MLNP at the doses of 0.2 and 0.1 mg per mouse increased the percentages of CD8^+^ T cells (gated from CD3^+^) by 19.9 and 12.2, respectively, and elevated the ratios of CD8^+^ T cells to CD4^+^ T cells, indicating the activation of T cells for inhibiting the tumor (Fig. 6I and Fig. S29). There was no difference in antitumor effect and T cell activation between the high and low dose of CDN-MLNP, confirming the role of CDN-MLNP as the initiator to trigger the antitumor immunity.

We constructed the B16-F10 melanoma-bearing mice as a highly metastatic tumor model (Fig. 6J). Systemic administration of CDN-MLNP (equivalent to 0.167 mg per mouse, twice) caused significant tumor regression, outperforming free CDN, MTO, and MLNP (Fig. 6K and Fig. S30). Free CDN at the dose of 20 μg per mouse could inhibit the tumor growth slightly, while free CDN at the dose of 0.5 μg per mouse had no effect on the growth of B16-F10 tumors as reported in the prior study. After 6 days of the second injection, tumors were resected for analysis and the mice wounds were covered with gauzes. Immunofluorescent staining revealed increased CD8^+^ T cell infiltration in the tumor of CDN-LNP-treated mice (Fig. 6L). Moreover, a higher proportion of CD8^+^ T cells were IFN-γ positive, indicating effective antitumor T cell activation (Fig. 6M and Fig. S31). To evaluate immune memory, we intravenously injected B16-F10 cells 25 days post-tumor excision. CDN-MLNP-treated mice exhibited the fewest lung metastatic foci, as confirmed by lung photos and H&E staining (Fig. 6N). This antimetastatic effect correlated with primary tumor regression. Additionally, spleens from CDN-MLNP-treated mice showed an increased percentage of CD8^+^ T cells and elevated ratio of CD8^+^ T cells to CD4^+^ T cells (Fig. S32). The spleen is the main site in which the tumor-specific CD8^+^ T cells after the immunotherapy, and these T cells play an important role in the long-term immune surveillance of cancer cells [49]. These results demonstrate that CDN-MLNP synergize STING activation and ICD induction to prime potent antitumor immunity, conferring long-term control of metastases and immune memory.

## CONCLUSION

In this study, we developed LNPs incorporating a custom AOL for efficient encapsulation and delivery of CDN-based STING agonists and a mitoxantrone-based lipidoid (lipMTO) for improving STING-based immunotherapy. The unique chemical properties of the ionizable AOL enable strong interactions with CDNs through both hydrogen bonding and electrostatic forces, driving co-assembly and resulting in an unprecedented EE of 86.8% in the CDN-MLNP. The incorporation of AOLs expands the application of lipid-based nanoparticles in effectively delivering hydrophilic small molecules via enhanced carrier-cargo interactions. The microfluidic fabrication method further ensures reproducible and scalable nanoparticle fabrication [29,50,51]. Unlike conventional tertiary amine-based ionizable lipids, AOL exhibits a zwitterionic character at neutral pH, contributing to improved biocompatibility and delivery performance. The resulting CDN-MLNP demonstrate robust tumor penetration and efficient cytosolic cargo release, enabling broad and potent STING activation across the tumor microenvironment. To amplify antitumor immunity, the chemotherapeutic prodrug lipMTO was incorporated to induce ICD. This design enables a spatiotemporally coordinated process: initial STING activation recruits immune cells to establish a T cell-inflamed ‘hot’ microenvironment, followed by MTO-triggered ICD that exposes tumor antigens. While tumor cells internalize a proportion of STING-activating LNPs, emerging evidence highlights the pivotal role of tumor cell-intrinsic STING pathway in suppressing metastases, elevating IFN-I levels, and recruiting T cells for immune surveillance [35,42–45]. The CDN-MLNP induces effective antitumor immune responses in multiple aggressive murine models (4T1 breast cancer, B16-F10 melanoma, orthotopic KPC PDAC) and in co-culture system of human TNBC cells and peripheral blood mononuclear cells. These results validate the potential of this platform for cancer immunotherapy, leveraging innovative chemical designs of carrier components to achieve high-efficiency delivery of cyclic dinucleotides.

## Supplementary Material

nwag340_Supplemental_File
