# Supplementary material for: Amine oxide lipid nanoparticles for delivery of cyclic dinucleotides
Source: Natl Sci Rev. 2026 Jun 8;13(16):nwag340. doi: 10.1093/nsr/nwag340 (PMC13532573; doi:10.1093/nsr/nwag340)
Supplement: Supplementary file 1 [file nwag340_supplemental_file.pdf]

Supplementary Information for

Amine oxide lipid nanoparticles for delivery of cyclic dinucleotides

Yao Chen[1,2], Yanfeng Yang[1], Feng Xiao[1], Qian Wang[1], Shengbo Chen[1], Jia'an Li[1], Saijie Wang[1], Yunhan Zhang[1], Jun Wu[2,*], and Xingyu Jiang[1,*]

[1]Shenzhen Key Laboratory of Smart Healthcare Engineering, Guangdong Provincial Key Laboratory of Advanced Biomaterials, Department of Biomedical Engineering, Southern University of Science and Technology, Nanshan District, ShenZhen, Guangdong 518055, P. R. China;

[2]Bioscience and Biomedical Engineering Thrust, Hong Kong University of Science and Technology (Guangzhou), Nansha, Guangzhou 511458, P. R. China

*Corresponding authors. E-mails: jiang@sustech.edu.cn; junwuhkust@ust.hk

Materials and methods

Materials: 1,2-dioleoyl-3-trimethylammonium-propane (DOTAP), cholesterol, 1,2-dimyristoyl-rac-glycero-3-methoxypolyethylene glycol-2000 (DMG-PEG2000) and cholesterol were from Avanti (USA). Cyclic-di-AMP disodium and DiI cell membrane dye were from MedChemExpress. N-methyl-N-octyoctan-amine (MOA) and ethanol were from Shanghai Aladdin Biochemical Technology Co., Ltd (Shanghai, China). Mitoxantrone (MTO), Di-tert-butyl decarbonate ($(Boc)_2O$), dichloromethane (DCM), trifluoroacetic acid (TFA) were from Shanghai Macklin Biochemical Technology Co., Ltd (Shanghai, China). Oleoyl chloride was from Beijing Vokai Biotechnology Co., Ltd (Beijing, China). LysotrackerGreen were from Life Technologies (Thermo Fisher Scientific). Antibodies for flow cytometry analysis including FITC anti-mouse CD45 (103107), PE anti-mouse CD45 (103105), APC anti-mouse CD3 (100235), PE anti-mouse CD8a (100707), FITC anti-mouse CD4 (100405), APC anti-mouse CD11c (117309), FITC anti-mouse CD86 (105005), Pacific Blue™ anti-human CD14 (325615), FITC anti-human CD3 (300306), PE anti-human CD8a (300907), and Pacific Blue™ anti-human CD4 (317423) were purchased from BioLegend, Inc. Antibodies for flow cytometry analysis including BV650 anti-mouse CD3 (416-0032-80), BV786 anti-mouse CD45 (417-0451-80) and BV650 anti-mouse F4/80

(416-4801-80) were from Thermofisher. Calreticulin (D3E6) XP® Rabbit mAb (Alexa Fluor® 488 Conjugate) (62304S) were from Cell Signaling Technology. Anti-CD31 antibody (ab281583) were from Abcam. Recombinant murine GM-CSF (315-03-5), recombinant murine IL-4 (214-14-5), recombinant human M-CSF (300-25-2) and recombinant human IL-2 (200-02-10) were obtained from PeproTech. Primers for RT-qPCR were synthesized by RuiBiotech Ltd (Beijing, China) with sequences in Table S1.

Cell lines and animals

4T1, B16F10 and MDA-MB-231 cells were sourced from American Type Culture Collection. 4T1 and MDA-MB-231 cells were cultured in Dulbecco's modified Eagle medium (11965092, Gibco), and B16-F10 cells were grown in Roswell Parks Memorial Institute 1640 (RPMI-1640) medium (11875119, Gibco) containing 10% fetal bovine serum (FBS) and 1× penicillin/streptomycin (PS, 15070063, Gibco) at 37 °C in 5% CO_2. Luciferase-engineered KPC PDAC cell line that derived from a KPC ($Kras^{LSL-G12D/+}$; $Trp53^{flox/flox}$; Pdx1-cre) genetically engineered mouse was from Shanghai Model Organisms Center, and the KPC cells were cultured in Dulbecco's modified Eagle medium (11965092, Gibco) containing 10% FBS) and 1× penicillin/streptomycin. All animal protocols were approved by the Animal Ethics Committee of Southern University of Science and Technology (LLBH-202004080001). Female C57BL/6J and BALB/c mice (4 weeks) were purchased from Beijing Vital River Laboratory Animal Technology Co., Ltd (https://vitalriver.biomart.cn/).

Synthesis of AOL and lipMTO

The amine oxide lipid, N-methyl-N-octyloctan-amine oxide (MOAO), was synthesized by oxidizing N-methyl-N-octyloctan-amine (MOA) with hydrogen peroxide (Supplementary Figure 1). 500 mg of MOA was dissolved in 5 mL of ethanol, and added 5 mL 30% H_2O_2 to make the clear solution become an emulsion. After stirring for 12 h at room temperature, the solution become clear again. Then the ethanol was removed using a rotary evaporator (RV 10 digital, IKA®) under the vacuum. To remove the H_2O_2, the solution was dialyzed (3500 Da molecular weight cut-off) in the water since MOAO formed large-sized micelles in water. Then the water was removed using a rotary evaporator to obtain an oily liquid. The ^1H NMR spectra were acquired using D_2O containing 1% CD_3COOD as the solvent on a 400 MHz spectrometer (AVANCE III 400M, Bruker, Switzerland) and analyzed by MestReNova (v. 9.0.1).

We conjugated MTO with oleoyl chloride to synthesize the lipMTO. 500 mg MTO (1.125 mmol) and 13 mg 4-Dimethylaminopyridine (DMAP, 0.1125 mmol) were dissolved in N,N-Dimethylformamide (DMF). The

reaction was stirred at room temperature for 4 h under a nitrogen atmosphere. The above reaction flask was cooled down on ice and oleoyl chloride (677 mg, 2.25 mmol) was added. After stirring for 1 h at 0 °C, 30 mL methanol was added to the reaction to quench this reaction. The solvent was removed using a rotary evaporator (RV 10 digital, IKA®) under the vacuum. The reaction flask was removed from the rotary evaporator and 50 mL of dichloromethane (DCM)/trifluoroacetic acid (TFA) (3/1, v/v) was added, and the reaction was continued at room temperature for 4 h. The solvent was removed using the rotary evaporator. The obtained crude product was purified by silica gel chromatography with DCM/methanol as the eluting solvents, followed by removing the solvent with a using rotary evaporator. The cut fractions were detected by thin-layer chromatography (TLC, Silica gel) on glass-packed precoated silica gel plates and visualized with a UV lamp. Dark blue solid with 41% yield was obtained. The ^{1}H NMR spectra were acquired using DMSO-d6 solvent with TMS (0 ppm) on the 400 MHz spectrometer. The high-resolution mass spectra of MOAO and lipoMTO were generated using a liquid chromatography with tandem mass spectrometry (LC-MS, Q-Exactive™, ThermoFisher Scientific), in which the samples did not go through the chromatographic column.

Molecular dynamics (MD) simulation

A 6 nm × 6 nm × 6 nm simulation box was constructed and randomly populated with 30 MOAO molecules and 5 CDN molecules to form the simulation system. Molecular dynamics simulations were performed using GROMACS 2022.2 under periodic boundary conditions at constant temperature and pressure. The GAFF all-atom force field and TIP3P water model were employed. During simulations, all involved hydrogen bonds were constrained using the LINCS algorithm with a 2-fs integration time step. Electrostatic interactions were calculated using the Particle-Mesh Ewald (PME) method, while non-bonded interactions utilized a 10-Å cutoff with neighbor list updates every 10 steps. Temperature was maintained at 298.15 K using the V-rescale thermostat, and pressure was controlled at 1 bar with the Parrinello-Rahman barostat. Systems were first energy-minimized via steepest descent to eliminate atomic clashes, followed by 100-ps NVT equilibration at 298.15 K. Production MD simulations were then conducted for 100 ns per system with trajectory snapshots saved every 10 ps. Conformation analysis was performed using GROMACS built-in tools and VMD. Cluster analysis of 70-100 ns trajectories for each system was executed using GROMACS' cluster module with a 1.2-nm RMSD cutoff, where the maximally populated cluster conformation was selected as the final assembled structure (Figure 2B).

Fabrication of microfluidic chips

The microfluidic chip used to synthesize MLNP and CDN-MLNP has herringbone structure, two inlets and one

outlet. The main body channel is 200 μm wide and 90 μm depth. The width and height of the herringbone structure are 200 μm and 10 μm perspectively. SU-8 2100 and SU-8 2015 photoresist (MicroChem Corp) were used to fabricate the template on a silicon wafer by photolithography using masks. The surface of photoresist mold was then modified with salinization reagent (trichloro(3,3,4,4,5,5,6,6,7,7,8,8,8-tridecafluorooctyl)silane, Sigma-Aldrich) using a vacuum chamber. The microfluidic chip is made of a glass substrate, polydimethylsiloxane (PDMS) (Sylgard 184, Dow Corning Inc.) slab. Specifially, uncured PDMS was poured onto the photoresist template and degassed for 10 min. The PDMS was cured in an 80 °C oven for 1 h. The cured PDMS slab was assembled on the glass slide after oxygen plasma surface treatment for 60 s.

Preparation of CDN-MLNP and determination of CDN encapsulation efficiency

The synthesis was initiated from solutions containing starting materials introduced by syringe pumps (PHD Ultra, Harvard Apparatus). Lipids (lipMTO : AOL : cholesterol : DMG-PEG = 50 : 25 : 20 : 5, w : w; equivalent to the molar ratio of 26 : 46.7 : 26.2 : 1) were dissolved in ethanol with the concentration of 10 mg/mL and CDN was dissolved in pH 6 PBS at 0.4 mg/mL. Then lipid solution and CDN solution were injected from the two inlets of the microfluidic chip with the flow rate at 10 mL/h and 40 mL/h, respectively. The obtained CDN-LNP solution was purified through dialysis against sterile PBS, using dialysis tubes (MWCO 6-8 kDa, Pur-A-Lyzer™Midi, PURD60010, Sigma Aldrich). For the fabrication of CDN-loaded liposome, we replaced the MOAO with the equal molar amount of DOTAP (1,2-Dioleoyl-3-trimethylammonium-propane, a cationic lipid) in the fabrication and maintained other components and preparation conditions unchanged. To determine the encapsulation efficiency of CDN, the CDN concentrations in the purified CDN-MLNP solution and unpurified CDN-MLNP solution were detected using liquid chromatography with tandem mass spectrometry (LC-MS/MS, Q-Exactive™, ThermoFisher Scientific, Sustech 15005370).

Characterization on size, Zeta potential and morphology of NPs

The hydrodynamic diameter and Zeta potential of NPs were measured by dynamic light scattering (DLS, Zetasizer Nano ZS, Malvern Instruments Ltd., UK). The morphology of NPs was observed with a transmission electron microscopy (TEM) with an acceleration voltage of 100 kV (HT7700, HITACHI, Japan); before imaging, 6 μL of NPs suspension (100 μg/mL) was dropped onto a carbon film-coated grid and then air-dried at room temperature.

In vitro drug release kinetics

3 mL of CDN-MLNP in pH 7.4-PBS or pH 5.6-PBS, with or without 50 U/mL porcine liver esterase (PLE) ,were added into dialysis bags (molecular weight cutoff of 7 kDa, Solarbio) and dialyzed against 15 mL PBS (pH 7.4), using 50-mL tubes. The 50-mL tubes were continuously shaken in an shaker at 37 °C. At predetermined time points, the releasing buffer was collected to determine MTO concentrations using a spectrofluorophotometer (RF-5301PC, SHIMADZU). The CDN concentrations in the releasing buffer were determined using the liquid chromatography with tandem mass spectrometry (LC-MS/MS, Q-Exactive™, ThermoFisher Scientific). The accumulative released percentages of MTO and CDN were calculated according to the determined concentrations.

***In vitro* cell uptake and endo/lysosomal escape**

4T1 cells were seeded in glass bottom dishes (2×10^5 cells per dish). After 24-hour cultivation, cells were stained with LysoTracker Green (Invitrogen, 100 nM) for 1 h. Subsequently, the cells were co-cultured with CDN-MLNP or CDN-liposomes at 5 μg/mL in DMEM culture medium for 2 or 6 h. The cells were washed with PBS for two times and imaged with a confocal laser scanning microscopy (Nikon, ECLIPSE Ti2-E, Japan) using the 488 nm (endo/lysosome) and 640 nm (MTO signal) laser excitation.

Cell apoptosis and immunogenic cell death in vitro

A total of 2×10^5 cells were seeded in each well of a 6-well plate. After a 24-hour incubation, CDN (2 μg/mL), MTO (2 μg/mL), MTO+CDN (2+2 μg/mL), unloaded MLNP and CDN-MLNP (equivalent to 2 μg/mL MTO) were added to the culture medium. After another 24-hour incubation, both adherent cells and suspended cells were collected using the trypsin (Gibco) and washed with PBS via centrifugation. To evaluate the cell apoptosis, the cells were resuspended in Binding Buffer and stained with the AnnexinV-FITC/PI Double Staining Cell Apoptosis Detection Kit (C1062M, Beyotime). The stained cells were then analyzed using the FITC and PE561 channels of the flow cytometry (BD FACSCanto SORP). To analyzing the immunogenic cell death, the collected cells were fixed by 4% paraformaldehyde for 20 min at room temperature, and then suspended in staining buffer (420201, Biolegend) after the centrifugation. After staining with calreticulin antibody (Alexa Fluor® 488 Conjugate), the cells were washed by the staining buffer and analyzed using a flow cytometry. To determine the immunogenic cell death of live 4T1 cells at different time, the cells on a 6-well plate were treated with different formulations for 6, 12 or 24 h. We then removed the mediums and slightly washed by the staining buffer for two times. Then the calreticulin antibody (Alexa Fluor® 488 Conjugate)-containing staining buffer was added to the wells for a 30-min incubation at 4 °C. A confocal microscope (Nikon, ECLIPSE Ti2-E, Japan) was used

to observe the fluorescent signal of membrane calreticulins.

In vitro evaluation of maturation of mouse bone marrow-derived dendritic cells (BMDCs)

For the in vitro investigation of DC maturation, BMDCs were obtained from the femurs and tibiae of 8-week-old Balb/c female mice. After the lysis of red blood cells, the collected cells were incubated with complete Roswell Park Memorial Institute (RPMI) 1640 medium and 20 ng/ml granulocyte-macrophage colony-stimulating factor (GM-CSF) along with 10 ng/ml IL-4. The adherent cells were harvested for further experiments on day 7. 4T1 cells were pretreated with each formulation for 24 h. Then, the BMDCs (5×10^5 cells per well) and the pretreated 4T1 cells (1×10^5 per well) were co-cultured for 48 h. The maturation of BMDCs was determined using flow cytometry after staining with anti-CD11c-APC (dilution of 1:100) and anti-CD86-FITC (dilution of 1:100) antibodies.

Co-culture of human immune cells and tumor cells in vitro

15 mL of human whole blood specimen were obtained from one healthy volunteer under the permission of the Medical Ethics Committee of Southern University of Science and Technology (20240212). PBMCs were isolated using Ficoll density gradient centrifugation. Firstly, blood was diluted with an equal volume of PBS and mixed slowly. A centrifugal tube with Ficoll Paque Plus reagent (ref. 17144002, Cytiva) was prepared and diluted blood was carefully overlayed to begin density gradient separation. Centrifugation was performed with the break-off (2000 rpm, 20 min), and then the white PBMC layer was carefully collected. Three centrifugal steps were performed for washing with PBS. The obtained PBMCs were seeded in the 24-well plates at 3×10^5 cells/0.5 mL/well density. The culture medium was RPMI-1640 medium containing FBS and PS and supplemented with 50 ng/mL recombinant human macrophage colony-stimulating factor (M-CSF) and 20 ng/mL IL-2 (ref. 21212-5, Pepro Tech). After 72 hours of culture, the monocytes have differentiated into adherent macrophages. To complete the medium change, the supernatant (containing T cells) was collected, centrifuged, resuspended in the culture medium and added evenly to each macrophage well. MDA-MB-231 cells were pretreated with different formulations (PBS, CDN, MTO, CDN+MTO, MLNP and CDN-MLNP) for 4 h or 24 h, and the concentration of each formulation was the same as that for evaluating ICD above. The tumor cells were then labeled with CFDA-SE and added to the above wells (1.5×10^5 cells/0.5 mL/well) to form the co-culture system of tumor cells, macrophages and other immune cells.

To evaluate the phagocytosis of MDA-MB-231 cells by human macrophages in the co-culture system, the

supernatants were removed and the adhesion cells were slightly washed for 3 times with PBS after 4 hours of co-culture. Then, the cells were digested and collected using 4-fold diluted trypsin. Twice centrifugation and wash were performed using the staining buffer (420201, Biolegend). The pacific blue-labeled CD14 antibody was used to label macrophages. A flow cytometry (CytoFLEX SRT, Beckman) was used to evaluate the phagocytosis of MDA-MB-231 cells by human macrophages. The phagocytosis was calculated as the percentage of double-positive macrophages among blue CD14-positive macrophages. To observe the phagocytosis using the confocal microscopy, the same experimental process was performed in the 96-well plate, in which the cell plating densities of PBMCs and MDA-MB-231 cells were adjusted to 5×10^4 cells/0.1 mL/well and 2.5×10^4 cells/0.05 mL/well, prospectively. The macrophages (blue) incorporating green fluorescent signals the indicated they phagocytized the cancer cells.

To evaluate the T-cell response and STING activation in the co-culture system, the co-culture was continued for 48 h. We collected the supernatants, and washed the wells with medium for 3 times and collected the washing liquids. The cells in supernatants together with the washing liquids were combined and underwent twice centrifugation/wash using the staining buffer, then analyzed with a flow cytometry (CytoFLEX SRT, Beckman) after staining with anti-human CD8a (PE) and anti-human CD4 (Pacific Blue) antibodies. The adhesion cells (including macrophages and live tumor cells) were lysed to extract the total RNA for real-time quantitative PCR (RT-qPCR), using a Cell/Tissue Total RNA Isolation Kit (RC112-01, Vazyme, Nanjing, China). 0.5 microgram of total RNA was transcribed into complementary DNA (cDNA) using High-Capacity cDNA Reverse Transcription Kit (R323-01, HiScript III RT SuperMix for qPCR, Vazyme, Nanjing, China), and RT-qPCR was performed using Taq Pro Universal SYBR qPCR Master Mix (Q712-02, Vazyme, Nanjing, China) with primers. The messenger RNA levels *Ifnb1*, *Cxcl9* and *Cxcl10* were detected to confirm the activation of STING pathways.

In vitro evaluation of STING activation in bone marrow-derived macrophages (BMDMs)

For evaluating the STING activation in mouse macrophages, we obtained bone marrow-derived macrophages (BMDMs) from femurs and tibiae of 8-week-old Balb/c female mice. After the lysis of red blood cells, the collected cells were incubated with complete Roswell Park Memorial Institute (RPMI) 1640 medium and 20 ng/ml macrophage colony-stimulating factor (M-CSF). The adherent cells were harvested for further experiments on day 7. 4T1 cells were pretreated with each formulation for 24 h. Then, the BMDCs (5×10^5 cells per well) and the pretreated 4T1 cells (2.5×10^5 per well) were co-cultured for 24 h. Then, the

supernatants were collected for determining the IFN-β concentration using an ELISA kit (Elabscience, No. E-EL-M0033), and all the adherent cells were collected for western blot (WB). For the WB assay, 100 μL of pre-chilled RIPA Lysis Buffer (P0013B, Beyotime, https://www.beyotime.com) was added, and the cells were incubated on ice for 5 minutes before being scraped gently. The cell lysate was then centrifuged at 14000 × g and 4 oC for 10 minutes, and the supernatant was collected. Total protein content was measured using a BCA Protein Assay Kit (P0012, Beyotime). Protein samples were mixed with Blue Loading Buffer Pack (7722, Cell Signaling Technology) and heated at 95-100 oC for 10 minutes. After a brief centrifugation at 14000 × g for 1 minute, 10 μL of the sample was loaded onto a 15-well SDS-PAGE 4-20% Gradient Gel (ET15420Gel, ACE Biotechnology, https://www.ebio-ace.com) with a protein marker (DB180, MIKX, http://www.mikxlife.com). Electrophoresis was conducted using MOPS-SDS Running Buffer (F00004Gel, ACE Biotechnology) at 100 V for 80 minutes. The gel was then transferred to a PVDF membrane (BSP0161, PALL) using a transfer buffer at 300 mA for 90 minutes. The membrane was blocked with 5% non-fat powdered milk (A600669, Sangon Biotech) in TBST (Tris-Buffered Saline with 0.1% Tween-20) for 1 hour, followed by overnight incubation with primary antibodies at 4 °C. After washing three times with TBST, the membrane was incubated with secondary antibodies at room temperature for 1 h, followed by three additional washes with TBST. Finally, the membrane was developed using either Hypersensitive ECL Western HRP Substrate (17046, ZENBIO, http://www.zen-bio.cn) or Clarity Western ECL Substrate (1705060, Bio-Rad) and visualized with a ChemiDocTM MP Imaging System (Bio-Rad). All primary antibodies and HRP-linked anti-rabbit IgG are from a Mouse-Reactive STING Pathway Antibody Sampler Kit (Cell Signaling Technology, No. 16029T). HSP90 rabbit monoclonal antibody was from Cell Signaling Technology (No. 4877).

In vivo bio-distribution and tumor penetration

To visualize tumor delivery in vivo, subcutaneous 4T1 tumor-bearing mice (n = 3, ~100 mm^3) were intravenously injected once with CDN-MLNP and CDN-loaded liposomes (50 μg MTO per mouse). At 24 h, tumors, heart, liver, spleen, lung and kidneys were imaged using a In Vivo Imaging System (PerkinElmer, IVIS Spectrum, USA). For the tumor penetration investigation, tumors were embedded in O.C.T. (SAKURA Tissue-Tek® O.C.T, 4583) and frozen in liquid nitrogen for section preparation. Tumor tissues were cut into 10 μm slide sections. After fixing with 4% PFA for 30 min, the blood vessels in the tumor were stained with a primary anti-CD31 antibody (ab281583, 1:100, Abcam) followed by an Alexa Fluor 488-conjugated secondary antibody (ab150077, 1:500, Abcam). DAPI (4,6-diamidino-2-phenylindole) was used to localize the cellular nuclei. The

sections were then observed using confocal laser scanning microscopy (Nikon, ECLIPSE Ti2-E, Japan).

To analyze the cell uptake of CDN-MLNP in tumor, subcutaneous 4T1 tumor-bearing mice (n = 3, ~100 mm^3) were intravenously injected once with CDN-MLNP (50 μg MTO per mouse). At 24 h, the tumors were isolated and dissociated through both mechanical and enzymatic dissociating methods (Hanks' balanced salt solution (HBSS) containing collagenase IV (1 mg/mL, Gibco), hyaluronidase (0.1 mg/mL, Yeasen, 20426ES60) and DNase I (20 U/mL, Coolaber, SL2076)). The obtained single cell suspension was centrifuged (400 g, 5 min, 4 °C) and washed using HBSS. The red blood cells were lysed using the RBC Lysis Buffer (BioLegend, 420301) for 5 min on ice. The cell suspension was centrifuged (400 g, 5 min, 4 °C) and washed using the staining buffer (BioLegend 420201). Count viable cells and resuspend in staining buffer at 2×10^7 cells/ml. 50 μL of cell suspension was added to 50 μL of staining buffer containing fluorescence-labeled flow antibodies, and incubate on ice in the dark for 20 minutes. Wash two times with 1ml of cell staining buffer by centrifugation at 400 g for 5 minutes. The cell pellet was resuspended in 0.5 ml of cell staining buffer and added DAPI to exclude dead cells, then performed flow cytometric analysis. The tissue sample treatment and cytometric analysis in followed assays are the same, unless otherwise stated.

In vivo pharmacokinetic study

Balb/c mice were intravenously (i.v.) injected with free CDN+MTO mixture or CDN-MLNP at an equivalent CDN dose of 20 μg per mouse. Plasma samples were collected at 10 min, 30 min, 1, 3, 6 and 24 h post injection. For LC–MS detection, 20 μl plasma was digested in 80 μl of 0.1 M HCl solution for 10 min followed by the addition of 400 μl MeOH–acetonitrile (1:1). The solutions were cooled using ice and then centrifuged for 10 min at 14,000 g. The supernatants were collected and then dried with nitrogen gas, resuspended in 50 μl of nuclease-free water. MTO concentration was quantified using fluorescence measurement (Ex: 660 nm, Em: 687 nm), while CDN concentration were determined by LC-MS. Pharmacokinetic analysis including terminal half-life ($t_{1/2}\beta$) and area under curve (AUC) was used a two-phase exponential decay model in GraphPad Prism 10.

In vivo evaluation of innate immune response and biosafety

We established a new cohort of 4T1 tumor-bearing Balb/c mice. When tumors reached approximately 100–200 mm^3 (day 7 post-inoculation), mice were intravenously administered different formulations on day 7, 10, and 13, including PBS, CDN (20 μg per mouse), MTO (40 μg per mouse), MTO+CDN (40 μg MTO +

20 μg CDN per mouse), MLNP (equivalent to 40 μg MTO per mouse), CDN-LNP (equivalent to 20 μg CDN per mouse) and CDN-MLNP (equivalent to 20 μg CDN per mouse). Mice were sacrificed 6 h after the third dose to capture early innate immune responses (or inflammatory responses) and acute toxicity. Blood samples were collected from 4T1 tumor-bearing mice (n = 3 per group) 6 h after the third intravenous dose via cardiac puncture under isoflurane anesthesia. Each blood sample was divided into two tubes: one containing EDTA-K_2 as an anticoagulant for complete blood count (CBC) analysis, and another without anticoagulant for serum separation by centrifugation (3000 rpm). CBC parameters, including white blood cells (WBC), lymphocytes, red blood cells (RBC), and platelets, were measured using an automated hematology analyzer (Dymind, DF52). Serum was separated and used for analysis of cytokine and serum biochemistry. Serum biochemistry, including alanine aminotransferase (ALT), alkaline phosphatase (ALP), creatinine (CR), and blood urea nitrogen (BUN), was assessed using a biochemical analyzer (Meikang, MS-480). The serum cytokines were detected using ELISA kits, including IFN-β (Elabscience, No. E-EL-M0033), TNF-α (Servicebio, GEM0004) and IL-6 (Servicebio, GEM0001). In addition, spleen, liver and kidney tissues were collected, fixed in 4% paraformaldehyde, embedded in paraffin, sectioned, and stained with hematoxylin and eosin (H&E) for histopathological evaluation.

Investigations of therapeutic efficacy and anti-tumor immune response in vivo

For subcutaneous 4T1 tumor-bearing mice, BALB/c mice (n = 5 or 6) were subcutaneously injected with 5×10^5 4T1 cells in 100 μl of PBS/Matrigel (1/1, v/v, Corning Matrigel, 354262) at the right flank. When tumors grew to 100-200 mm^3 in size, the mice received three doses (one dose per three days) of intravenously administered PBS, free MTO (50 μg per mouse), free CDN (20 μg per mouse), unloaded MLNP (0.2 mg per mouse) or CDN-MLNP (equivalent to 20 μg CDN, 32.8 μg MTO, and 0.162 mg CDN-MLNP per mouse). Mice with tumor volumes greater than 1000 mm^3 were considered dead because 4T1 tumors larger than 1000 mm^3 tend to be ulcerated and were not in compliance with the ethical welfare of animals in our laboratory animal center.

In an independent study, 4T1-tumor bearing mice received a single intratumoral injection of PBS, CDN, MTO+CDN and CDN-MLNP. At 6 h or 24 h post-injection, the tumors were isolated and dissociated into single cell suspensions as the above dissociating methods. The cellular calreticulin expression and the proportions of CD4 and CD8-positive T cells in CD45-positive cells were analyzed by the flow cytometry after staining with

flow antibodies.

In an independent study, 4T1-tumor bearing mice received a single intravenous or subcutaneous injection of PBS, MTO, CDN, unloaded MLNP and CDN-MLNP. At 12 h post-injection, the tumors were isolated and lysed to extract the total RNA for real-time quantitative PCR (RT-qPCR) to confirm the expressions of Ifnb1 and Cxcl10 genes. A Cell/Tissue Total RNA Isolation Kit (RC112-01, Vazyme, Nanjing, China) and a low temperature tissue grinder (ServiceBio, KZ-5F-3D) were used. The assays including cDNA reverse transcription and qPCR were the same with above description.

In an independent study, we established the 4T1 tumor-bearing mouse model and performed intratumoral administration of the following seven agents: PBS (control), free CDN, free MTO, free MTO+CDN mixture, unloaded MLNP (containing lipMTO but no CDN), CDN-LNP (containing CDN but no lipMTO, with DOPE replacing lipMTO at an equimolar concentration), and CDN-MLNP (containing both CDN and lipMTO). The dose administered intratumorally is half that administered intravenously. Tumors were collected at 6 and 24 h post-injection for flow cytometric analysis, and serum was collected for detection of IFN-β level. For disassociation of 4T1 tumor, tumors were excised from mice, minced into small pieces (approximately 1–2 mm^3) using a scissor, and transferred to a digestion cocktail containing collagenase IV (1 mg/mL, Gibco), hyaluronidase (0.1 mg/mL, Yeasen, 20426ES60) and DNase I (20 U/mL, Coolaber, SL2076) in hank's balanced salt solution (HBSS). The tissue suspension was incubated at 37 °C for 30–45 min with gentle shaking (150 rpm). After digestion, the mixture was filtered through a 70 μm cell strainer (Falcon, USA) to remove undigested debris. The filtered cell suspension was centrifuged at 400 × g for 5 min at 4 °C, and the supernatant was discarded. The red blood cells were lysed using the RBC Lysis Buffer (BioLegend, 420301) for 5 min on ice. Cells were then washed by centrifugation using HBSS, and finally resuspended in the cell staining buffer (Biolegend, 420201) and subsequent staining with flow cytometric antibodies.

To construct orthotopic KPC pancreatic ductal adenocarcinoma (PDAC) tumor model, 5×10^5 luciferase-engineered KPC cells were orthotopically transplanted into the pancreas head of a Balb/c nude mouse through a surgery with an incision on the right side of the abdomen. At 7 days post-inoculation, the mice were intraperitoneally injected with D-Luciferin and imaged using an In Vivo Imaging System (PerkinElmer, IVIS Spectrum, USA) to determine the tumor sizes. The selected mice bearing tumors were treated with PBS,

unloaded MLNP (0.2 mg per mouse) or CDN-MLNP (0.1 or o.2 mg per mouse) for three times. At 16 days post-inoculation, the mice sacrificed and their tumors along with pancreas and spleen were collected for weighing. The tumors were dissociated into single cells for flow cytometry analyzing.

For subcutaneous B16-F10 tumor-bearing mice, female C57BL/6J mice were subcutaneously injected with 5×10^5 4T1 cells in 100 μl of PBS/Matrigel at the right flank. The mice received two doses (at day 6 and day 9) of intravenously administered PBS, free MTO (50 μg per mouse), free CDN (20 μg per mouse), MLNP (0.2 per mouse) or CDN-MLNP (equivalent to 20 μg CDN, 32.8 μg MTO, and 0.162 mg CDN-MLNP per mouse). After six days (day 15) of the second dose, tumors were completely resected and the wounds were covered with gauzes. The tumors were dissociated into single cells and analyzed by flow cytometry. To analyze the IFNγ expression, the cells were fixed with 4% PFA for 20 min at room temperature and permeabilized using iced 90% methanol, and then washed with cell staining buffer for two times before staining with flow antibodies. To construct the rechallenge model, we injected 1×10^5 B16-F10 cells through tail vein at day 40. After another 10-day, the mice were sacrificed and their lungs were collected to confirm the lung metastasis of tumor cells. At the same time, the spleens were collected and prepared single cell suspension through mechanical trituration. Specifically, the spleen was placed on a 100 μm cell strainer on a 10 cm dish, added 5 mL hank's balanced salt solution (HBSS), grinded the spleen with a grinding rod, and collected the filtrate. Then the flow cytometry was performed to analyze T cells in the spleen in the same methods as above.

Statistical analysis

Data were presented as the means ± SD. The in vitro experiments were performed in independent experiments with at least three repetitions for each group. All statistical analysis of the data was determined by t-tests or one-way analysis of variance (ANOVA) using GraphPad Prism 10 software, and a P value less than 0.05 was considered statically significant.

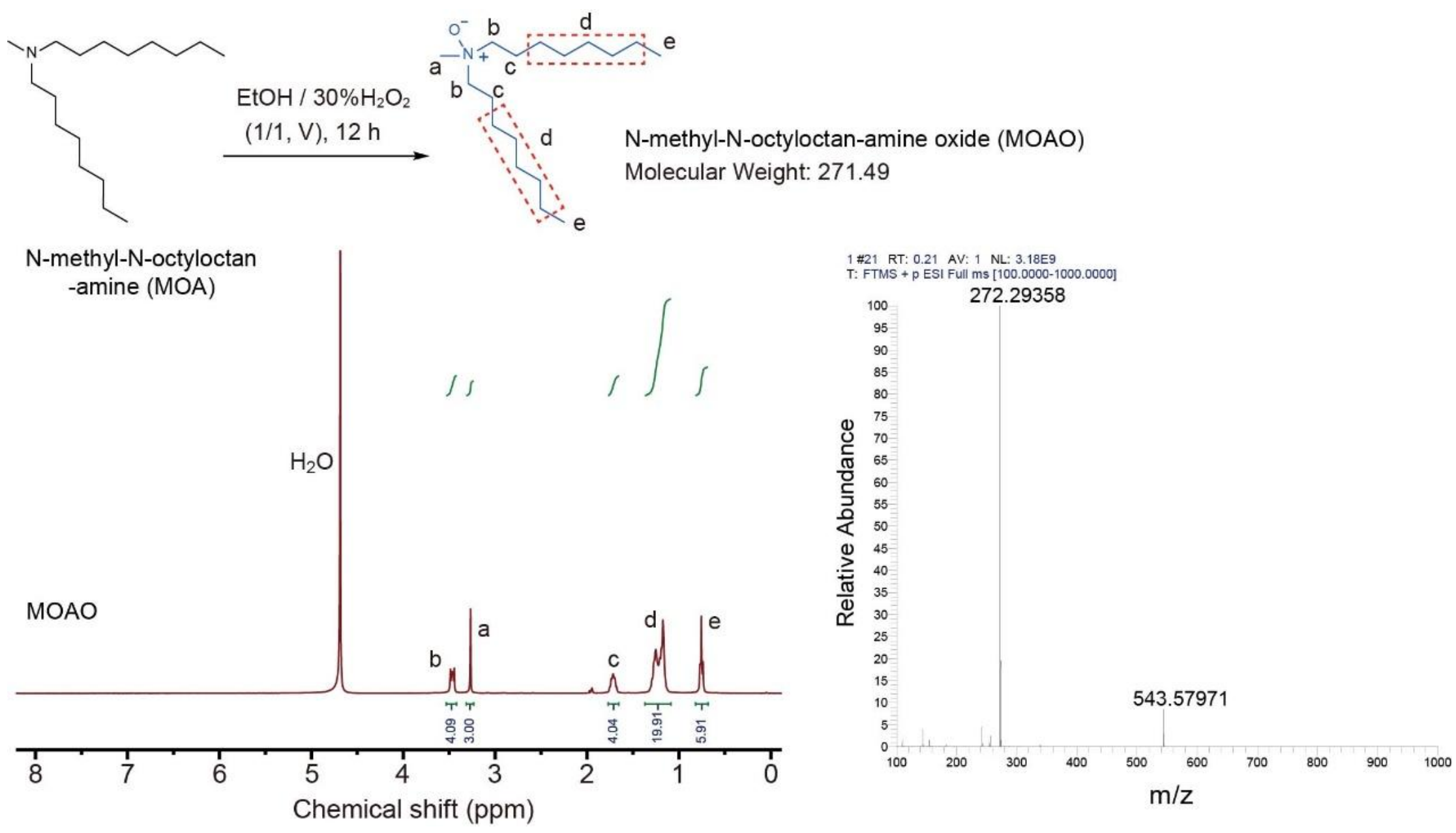

Figure S1. The synthesis of N-methyl-N-octyloctan-amine oxide (MOAO), and its ^{1}H NMR spectrum and high-resolution mass spectrum.

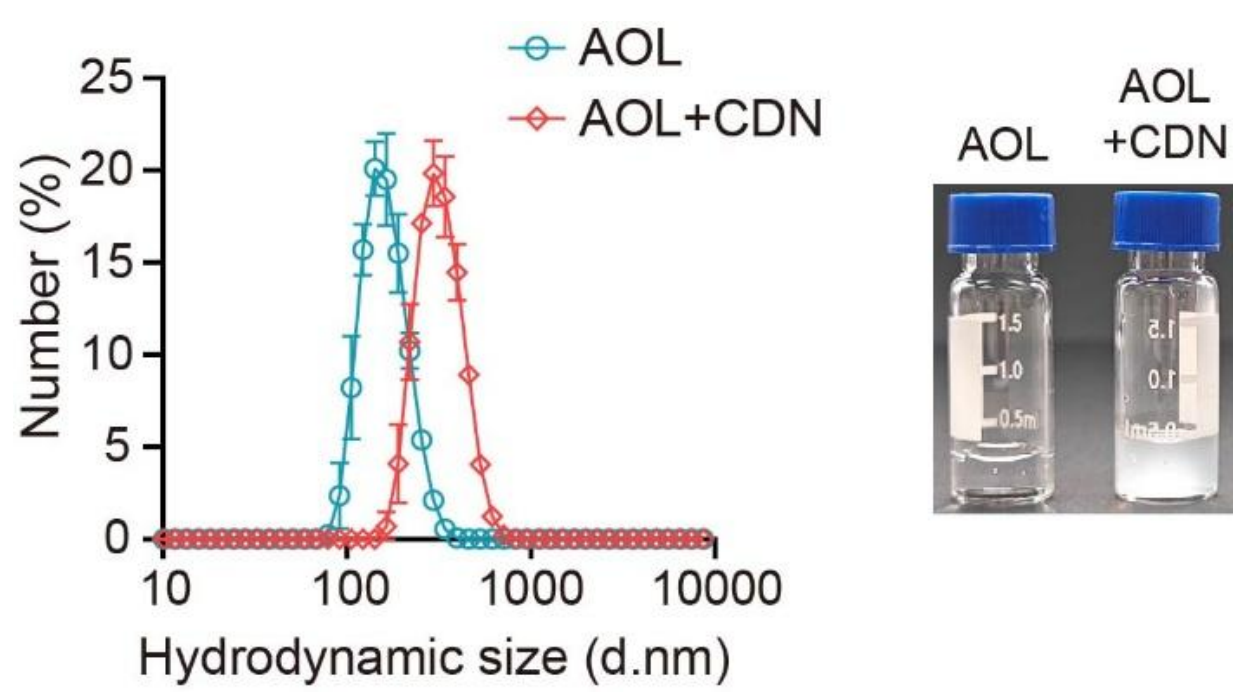

Figure S2. The sizes of AOL micelle and AOL/CDN emulsion in the pH 4.35 acetic acid/sodium acetate buffer determined with dynamic light scattering (DLS). The right photo shows that adding CDN into the clear AOL solution enables the formation of white emulsion in seconds at this pH.

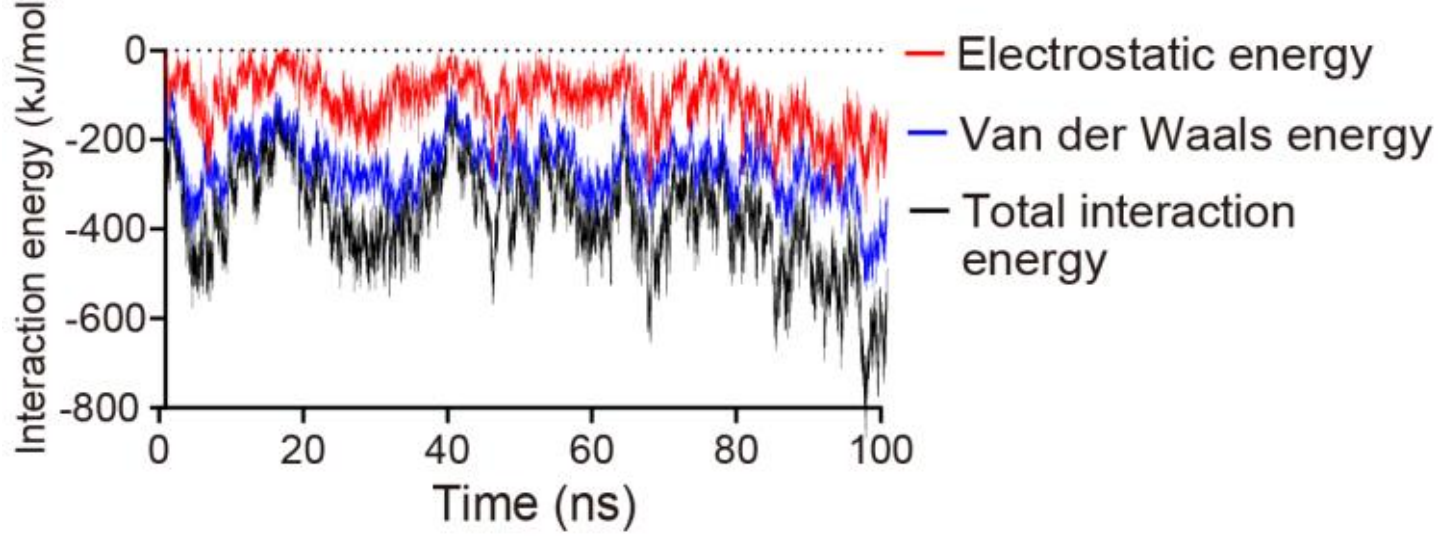

Figure S3. Time-dependent intermolecular interaction energy evolution process of the whole system

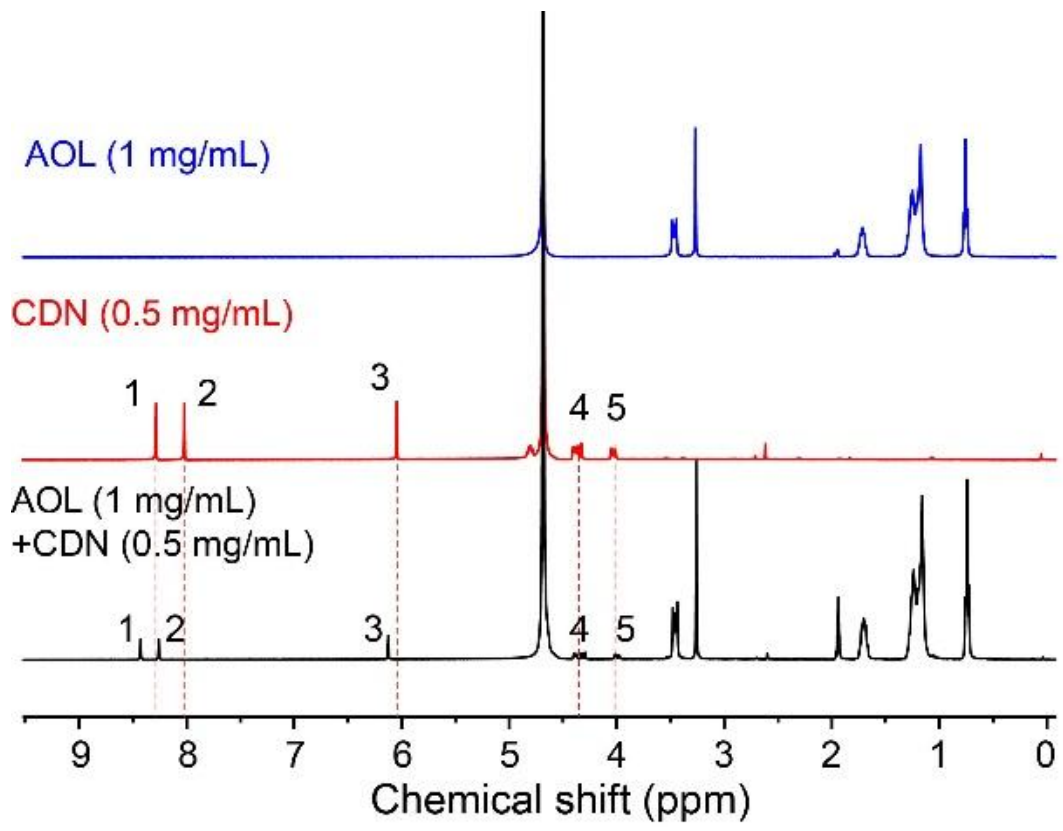

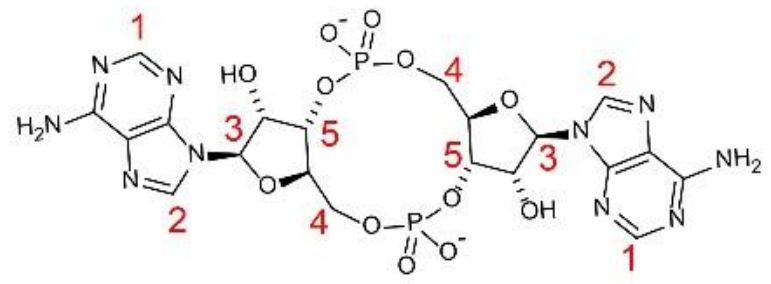

	δ (H1)	δ (H2)	δ (H3)	δ (H4)	δ (H5)
CDN	8.28	8.02	6.05	4.37	4.03
AOL+CDN	8.43	8.26	6.12	4.35	4.00
Δδ	0.15	0.24	0.07	-0.02	-0.03

Figure S4. The ^{1}H NMR spectra of the AOL, CDN and the mixture of both. The right table shows the chemical shifts (δ) of CDN protons in the ^{1}H NMR spectra of CDN alone and the mixture. Δδ refers to the differences in chemical shifts between CDN protons and the corresponding protons in the mixture.

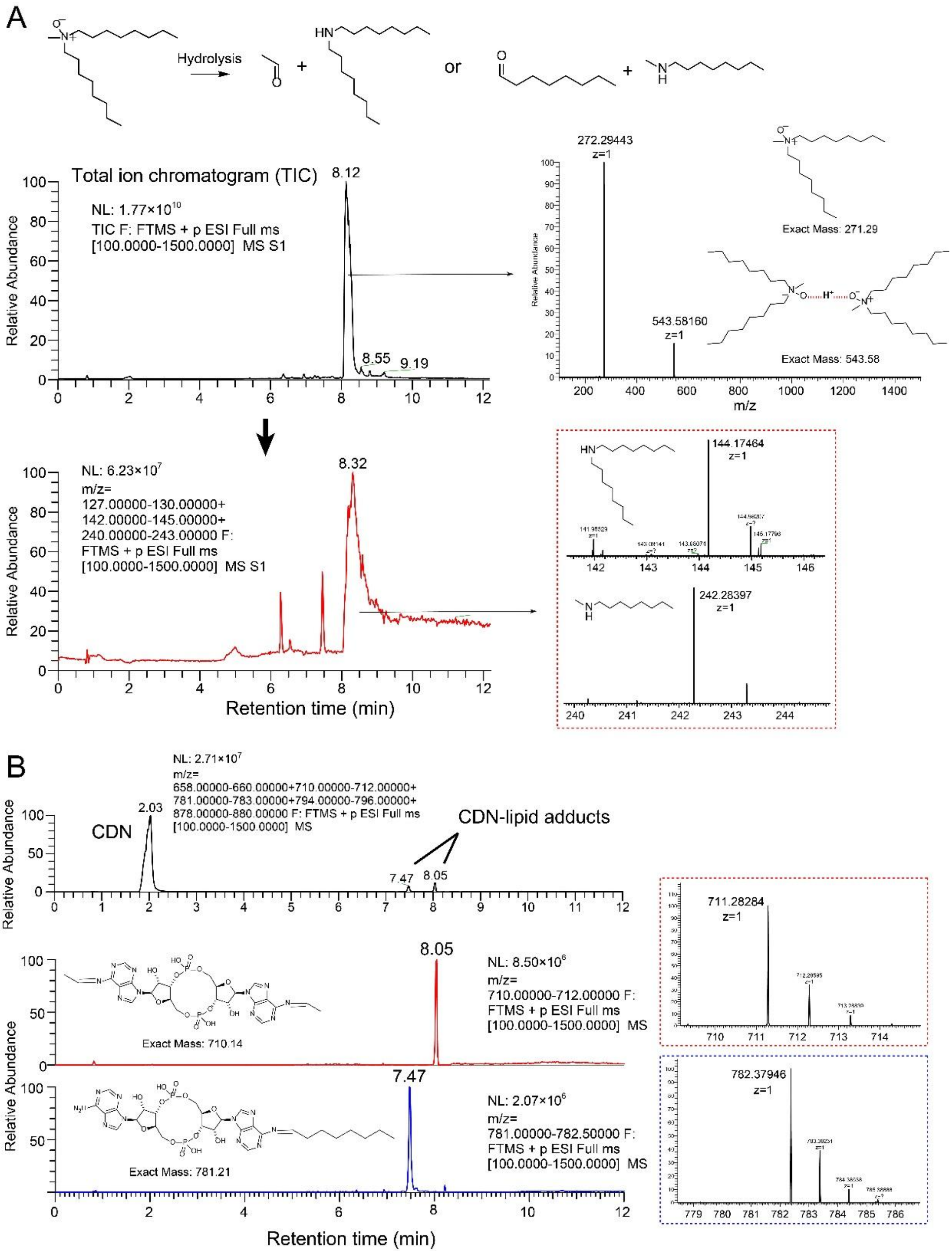

Figure S5. HPLC-MS analysis of the AOL and CDN mixture. A solution containing AOL (4 mM) and CDN (1 mM) in PBS (pH 7.4 or 5.6) was incubated at the room temperature for 48 h, followed by a 20-fold dilution with methanol for LC-MS analysis. (A) LC-MS spectra showing the hydrolysis of AOL. (B) Ion chromatograms and corresponding MS spectra of CDN and related CDN-lipid adducts, demonstrating the formation of CDN-lipid adducts.

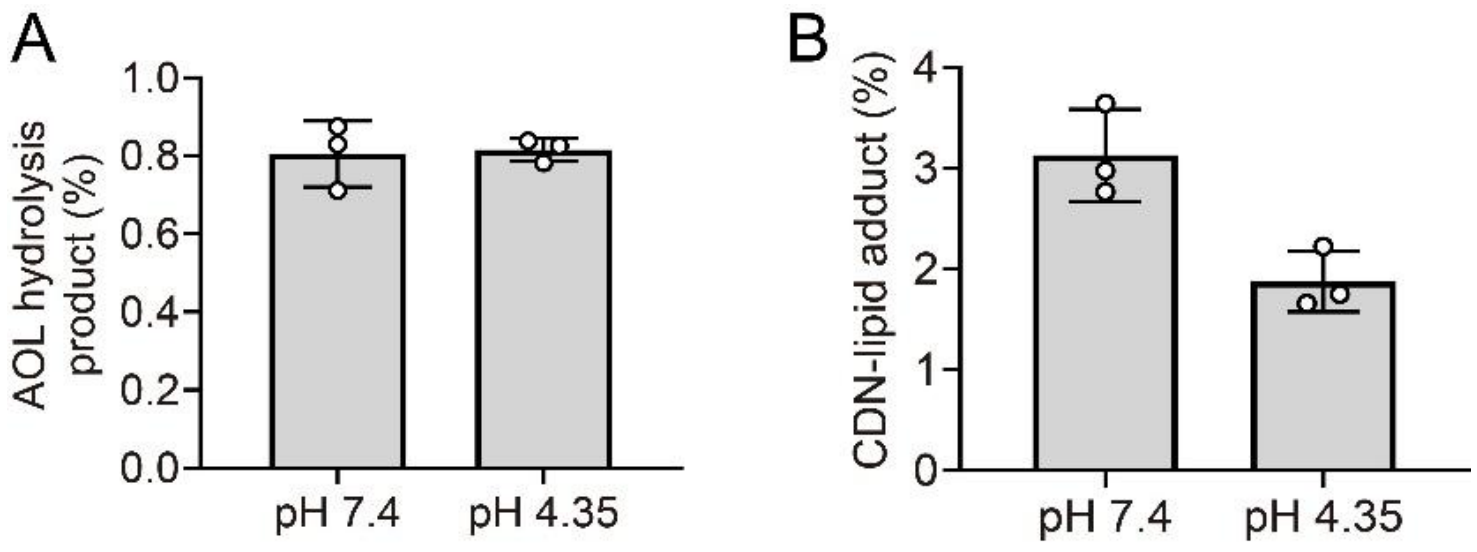

Figure S6. (A) Proportion of AOL hydrolysis products (relative to total AOL) in the AOL/CDN solution at pH 7.4 and 4.35. (B) Proportion of CDN-lipid adducts (relative to total CDN) in the solution.

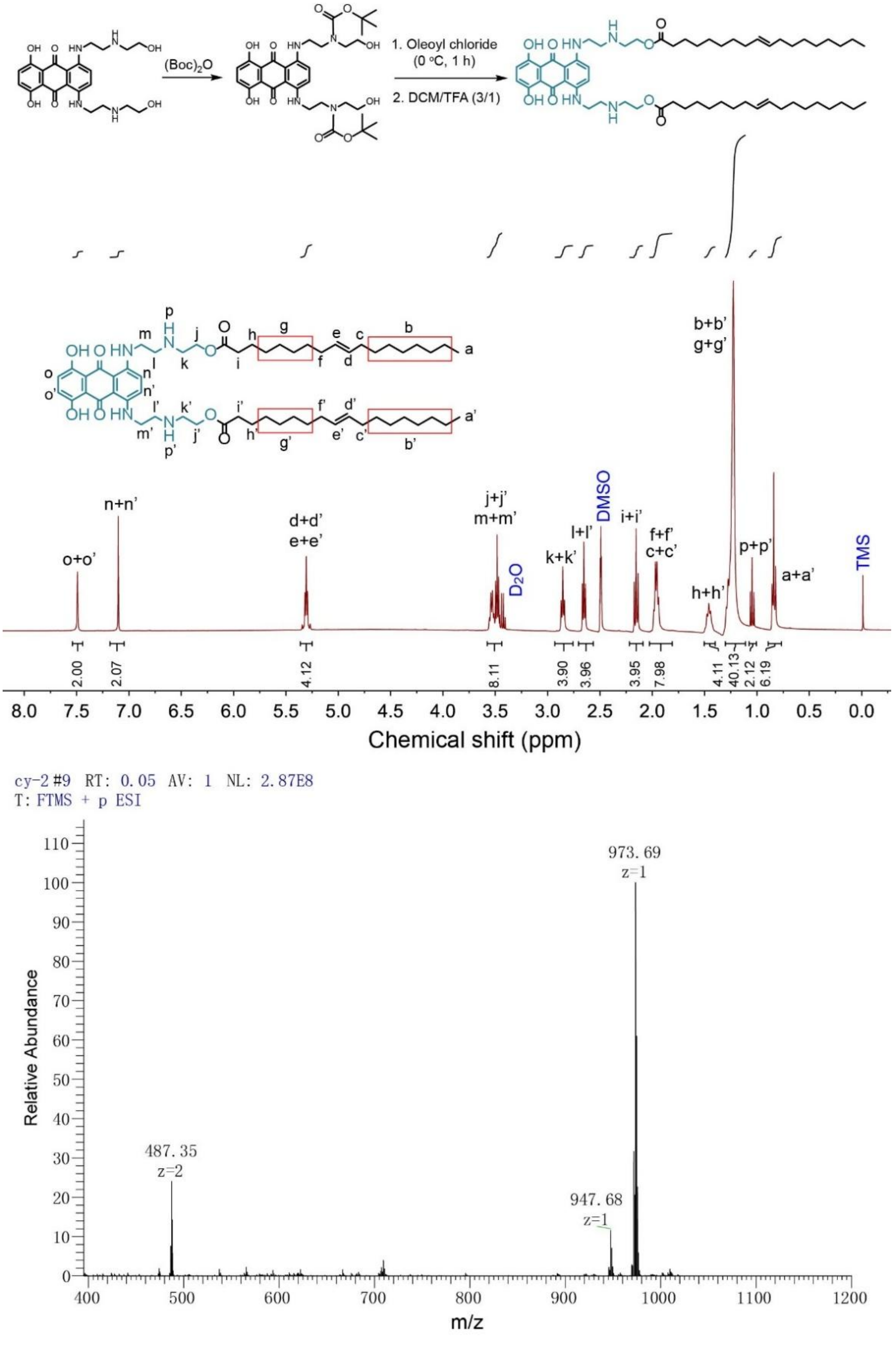

Figure S7. The synthesis of oleoyl-conjugated mitoxantrone (lipMTO), and its ^{1}H NMR spectrum and high-resolution mass spectrum.

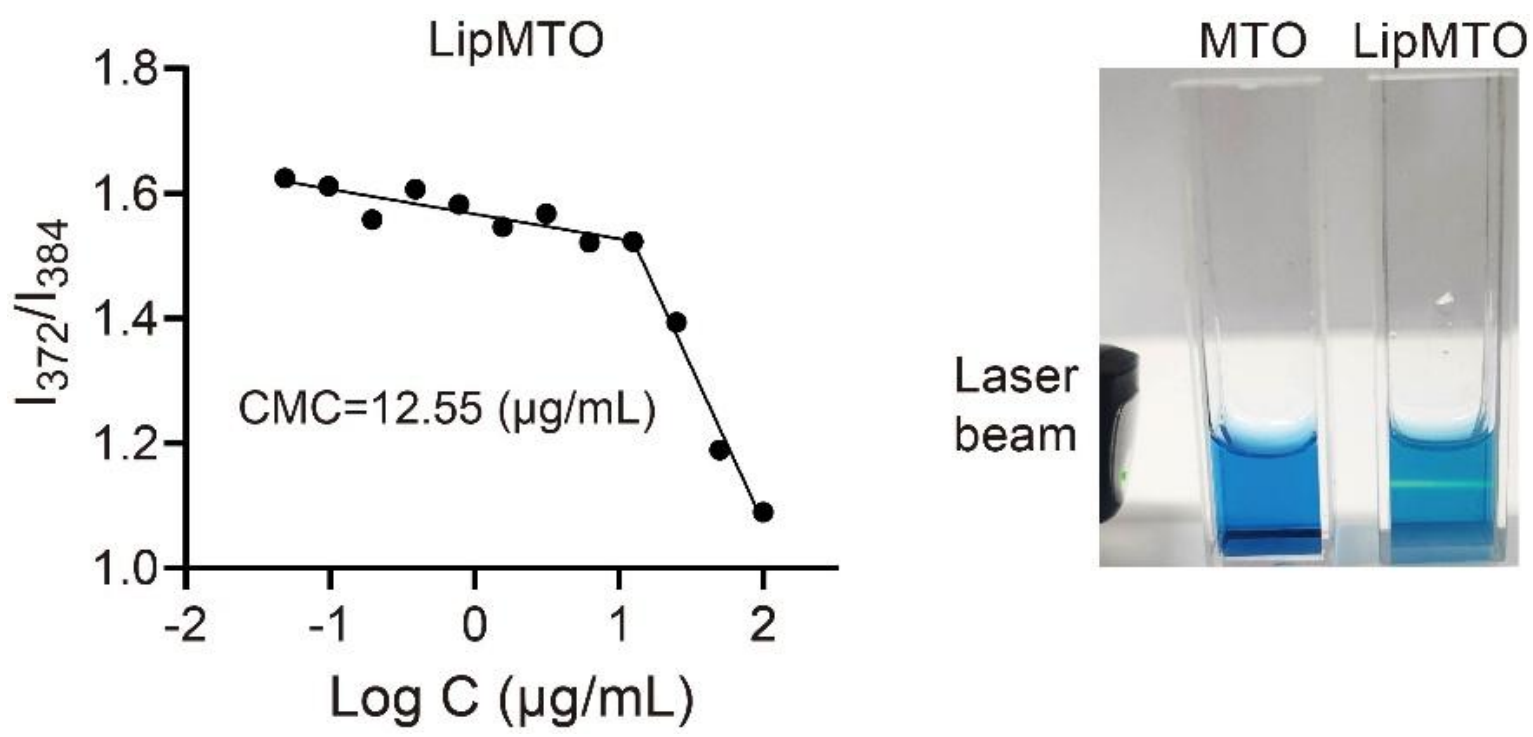

Figure S8. The determination of critical micelle concentrations (CMC) of lipMTO and the photo of lipMTO and MTO solutions under the laser light.

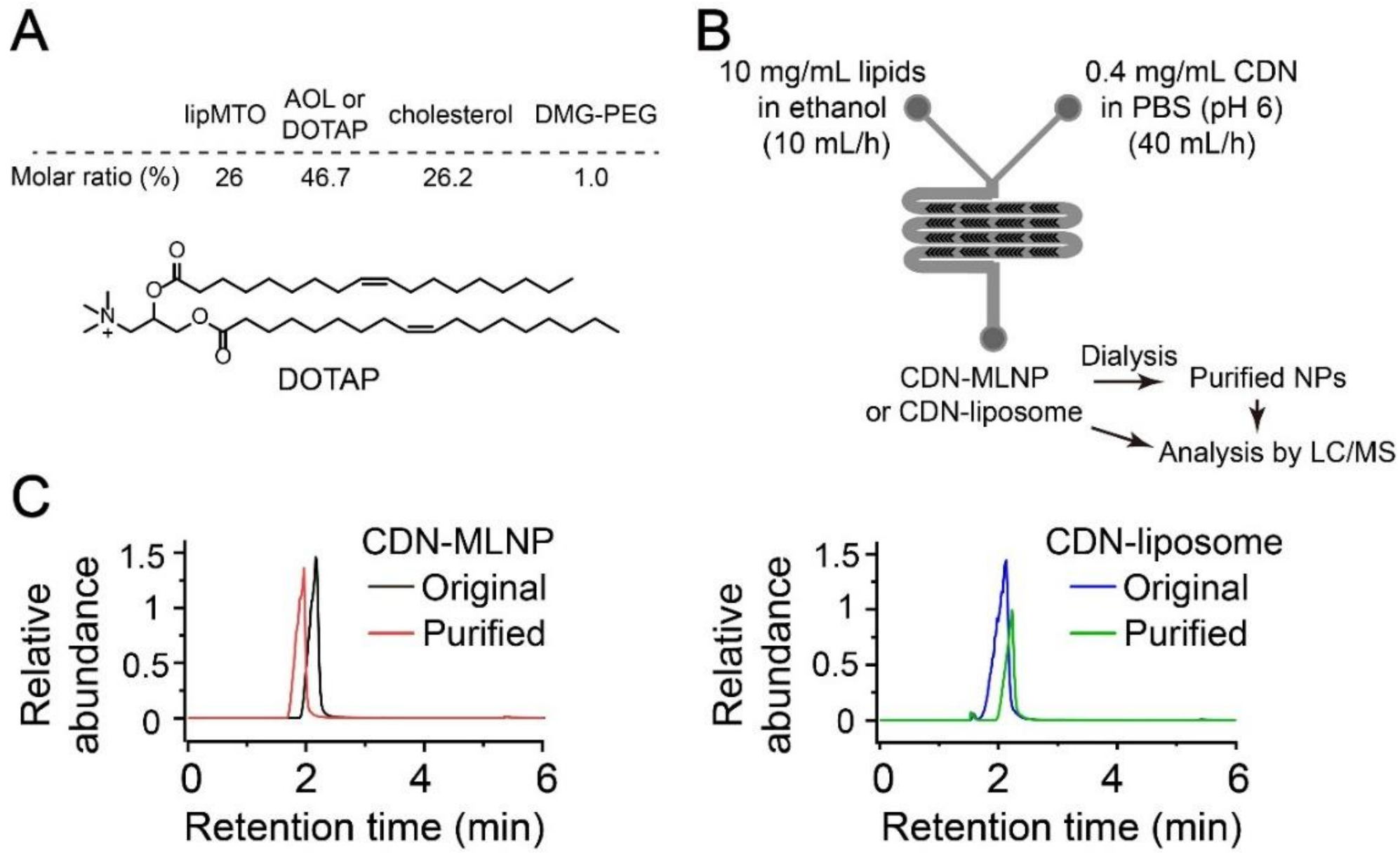

Figure S9. The formulations of CDN-MLNP and CDN-encapsulated cationic liposome (CDN-liposome). A) The feed molar percentages of four different lipids and the chemical structure of the cationic lipid, DOTAP, for the CDN-liposome. B) The microfluidic chip with herringbone textures was used to fabricate the CDN-MLNP. The feed concentration, solvent and flow rate of each component were showed. The liquid chromatography with tandem mass spectrometry (LC-MS) was used to analyze the amount of CDN and MTO in the purified nanoparticle solution. C) These chromatograms detected by LC-MS showed the relative concentrations of CDN in the CDN-MLNP or CDN-liposome solutions that were purified or unpurified using dialysis. The peaks were screened according to the m/z of CDN.

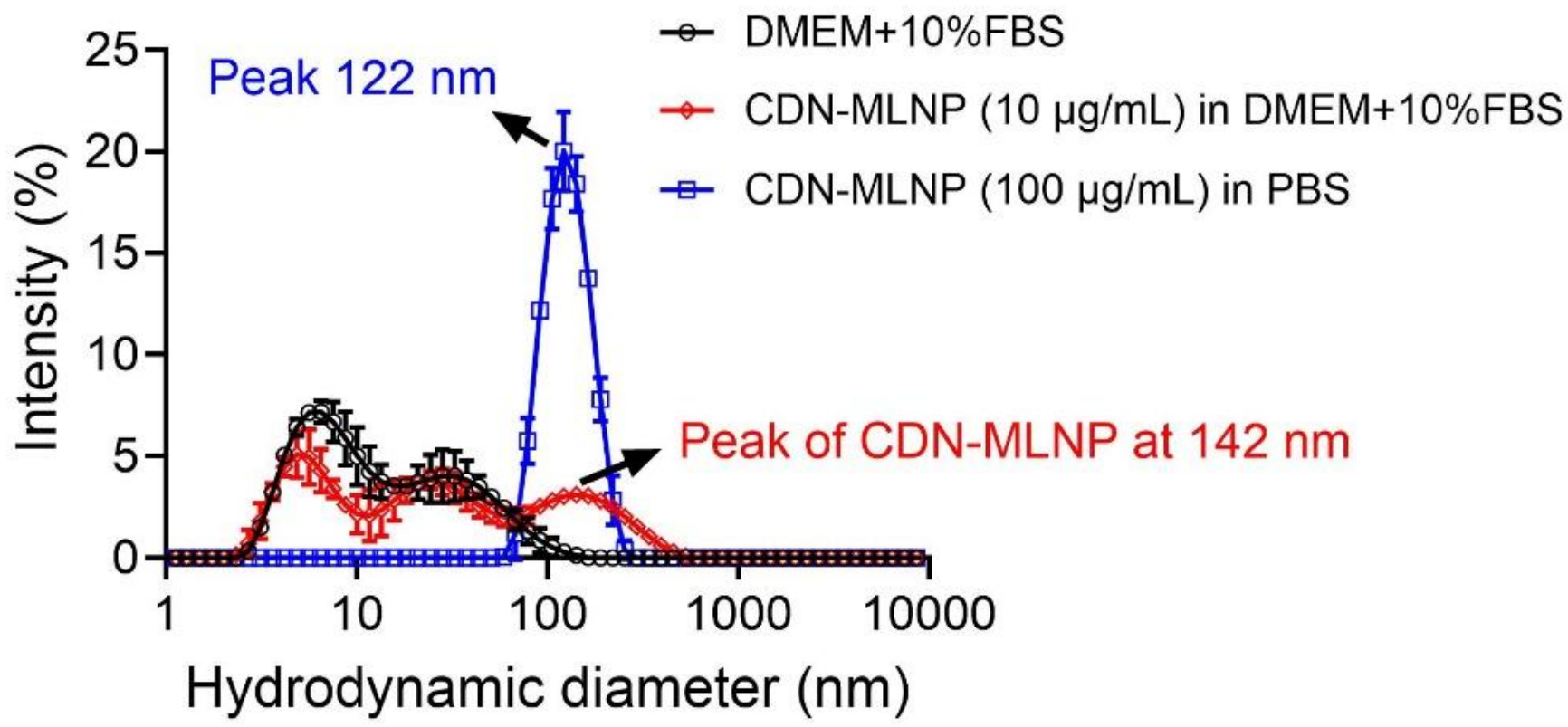

Figure S10. Intensity-weighted size distribution of CDN-MLNP in different media measured by dynamic light scattering. CDN-MLNP was dispersed in cell culture medium (DMEM supplemented with 10% FBS) at 10 μg/mL (treatment concentration) and incubated at room temperature for 12 h. Compared with CDN-MLNP in PBS, the intensity-averaged hydrodynamic diameter in DMEM+10% FBS increased by about 20 nm (Figure S12). No significant aggregation or precipitation was observed.

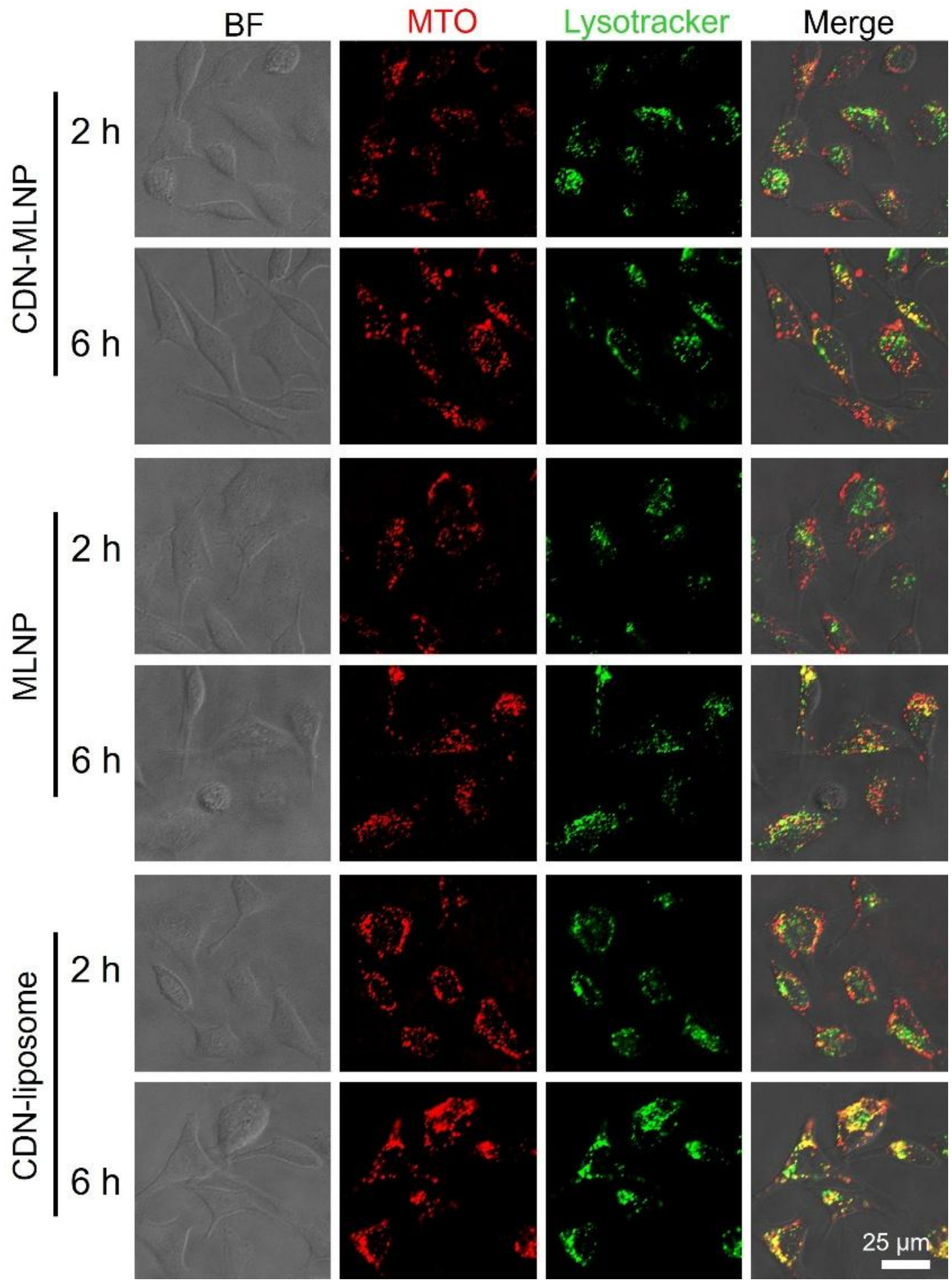

Figure S11. Confocal fluorescence images of 4T1 cells treated with CDN-MLNP, MLNP or CDN-liposome for 2 and 6 h. The fluorescence of MTO was detected as the location of NPs, and cellular acidic compartments (endo/lysosomes) were stained with LysoTracker (green).

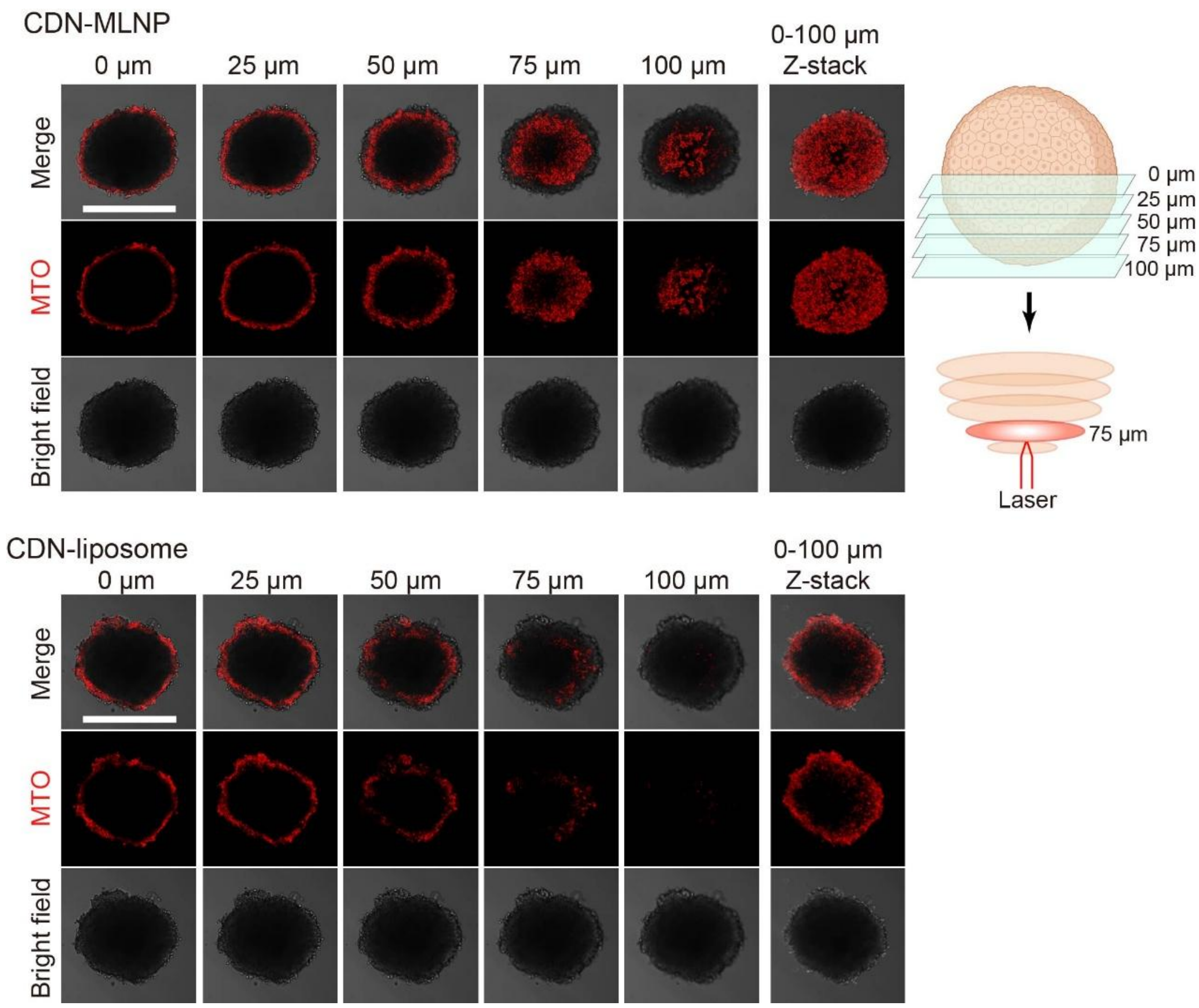

Figure S12. The confocal images of the 4T1 tumor organoid at varied depths upon incubation with CDN-MLNP or CDN-liposome for 12 h. Separate brightfield and single-channel fluorescence images and a schematic of the imaging method are showed. Scale bar 500 μm.

A

	CDN-MLNP		Free CDN+MTO	
Time (h)	CDN (μg/mL)	ID%	CDN (μg/mL)	ID%
0.167	8.81 ± 2.25	66.1 ± 16.9	0.96 ± 0.37	7.23 ± 2.78
0.5	6.10 ± 1.68	45.7 ± 12.6	0.29 ± 0.16	2.22 ± 1.23
1	3.31 ± 0.72	24.8 ± 5.43	0.017 ± 0.005	0.13 ± 0.040
3	1.35 ± 0.32	10.1 ± 2.41	-	-
6	0.65 ± 0.22	4.89 ± 1.68	-	-
24	0.27 ± 0.14	2.07 ± 1.07	-	-

B

	CDN-MLNP		Free CDN+MTO	
Time (h)	MTO (μg/mL)	ID%	MTO (μg/mL)	ID%
0.167	16.5 ± 2.01	76.4 ± 9.26	3.18 ± 0.64	11.9 ± 2.38
0.5	12.5 ± 1.69	57.8 ± 7.82	0.71 ± 0.27	2.65 ± 1.02
1	8.23 ± 1.79	38.0 ± 8.25	0.24 ± 0.049	0.91 ± 0.19
3	4.76 ± 1.71	22.0 ± 7.90	0.085 ± 0.026	0.32 ± 0.096
6	2.42 ± 0.73	11.1 ± 3.36	0.046 ± 0.009	0.17 ± 0.034
24	1.21 ± 0.56	5.60 ± 2.59	0.026 ± 0.008	0.100 ± 0.029

C

	CDN-MLNP		Free CDN+MTO	
	CDN	MTO	CDN	MTO
Area under curve, AUC (μg•h/mL)	20.85 ± 2.66	66.46 ± 9.12	0.31 ± 0.079	2.06 ± 0.18
Half life, $T_{1/2}\beta$ (h)	3.01	2.542	0.19	0.15

Figure S13. Pharmacokinetic data of CDN (A) and MTO (B) in balb/c mice injected (i.v.) with CDN+MTO mixture or CDN-MLNP. Total CDN, MTO and percentage of injected dose (ID) are represented in this table. CDN levels were determined by LC-MS, while MTO concentrations were quantified using fluorescence measurement (Ex: 660 nm, Em: 687 nm). (C) Pharmacokinetic analysis using a two-phase exponential decay model showed area under curve (AUC) and half life (T1/2β) of CDN and MTO. The data represent mean ± s.d., from a representative experiment of two independent experiments with n = 3 per group.

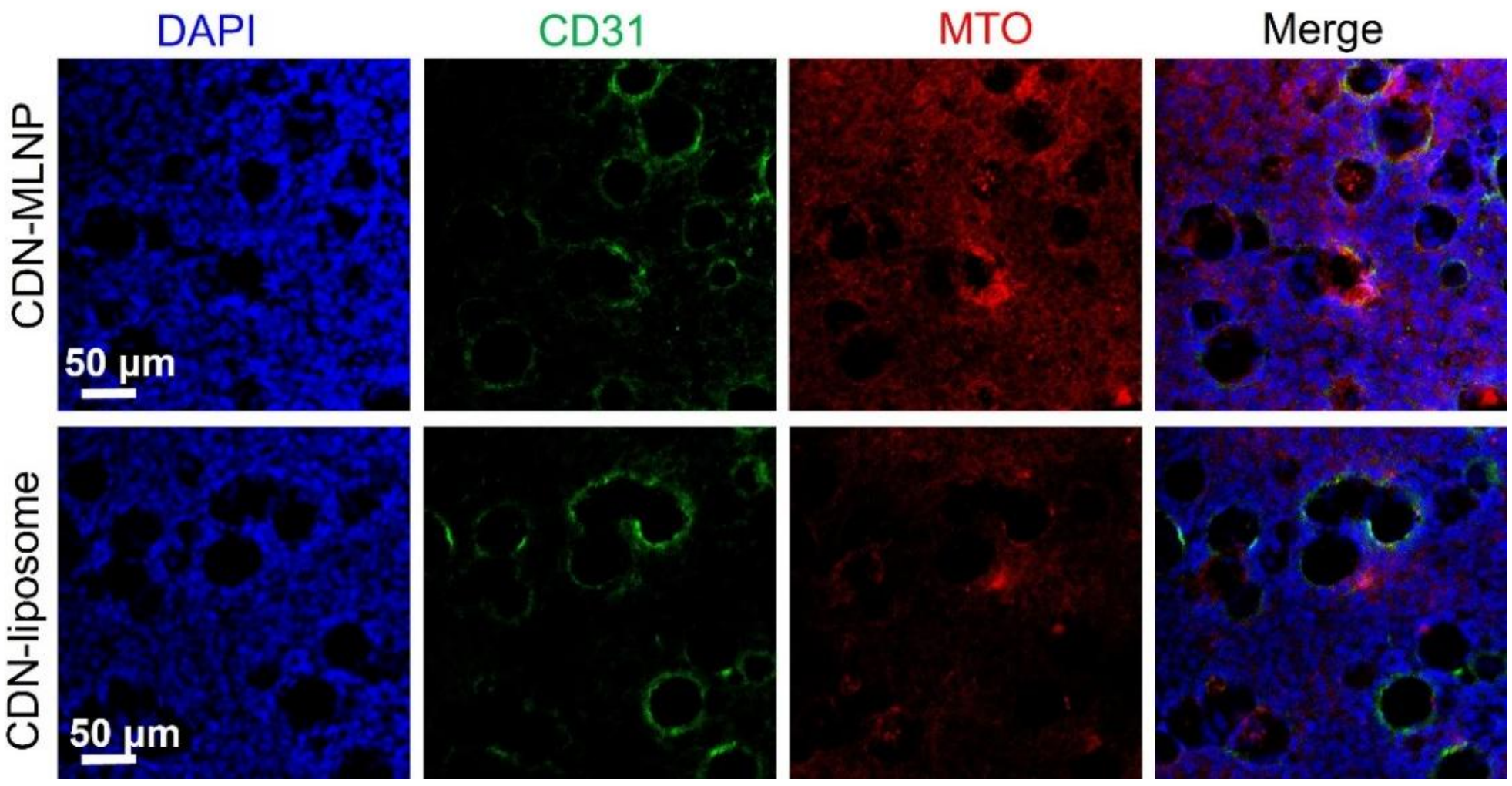

Figure S14. The accumulation and penetration of CDN-MLNP in the tumor tissue. The Investigation of the ability of CDN-MLNP to extravasate and penetrate the tumor after *i.v.* administration into mice with subcutaneous 4T1 tumors 24 h after *i.v.* injection of CDN-MLNP and CDN-liposomes, confocal laser scanning microscopy of sections of the tumors was performed. Blood vessels were marked with CD31 antibody followed by Alexa Fluor 488 secondary antibody staining (green). Cell nuclei were stained by DAPI (blue).

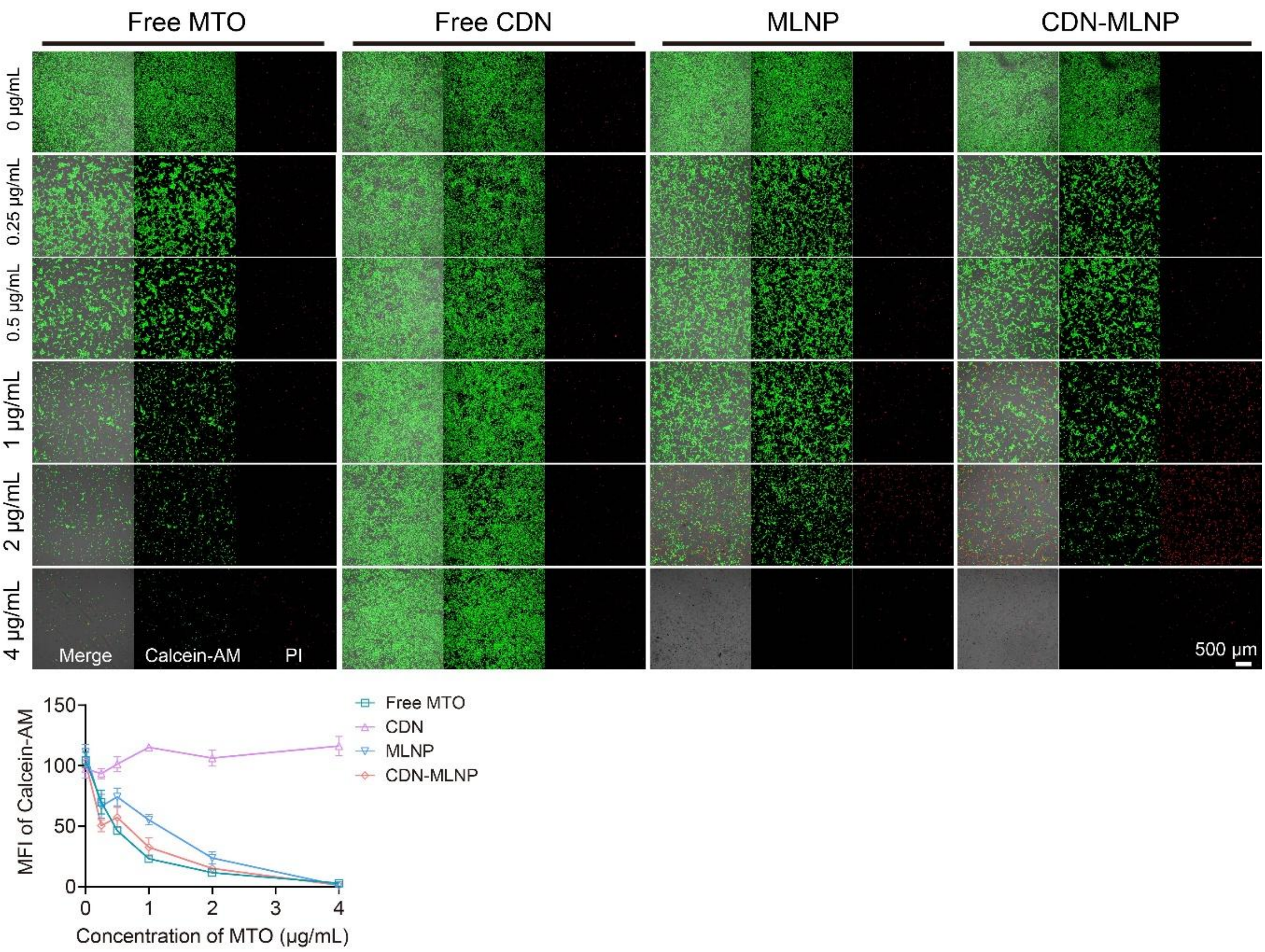

Figure S15. The cytotoxic effects of CDN-MLNP and other formulations on cancer cells. The confocal images show the live/dead conditions of 4T1 cells treated with different agents for 48 hours (staining by calcein-AM/PI). The quantification result shows the mean fluorescence intensity (MFI) of calcein-AM for staining live cells.

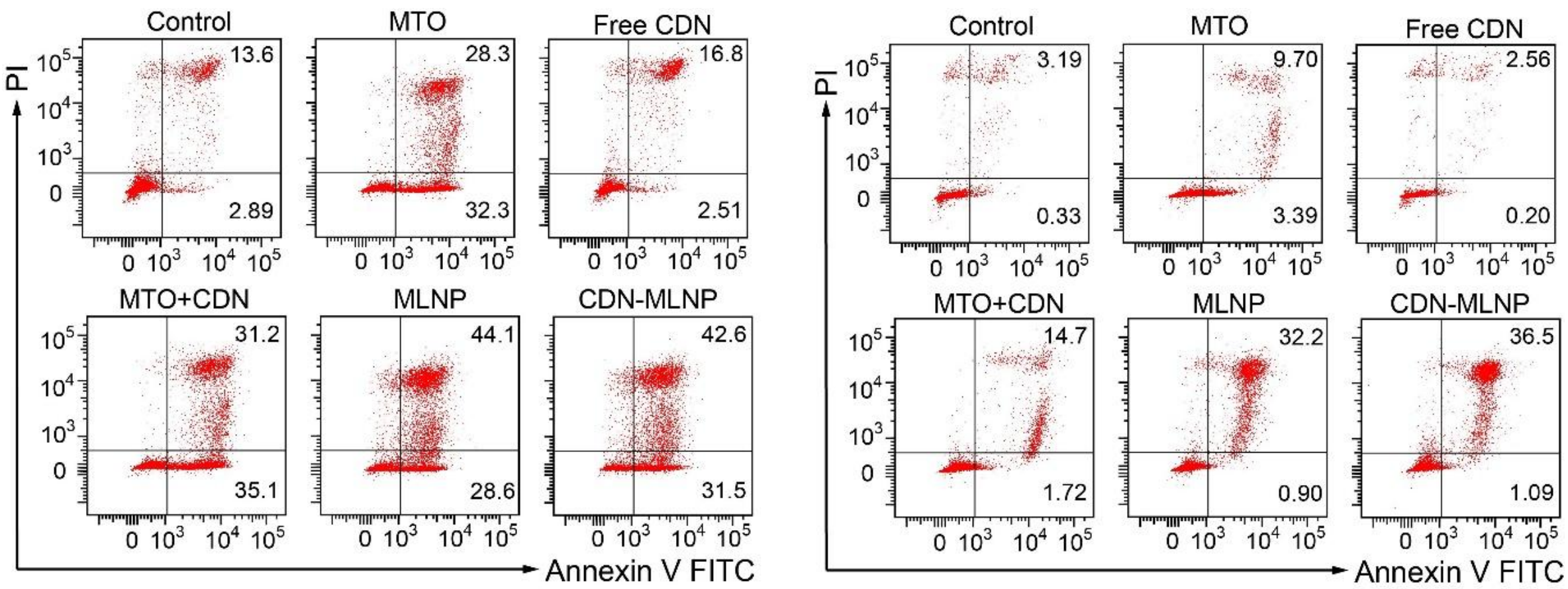

Figure S16. Flow cytometry analysis of apoptosis/necrosis of 4T1 cells (left) and B16-F10 cells (right) using Annexin-PI staining after incubation with different formulations for 24 h.

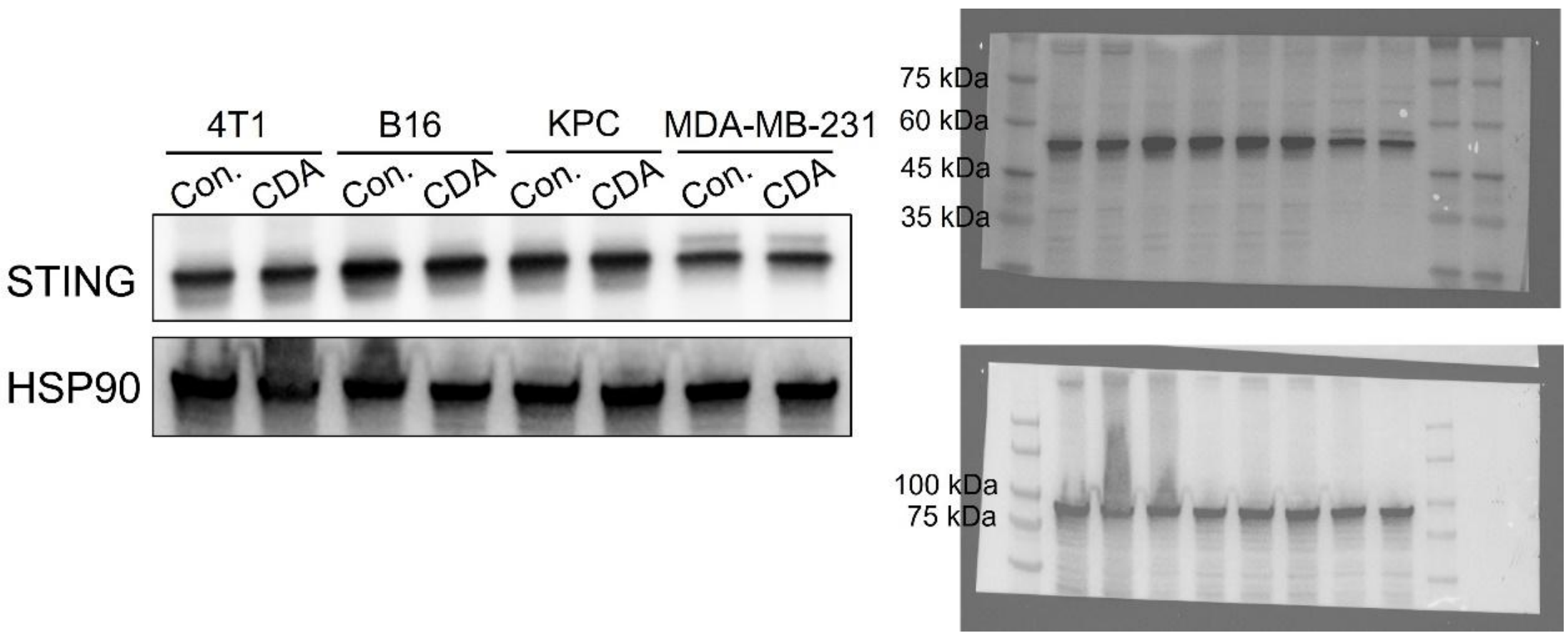

Figure S17. The western blot images show the expression of STING in four different cancer cells, including 4T1 cells (mouse triple negative breast cancer cells), B16-F10 cells (mouse melanoma cells), KPC cells (mouse pancreatic cancer cells), and KPC cells) and MDA-MB-231 cells (human triple negative breast cancer cells).

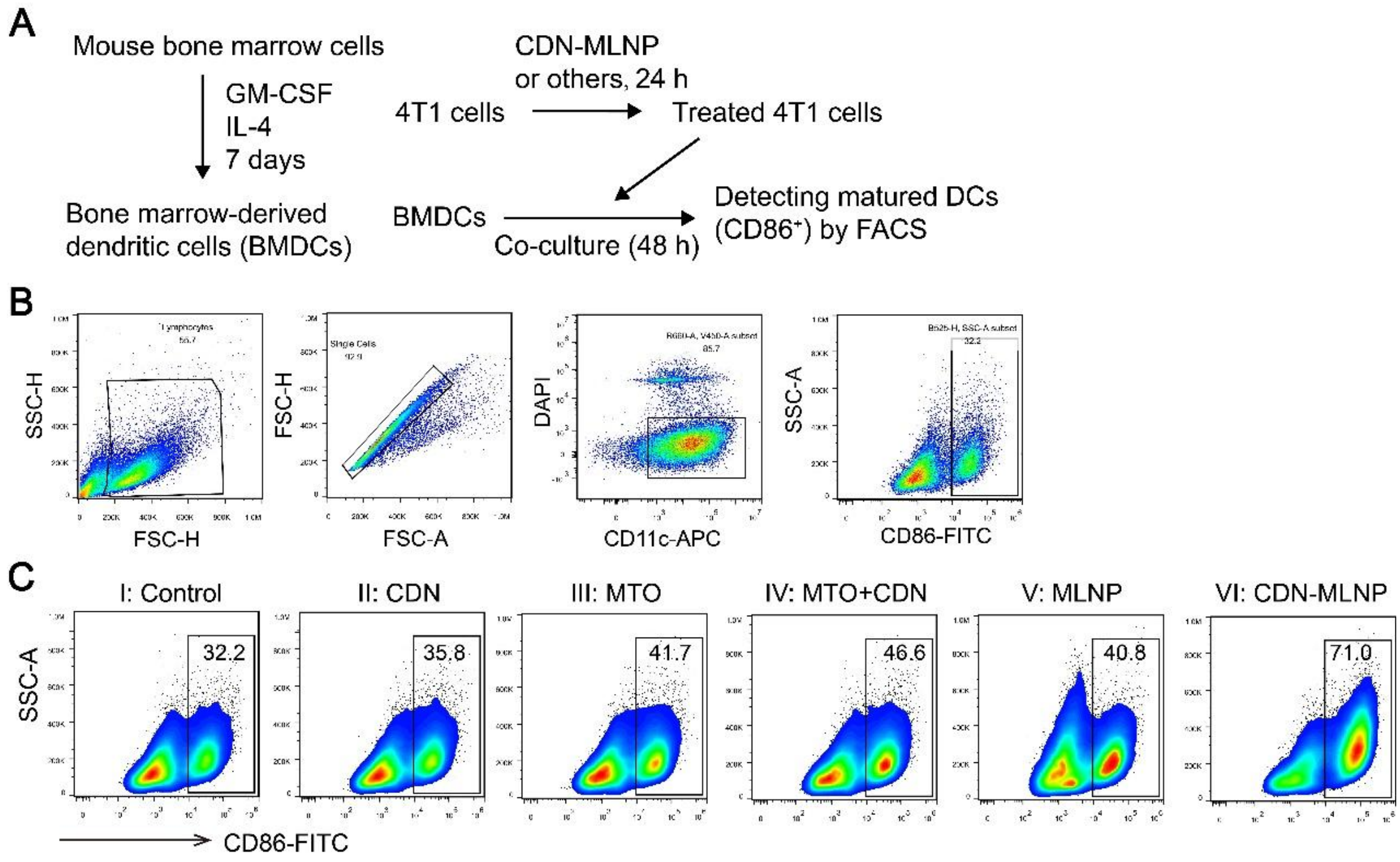

Figure S18. Flow cytometry analysis of CD86-positive BMDCs after stimulation with pretreated 4T1 cells with each formulation. A) The 4T1 cells were treated with CDN-MLNP or other formulations for 24 h, and then the pretreated 4T1 were co-cultured with BMDCs for 48 h. The CD86-positive matured BMDCs were identified using the flow cytometry. B) The flow cytometric gating strategies for analyzing the maturation of BMDCs after staining with the flowmetric antibody of CD11c, CD86 and DAPI. C) The flow cytometric plots of each group.

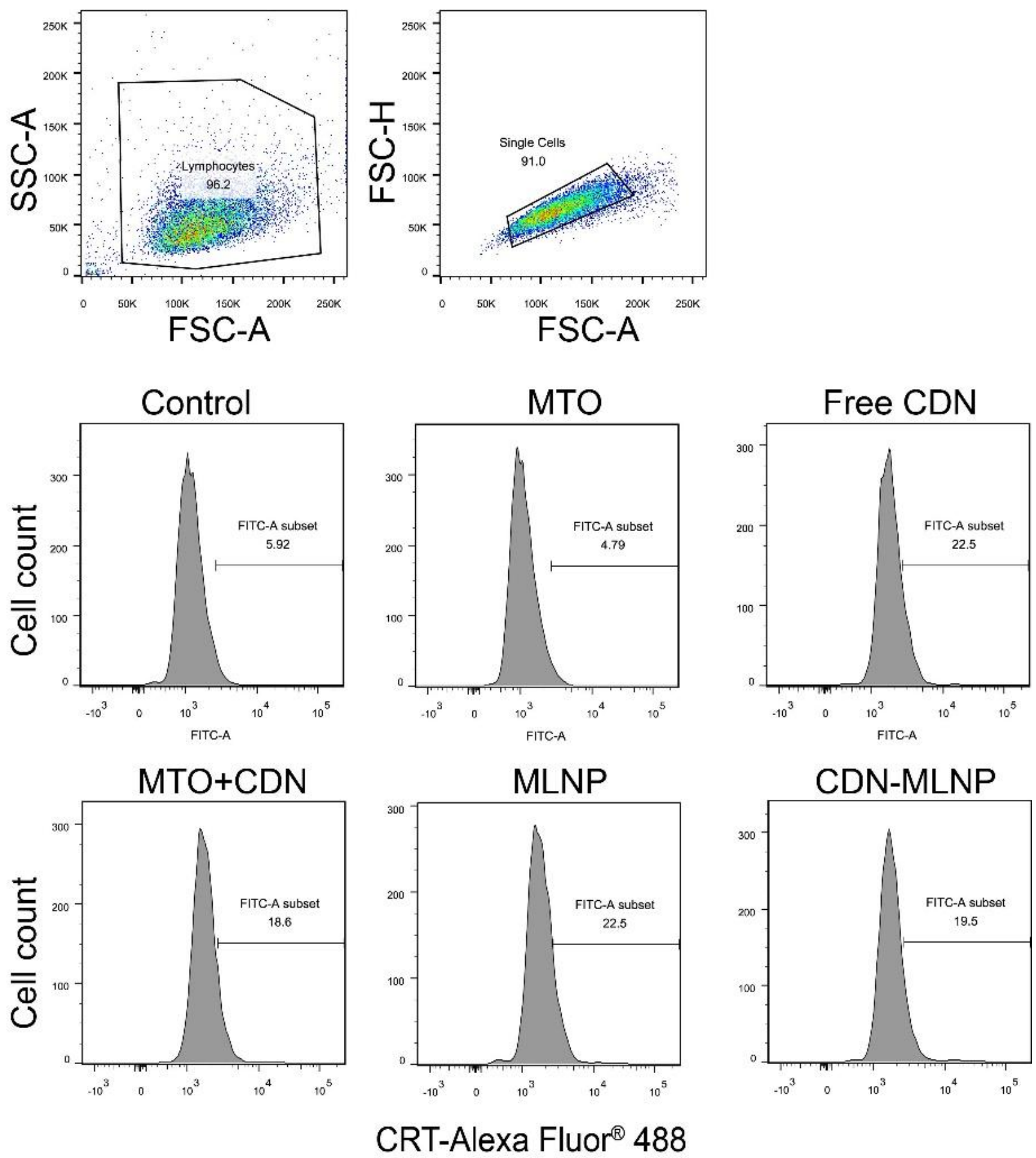

Figure S19. The flow cytometry gating strategies for analyzing the expression level of calreticulin (CRT) of 4T1 cells treated with different formulations for 24 h, and the related flow cytometry counter diagrams.

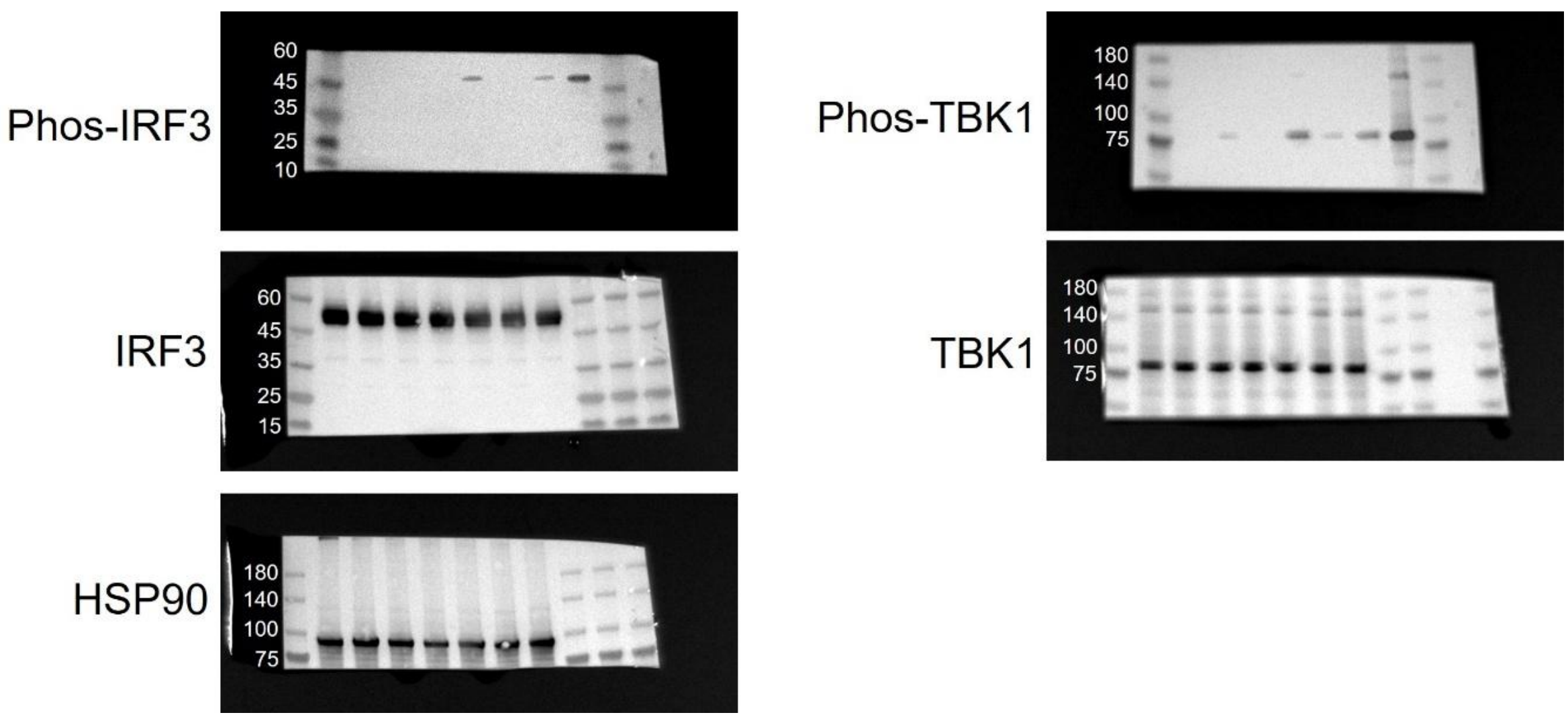

Figure S20. Uncropped membrane for western blotting of maker proteins in STING-IRF3 pathway. Corresponding to Figure 4E.

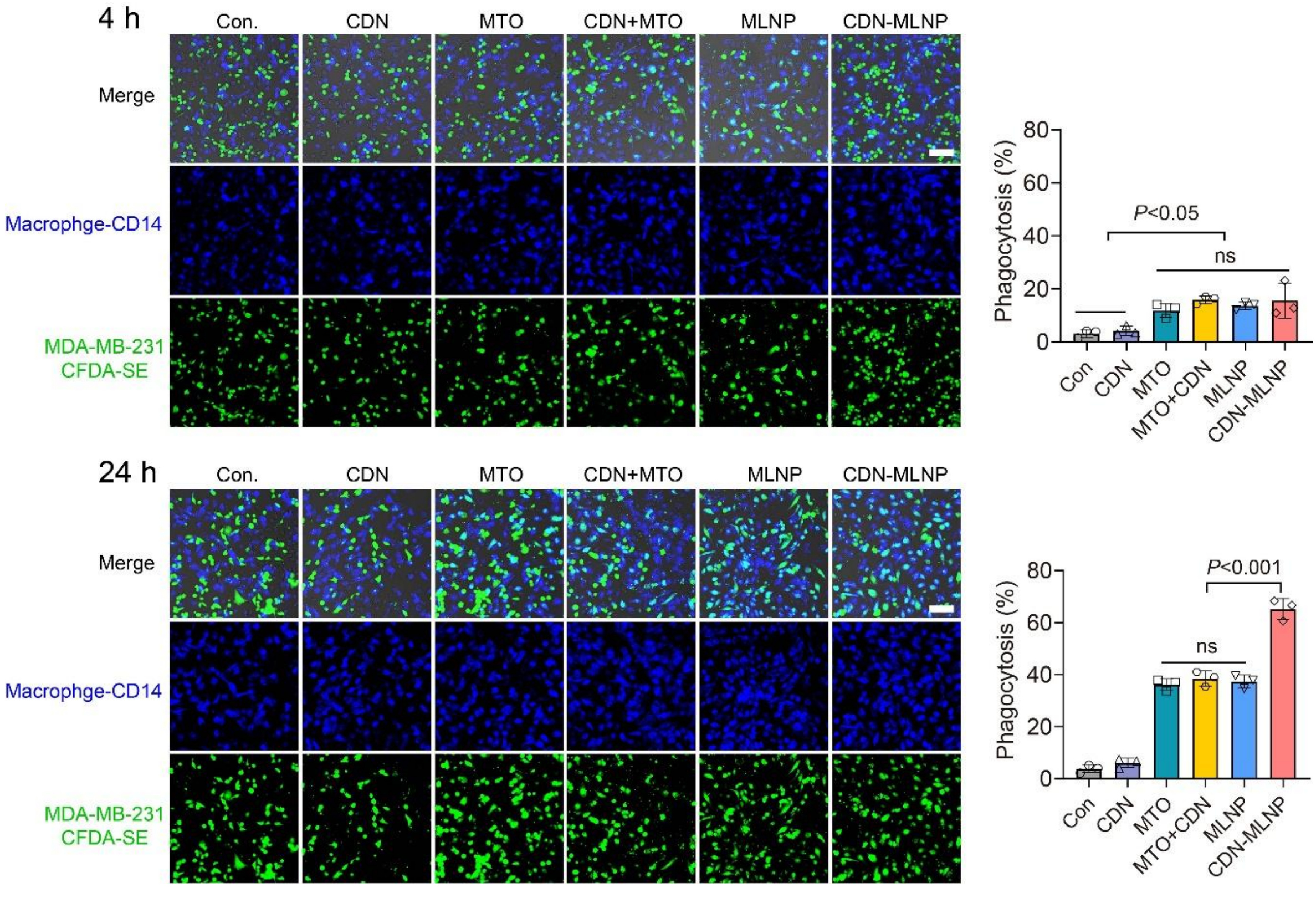

Figure S21. These confocal images show the phagocytosis of MDA-MB-231 cells by human macrophages and the corresponding quantification. The MDA-MB-231 cells were treated with different agents for 4 or 24 h and labeled with CFDA-SE (green fluorescence). Then the human blood-derived macrophages were co-cultured with treated MDA-MB-231 cells for 4 hours and then stained with pacific blue-labeled CD14 antibody (blue fluorescence). The macrophages (blue) incorporating green fluorescent signals indicated they phagocytized the cancer cells. Scale bar 100 μm.

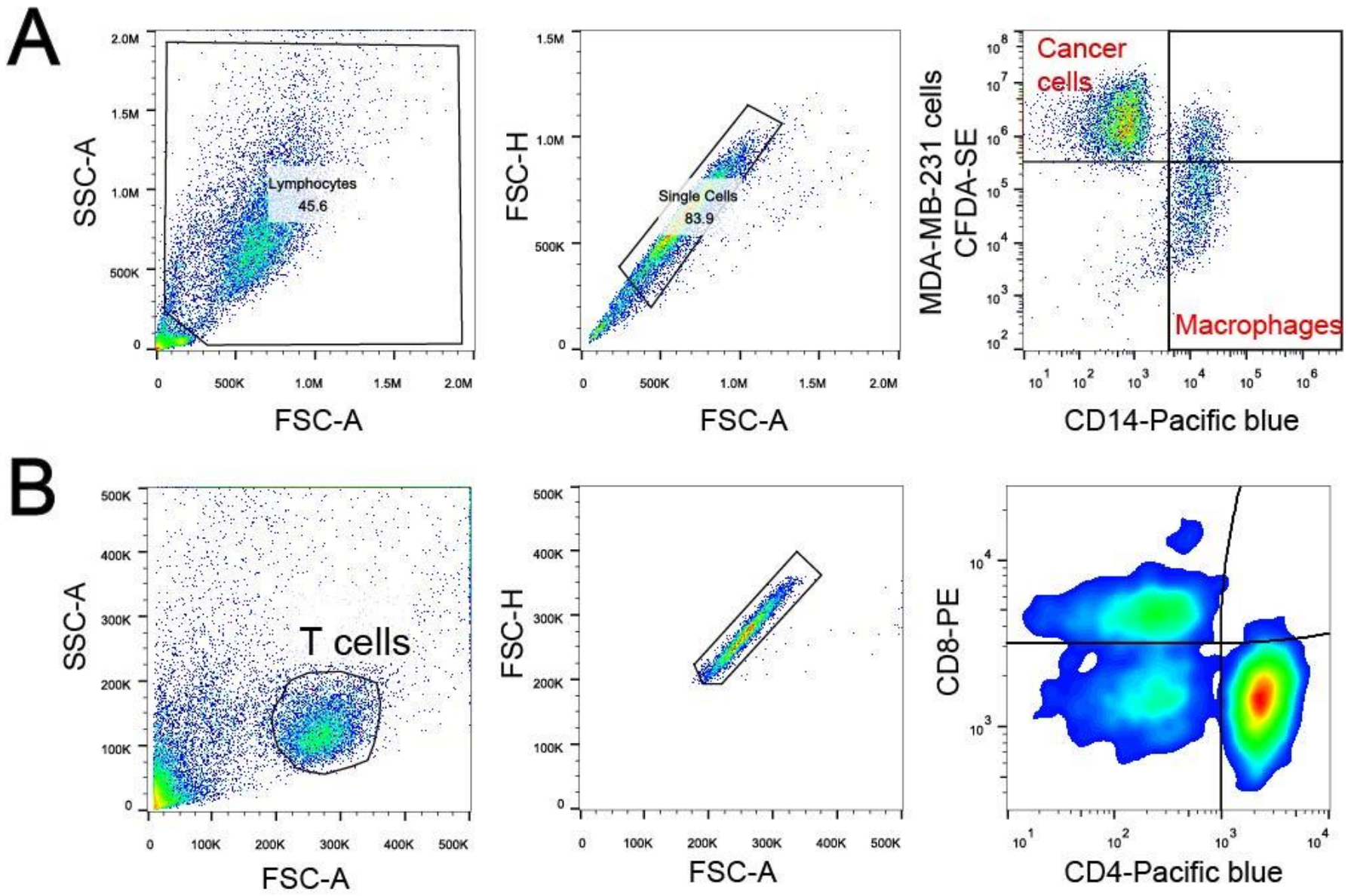

Figure S22. The flow cytometric gating strategies. A) The flow cytometric gating strategy for analyzing the phagocytosis of MDA-MB-231 cells by human macrophages. B) The flow cytometric gating strategy for analyzing T cells in co-culture systems of cancer cells and PBMCs. The cancer cells already treated with different agents for 24 h were used to co-culture with PBMCs for 48 h, and then the suspended cells were analyzed to determine the proportions of $CD4^+$ and $CD8^+$ cells using flow cytometry.

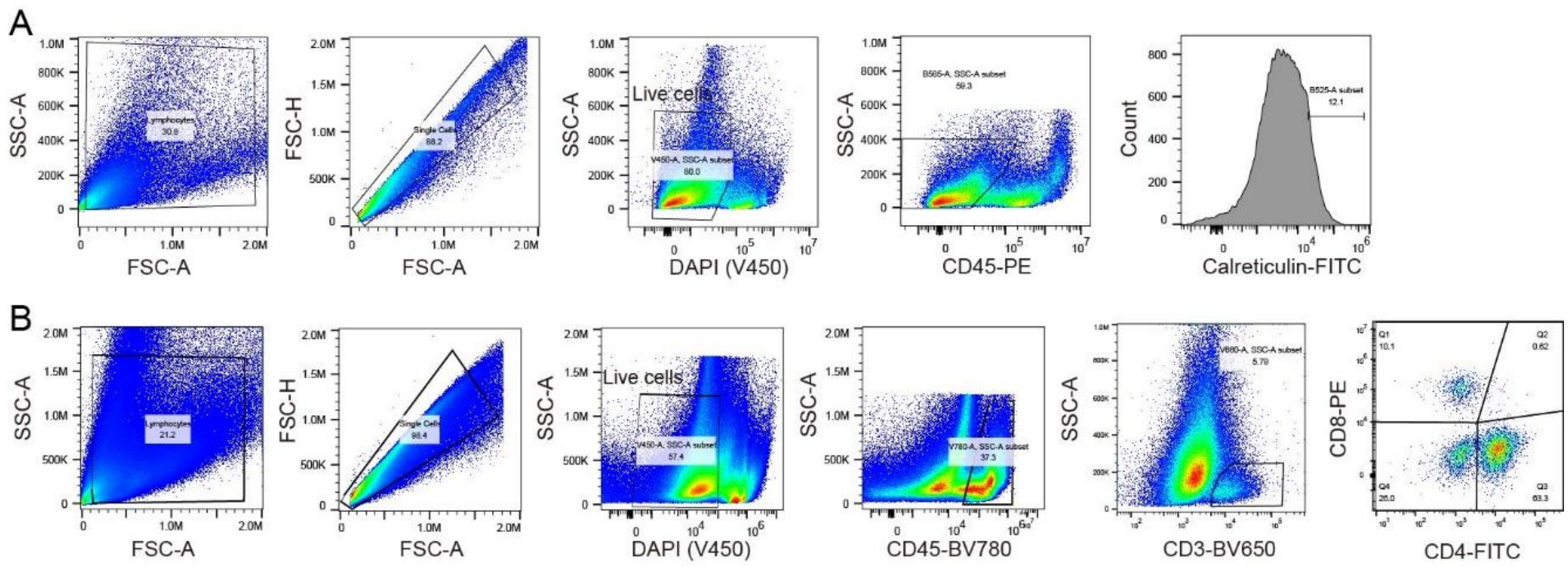

Figure S23. The flow cytometric gating strategies for analyzing the calreticulin (CRT)-positive cells (A) and T cells (B) in tumors of 4T1 tumor-bearing mice intratumorally injected with PBS, CDN, MTO, MTO+CDN, MLNP, CDN-LNP or CDN-MLNP.

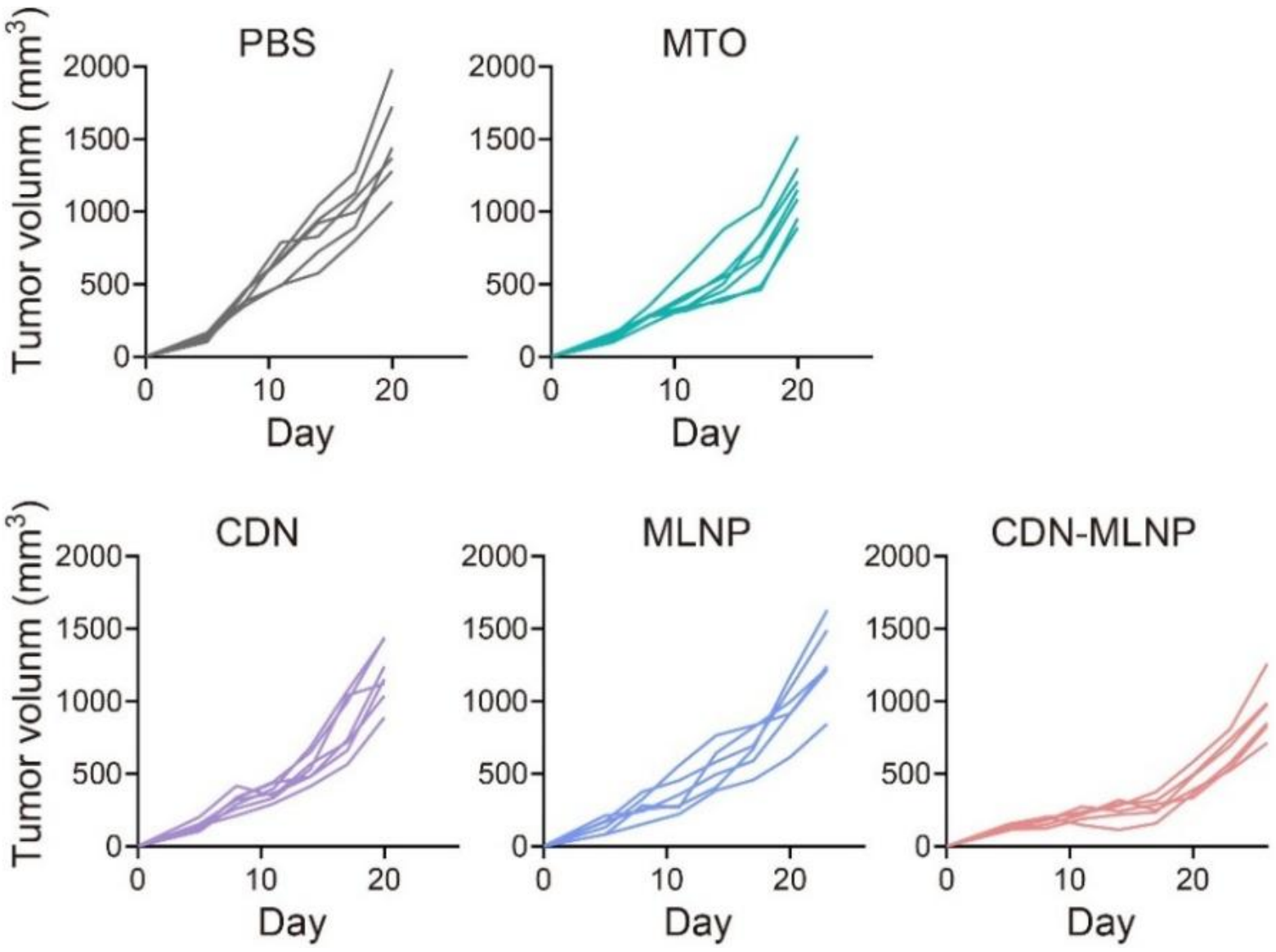

Figure S24. Individual tumor growth curves of 4T1 tumor-bearing mice of each group.

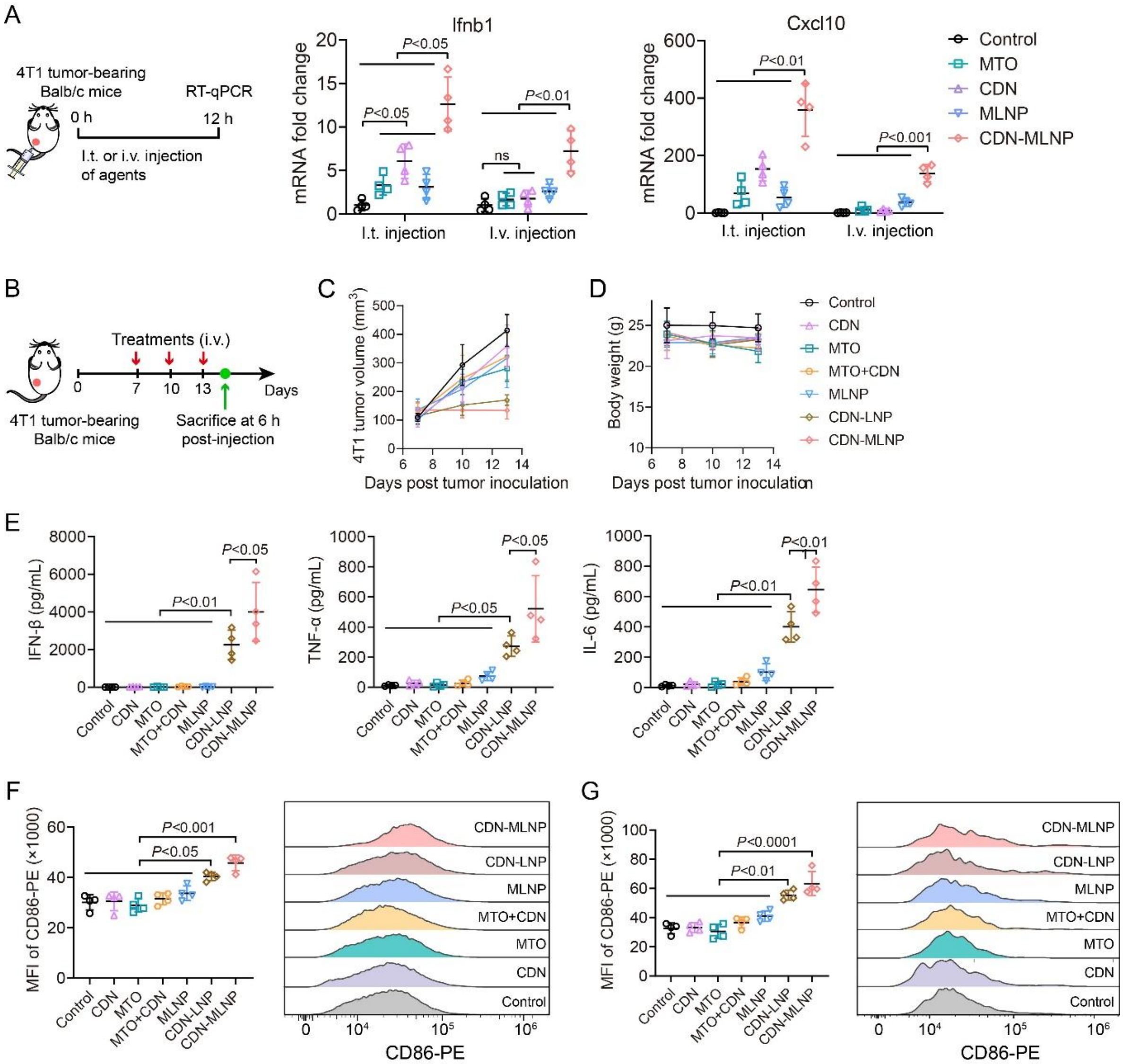

Figure S25. Induction of innate immune activation by CDN-MLNP in a mouse model of 4T1 breast cancer. (A) The 4T1 tumor-bearing Balb/c mice were intratumorally (i.t.) or intravenously injected of different agents, and tumors were collected 12 h later for RT-qPCR analysis. The relative mRNA levels of *Ifnb1* and *Cxcl10* in tumors were shown. The dose of each formulation for intravenous injection was the same with Figure 6 (Panel A), and doses for intratumoral injection are half of the doses in intravenous injection. n = 3 biologically independent samples. (B) 4T1 tumor-bearing Balb/c mice were treated with i.v. administration of different agents on day 7, 10 and 13, and sacrificed at 6 h after the third dose for subsequent investigations. (C) Tumor growth curves. (D) Body weight changes over the treatment period. (E) Serum cytokine levels were measured by ELISA following the third dose of treatment. (F and G) Quantification and representative flow cytometry histograms of CD86 expression on macrophages ($CD45^{+}F4/80^{+}$) (F) and DCs ($CD45^{+}F4/80^{-}CD11c^{+}$) (G) within the tumor. Data are presented as means ± s.d., with statistical significance calculated using one-way ANOVA test.

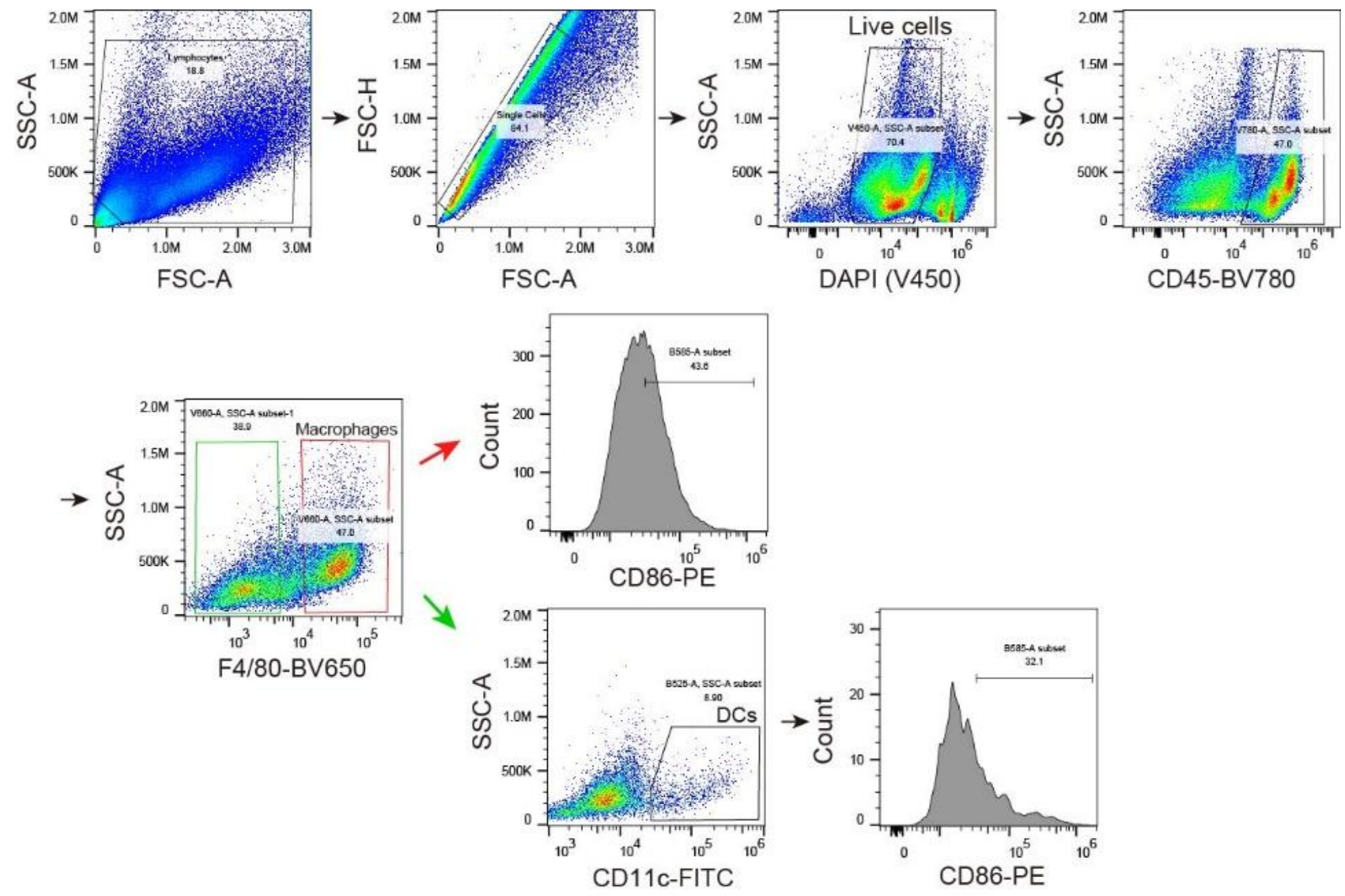

Figure S26. The gating strategy for analyzing the CD86 expression on macrophages and DCs in the 4T1 tumor-bearing mice.

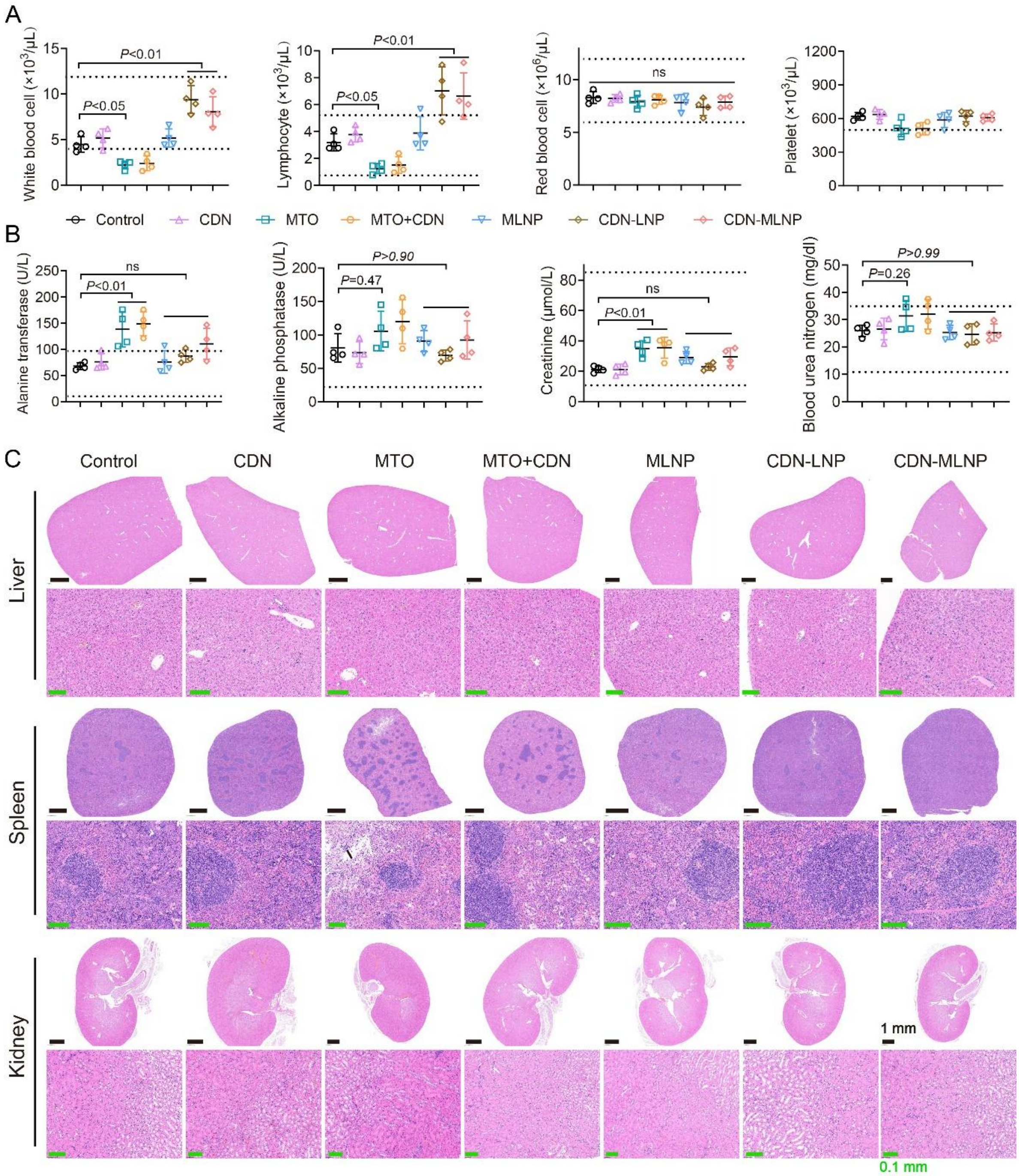

Figure S27. In vivo safety evaluation in 4T1 tumor-bearing mice. 4T1 tumor-bearing Balb/c mice were intravenously administered with control, CDN, MTO, MTO+CDN, MLNP, CDN-LNP, or CDN-MLNP on days 7, 10, and 13 post-tumor inoculation (n = 4 per group). Blood and tissues were collected 6 h after the third dose. (A) Complete blood counts: white blood cells (WBC), lymphocytes, red blood cells (RBC), and platelets. Normal reference ranges are indicated by dotted line areas. (B) Serum biochemistry: alanine aminotransferase (ALT), alkaline phosphatase (ALP), creatinine (CR), and blood urea nitrogen (BUN). Reference ranges are shown as dotted line areas. (C) H&E staining of liver, spleen and kidney sections.

Data are presented as means ± s.d., with statistical significance calculated using one-way ANOVA test.

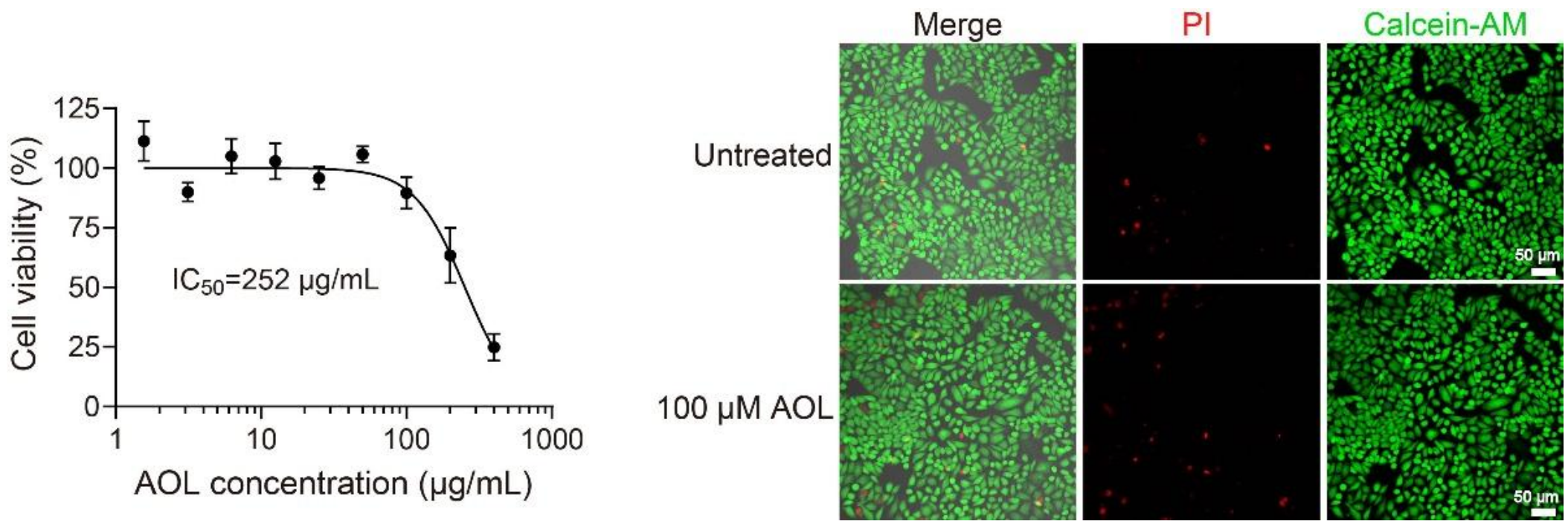

Figure S28. In vitro cytotoxicity of the amine oxide lipid (AOL) on human umbilical vein endothelial cells (HUVECs). HUVECs were treated with various concentrations of AOL for 24 h. Cell viability was assessed by the CCK-8 assay (left panel), and live/dead staining was performed using calcein-AM (green, live cells) and propidium iodide (red, dead cells) with confocal images shown (right panel).

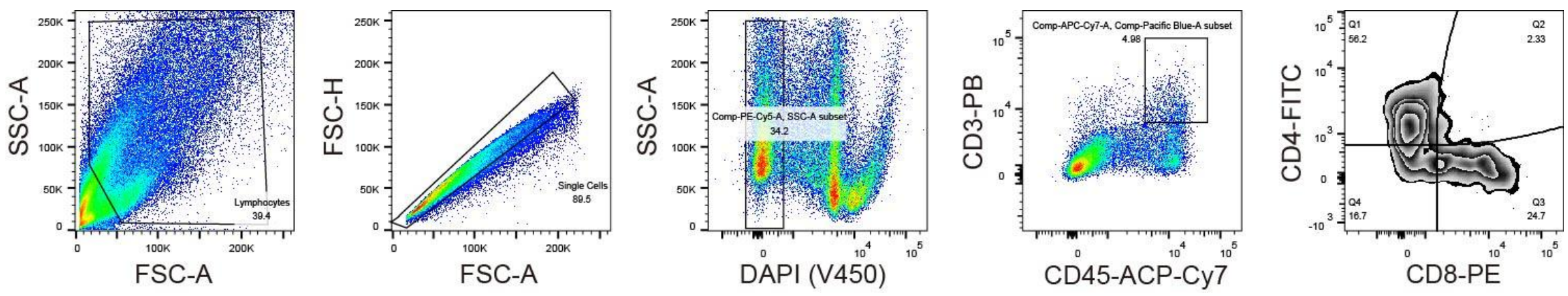

Figure S29. The flow cytometric gating strategy for analyzing the $CD4^+$ and $CD8^+$ T cells in the KPC PDAC tumors.

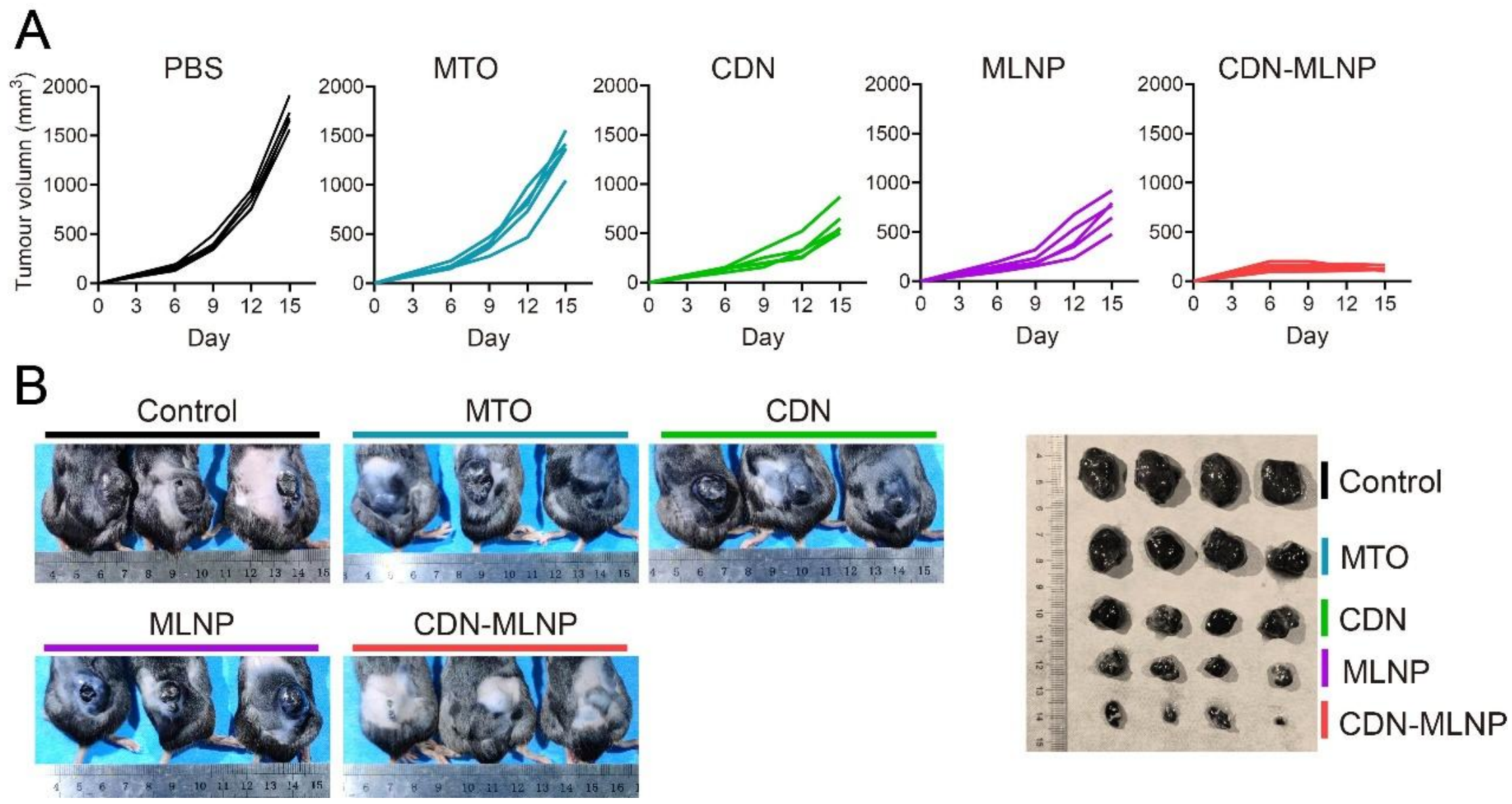

Figure S30. The therapeutic efficacy of CDN-MLNP and other formulations to B16-F10 tumor-bearing mice receiving two i.v. doses. (A) Individual tumor growth curves of the mice of each group. (B) Photos of B16-F10 tumor-bearing mice before the tumor excision (day 15 in Fig. 6J), and the image of isolated tumors at the end of treatments.

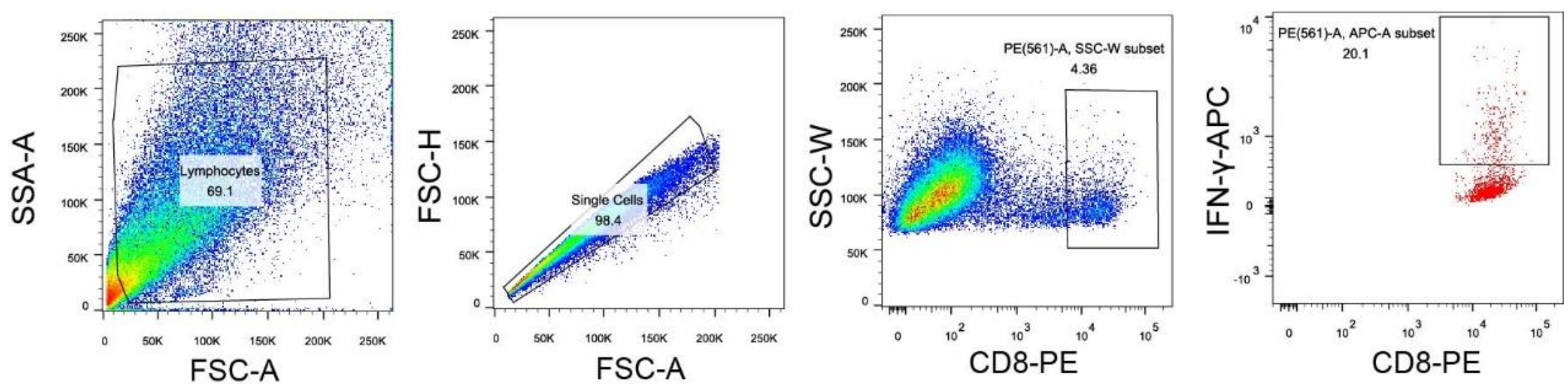

Figure S31. The flow cytometric gating strategy for analyzing the $CD8^{+}IFN\gamma^{+}$ T cells in the B16-F10 tumors.

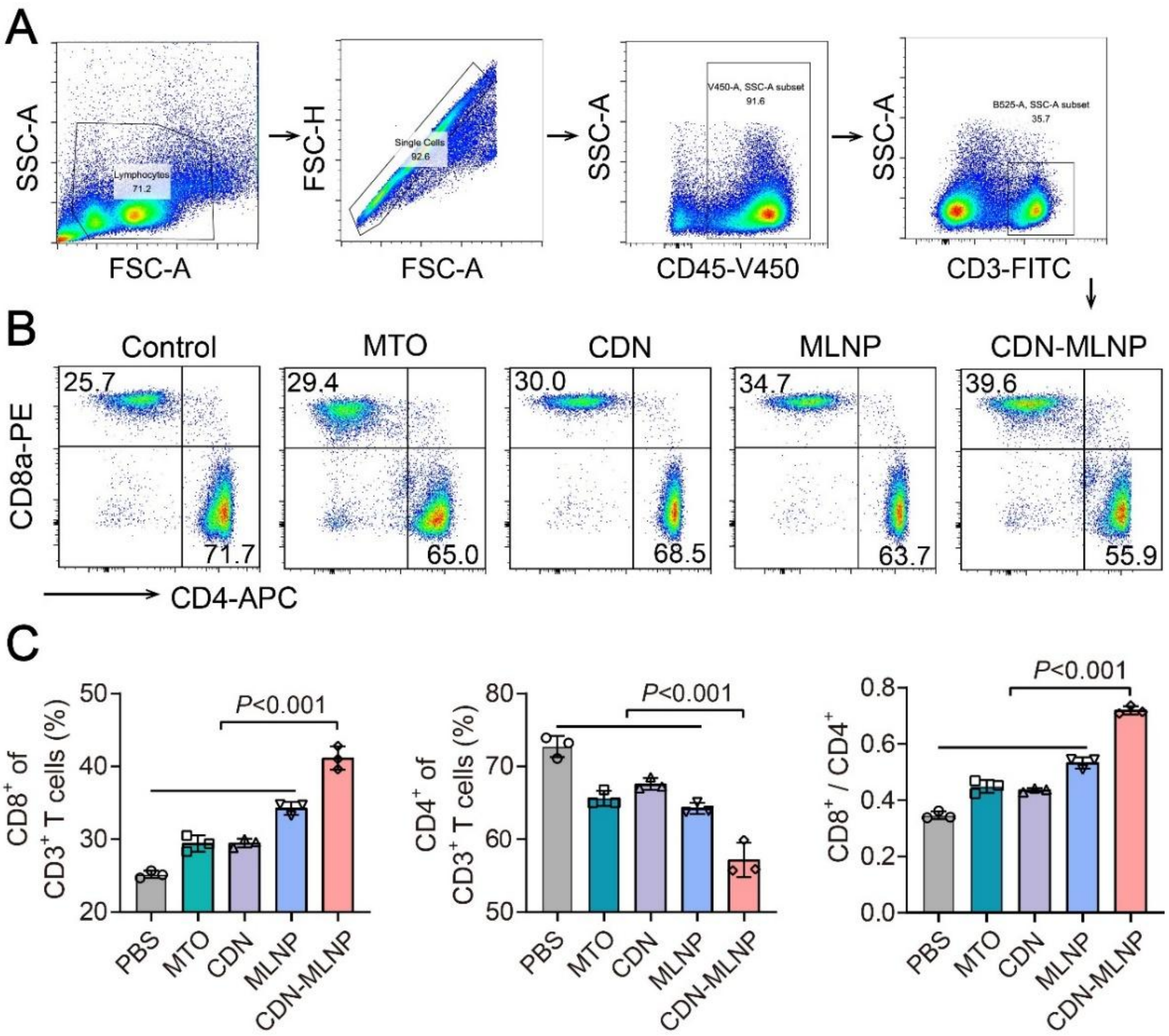

Figure S32. Flow cytometry analysis of spleen T cells in the B16-F10 tumor-bearing mice when the mice were sacrificed (day 50). (A) The flow cytometric gating strategy for analyzing T cells in the spleen of B16-F10 tumors. (B and C) Flow cytometry cytometric plots (B) and corresponding quantification (C) of $CD8^+$ T or $CD4^+$ T cells (in $CD3^+$ T cells) in mouse spleens in each group. Data are representative from three biological independent samples and are shown as mean ± s.d..

Table S1. Primers for real-time quantitative PCR

Name	Sequence
H-IFNb1 forward	5'-CTCCTCCAAATTGCTCTCCTGT-3'
H-IFNb1 reverse	5'-CTCATTCCAGCCAGTGCTAGAT-3'
H-CXCL10 forward	5'-TCCAGTCTCAGCACCATGAATC-3'
H-CXCL10 reverse	5'-AACACGTGGACAAAATTGGCTT-3'
H-CXCL9 forward	5'-GTTCTGATTGGAGTGCAAGGAAC-3'
H-CXCL9 reverse	5'-TCACATCTGCTGAATCTGGGTTT-3'
M-IFNb1 forward	5'-GGCTTCCATCATGAACAACAGG-3'
M-IFNb1 reverse	5'-GATGGCAAAGGCAGTGTAACTC-3'
M-CXCL10 forward	5'-GCCGTCATTTTCTGCCTCA-3'
M-CXCL10 reverse	5'-CGTCCTTGCGAGAGGGATC-3'
M-CXCL9 forward	5'-TGGGCAGAAGTTCCGTCTTG-3'
M-CXCL9 reverse	5'-ATTACCGAAGGGAGGTGGACAACG-3'
H-Gapdh forward	5'-GAGTCAACGGATTTGGTCGT-3'
H-Gapdh reverse	5'-GACAAGCTTCCCGTTCTCAG-3'
M-Gapdh forward	5'-GTCATCCCAGAGCTGAACG-3'
M-Gapdh reverse	5'-TCATACTTGGCAGGTTTCTCC-3'
